# Intraoperative Wound Irrigation for the Prevention of Surgical Site Infection After Laparotomy: A Randomized Clinical Trial by CHIR-Net

**DOI:** 10.1001/jamasurg.2023.7985

**Published:** 2024-02-21

**Authors:** Tara Catharina Mueller, Victoria Kehl, Rebekka Dimpel, Christiane Blankenstein, Silvia Egert-Schwender, Judith Strudthoff, Johan Friso Lock, Armin Wiegering, Ali Hadian, Hauke Lang, Markus Albertsmeier, Michael Neuberger, Viktor Von Ehrlich-Treuenstätt, André L. Mihaljevic, Phillip Knebel, Frank Pianka, Chris Braumann, Waldemar Uhl, Ralf Bouchard, Ekaterina Petrova, Ulrich Bork, Marius Distler, Michael Tachezy, Jakob R. Izbicki, Christoph Reissfelder, Florian Herrle, Christian Vay, Wolfram Trudo Knoefel, Alexander Buia, Ernst Hanisch, Helmut Friess, Daniel Reim

**Affiliations:** 1 Department of Surgery, School of Medicine and Health, Technical University of Munich, Munich, Germany; 2 Münchner Studienzentrum, School of Medicine and Health, Technical University of Munich, Munich, Germany; 3 Department of General, Visceral, Transplant, Vascular and Pediatric Surgery, University Hospital of Würzburg, Würzburg, Germany; 4 Department of General, Visceral and Transplant Surgery, University Medical Center Mainz, Mainz, Germany; 5 Department of General, Visceral and Transplantation Surgery, Ludwig Maximilians University of Munich, University Hospital, Munich, Germany; 6 Clinical Study Center, Department of General, Visceral and Transplantation Surgery, Heidelberg University Hospital, Heidelberg, Germany; 7 Department of General and Visceral Surgery, St. Josef Hospital, Ruhr University Bochum Hospitals, Bochum, Germany; 8 Department of Surgery, University Medical Center Schleswig-Holstein, Campus Lübeck, Lübeck, Germany; 9 Department of Visceral, Thoracic and Vascular Surgery, Faculty of Medicine and University Hospital Carl Gustav Carus, Technische Universität Dresden, Dresden, Germany; 10 National Center for Tumor Diseases, Dresden, Germany: German Cancer Research Center, Heidelberg; Faculty of Medicine and University Hospital Carl Gustav Carus, Technical University of Dresden, Helmholtz Center Dresden-Rossendorf, Dresden, Germany; 11 Department of General, Visceral and Thoracic Surgery, University Hospital Hamburg-Eppendorf, Hamburg, Germany; 12 Department of Surgery, University Medical Center Mannheim, Medical Faculty Mannheim, Heidelberg University, Mannheim, Germany; 13 Department of General, Visceral, Thoracic, and Pediatric Surgery, Heinrich Heine University Düsseldorf, Düsseldorf, Germany; 14 Asklepios Clinic Langen, Department of General, Visceral and Thoracic Surgery, Langen, Germany

## Abstract

**Importance:**

Surgical site infections frequently occur after open abdominal surgery. Intraoperative wound irrigation as a preventive measure is a common practice worldwide, although evidence supporting this practice is lacking.

**Objective:**

To evaluate the preventive effect of intraoperative wound irrigation with polyhexanide solution.

**Design, Setting, and Participants:**

The Intraoperative Wound Irrigation to Prevent Surgical Site Infection After Laparotomy (IOWISI) trial was a multicenter, 3-armed, randomized clinical trial. Patients and outcome assessors were blinded to the intervention. The clinical trial was conducted in 12 university and general hospitals in Germany from September 2017 to December 2021 with 30-day follow-up. Adult patients undergoing laparotomy were eligible for inclusion. The main exclusion criteria were clean laparoscopic procedures and the inability to provide consent. Of 11 700 screened, 689 were included and 557 completed the trial; 689 were included in the intention-to-treat and safety analysis.

**Interventions:**

Randomization was performed online (3:3:1 allocation) to polyhexanide 0.04%, saline, or no irrigation (control) of the operative wound before closure.

**Main Outcome and Measures:**

The primary end point was surgical site infection within 30 postoperative days according to the US Centers for Disease Control and Prevention definition.

**Results:**

Among the 689 patients included, 402 were male and 287 were female. The median (range) age was 65.9 (18.5-94.9) years. Participants were randomized to either wound irrigation with polyhexanide (n = 292), saline (n = 295), or no irrigation (n = 102). The procedures were classified as clean contaminated in 92 cases (8%). The surgical site infection incidence was 11.8% overall (81 of 689), 10.6% in the polyhexanide arm (31 of 292), 12.5% in the saline arm (37 of 295), and 12.8% in the no irrigation arm (13 of 102). Irrigation with polyhexanide was not statistically superior to no irrigation or saline irrigation (hazard ratio [HR], 1.23; 95% CI, 0.64-2.36 vs HR, 1.19; 95% CI, 0.74-1.94; *P* = .47). The incidence of serious adverse events did not differ among the 3 groups.

**Conclusions and Relevance:**

In this study, intraoperative wound irrigation with polyhexanide solution did not reduce surgical site infection incidence in clean-contaminated open abdominal surgical procedures compared to saline or no irrigation. More clinical trials are warranted to evaluate the potential benefit in contaminated and septic procedures, including the emergency setting.

**Trial Registration:**

drks.de Identifier: DRKS00012251

## Key Points

**Question:**

Is prophylactic intraoperative wound irrigation with polyhexanide 0.04% solution effective in reducing surgical site infections after laparotomy?

**Findings:**

This randomized clinical trial did not find a significant difference in surgical site infection rates between intraoperative wound irrigation with polyhexanide compared to saline or no irrigation in 689 patients undergoing laparotomy.

**Meaning:**

According to these findings, intraoperative wound irrigation with polyhexanide solution should not be recommended as standard clinical practice in open clean-contaminated surgical procedures; additional clinical trials are warranted to evaluate the potential benefit in contaminated and septic procedures, including the emergency setting.

## Teaser

This randomized clinical trial assesses the effect of intraoperative wound irrigation in preventing surgical site infection.

## Introduction

According to the GlobalSurg Collaborative,^1,2^ postoperative surgical site infection is the most common type of hospital-acquired infection across all income and development settings after gastrointestinal surgery. Surgical site infections prolong the length of hospital stay and increase costs and morbidity and mortality rates.^3,4,5^ While European surveillance data^5^ show surgical site infection rates of 10.1% after open colorectal surgery, randomized clinical trials^6,7,8^ have demonstrated an incidence of 15% to 30%, depending mainly on the intraoperative level of contamination according to the US Centers for Disease Control and Prevention classification (eAppendix 1 in Supplement 2). Most patient-related risk factors for surgical site infection, such as age, obesity, diabetes, and comorbidities, are hardly modifiable. Therefore, the optimization of surgery-related factors to reduce intraoperative contamination by using sterile techniques and application of antiseptic or antibiotic substances has been the focus of current research. Intraoperative wound irrigation of the surgical incision at the end of surgery is widely practiced, and a multitude of antiseptic irrigation solutions are available, although evidence-based recommendations are inconsistent. While the British National Institute for Health and Care Excellence guideline (2019) recommends against intraoperative wound irrigation due to lack of evidence,^9^ the World Health Organization (2016) and the US Centers for Disease Control and Prevention (2017) guidelines recommend consideration of intraoperative wound irrigation with aqueous iodophor (povidone-iodine) solution for surgical site infection prevention^10,11,12^ (eAppendix 2 in the Supplement 2). Most abdominal surgery clinical trials are outdated (1970 to 1990) and demonstrate high risk of bias.^12^ A large systematic review and meta-analysis^13^ included all kinds of surgery and compared, nonantibacterial, antiseptic, and antibiotic irrigation solutions to no irrigation. The mean odds ratio of surgical site infection was significantly lower for antibiotic and antiseptic irrigations, and no irrigation was comparable to nonantibacterial irrigation.

Polyhexanide solutions are relatively new, and data from in vitro and in vivo trials have shown favorable prophylactic and therapeutic antiseptic effects. A wound antisepsis consensus (2018)^14^ stated that polyhexanide was the agent of choice for critically colonized infected chronic and burn wounds. Even though evidence from trials evaluating prophylactic polyhexanide application is scarce, many surgeons use it in clinical practice because of its broad antimicrobial effect, long postantiseptic effect, anti-inflammatory properties, and reduction of biofilm and fibrin depositions. However, a pilot trial^15^ in colorectal procedures (laparoscopic and open) found no significant difference in surgical site infection rates between intraoperative wound irrigation with polyhexanide and Ringer solution. In contrast, the recent Reduction of Postoperative Wound Infections by Antiseptica (RECIPE) trial^8^ demonstrated a significant surgical site infection reduction after polyhexanide intraoperative wound irrigation. Thus, the multicenter Intraoperative Wound Irrigation to Prevent Surgical Site Infection After Laparotomy (IOWISI) trial aimed to determine whether intraoperative wound irrigation with polyhexanide is effective in surgical site infection reduction within 30 days after open gastrointestinal surgery compared to saline or no irrigation.

## Methods

### Study Design

The IOWISI trial was a prospective, observer- and patient-blinded, multicenter randomized clinical trial with 3 parallel comparison groups. The trial is reported in accordance with Consolidated Standards of Reporting Trials (CONSORT) reporting guideline.^16^ Patients were randomized to either no intraoperative wound irrigation, saline 0.9% intraoperative wound irrigation, or polyhexanide 0.04% intraoperative wound irrigation at a ratio of 1:3:3. The trial was conducted at 12 surgical sites within the Surgical Trial Network (CHIR-Net) in Germany. Each participating site obtained local ethics committee approval. The trial protocol was approved by the leading ethics committee of the Technical University of Munich and by the Federal Institute for Drugs and Medical Devices. The trial was registered at drks.de (DRKS00012251). The published protocol^17^ is presented in Supplement 1. The study took place from September 2017 to December 2021, with 30-day follow-up. All participants provided written informed consent.

### Participants

Patients undergoing elective and emergency visceral laparotomy (transverse or midline) were screened. Main exclusion criteria were level of contamination I, laparoscopic or robotic surgery, surgery without opening of the abdominal cavity, revision surgery (previous abdominal surgery within 30 days before trial inclusion or planned repeat laparotomy within 30 days after trial inclusion), preoperative continuous antibiotic therapy within 5 days before surgery, American Society of Anesthesiologists score of 3, severe immunosuppression (after organ or bone marrow transplantation; concurrent steroid treatment with >10-mg prednisone equivalent daily, and infliximab treatment or an equivalent immunosuppressive), chemotherapy within the last 2 weeks prior to inclusion, severe preoperative neutropenia (≤0.5 × 10^9^/L) or liver cirrhosis (Child-Pugh score of B/C), pregnancy or breastfeeding, and known hypersensitivity or allergy to polyhexanide. Routine intraoperative antibiotic prophylaxis (single shot and redosing) was not an exclusion criterion. Patients had to be 18 years old or older and provide written informed consent. Patients had to be able and willing to attend follow-up visits.

### Randomization and Masking

A member of the study group not involved in the postoperative evaluation of patients performed intraoperative randomization after complete abdominal fascia closure by an online randomization tool using predefined randomization lists, which were created by the Münchner Studienzentrum. To assure balanced group sizes during the accrual, block-wise randomization was applied. The allocation sequence was concealed to study group members performing the randomization. The blinding procedure was restricted to participants, outcome assessors, and the trial statistician. Blinding of the surgical team performing the intervention was not possible because the control arm did not receive any wound irrigation. Postoperatively, blinded investigators conducted assessment of the primary end point.

### Procedures

After randomization, patients received intraoperative wound irrigation with 1000 mL of 0.04% polyhexanide solution or 1000 mL saline or no irrigation. The wound was rinsed with the respective solution and the excess was removed by suction. Debris and blood clots were removed from the wound using irrigation and suction. The volume of 1000 mL was chosen by common practice, to make sure enough solution was available for large or deep laparotomy wounds or repeated irrigation if necessary. The wound was left moistened with the irrigation solution to ensure sufficient contact time (>10 minutes). After intraoperative wound irrigation, skin closure was performed according to local standards without any further wound-related procedure.

### Outcomes

The primary efficacy end point was incidence of surgical site infection grade I to III within 30 days postoperatively.^18^ The following secondary end points were assessed within 30 days postoperatively: length of hospital stay, reoperation rate, noninfectious wound complication rate, adverse event and serious adverse event rates, mortality, and morbidity. All adverse events and serious adverse events related to surgery were categorized according to the Clavien-Dindo classification.^19^

### Statistical Analysis

#### Sample Size

The initial sample size was calculated in nQuery 2017 version 7.0 (Statistical Solutions) assuming surgical site infection rates of 2.2% in the polyhexanide group (anticipating a 75% risk reduction according to Roth^20^), 8.7% in the saline group (according to Cervantes-Sanchez^21^), and 16.2% in the control group (according to Mueller^12^). The global significance level was 5%. Since the polyhexanide arm was used twice for comparison, the Bonferroni-Holm procedure was used to set the local α level for polyhexanide vs no intervention (test 1) at 2.5% and for polyhexanide irrigation vs saline irrigation (test 2) to 5%. This resulted in a planned sample size of 230 patients in the polyhexanide arm, 230 patients in the saline arm, and 80 patients in the control arm (N = 540). The 2-sided Fisher exact test had a power to detect differences between the treatment groups of 94% for test 1 and 85% for test 2. For this initial calculation a dropout-rate of 8% to 10% was assumed, based on data from comparable trials.^6^ However, during the IOWISI trial, a much higher dropout rate of 21% occurred, related to a higher than expected emergency relaparotomy rate after patient inclusion. Therefore, a sample size reestimation was required. The analysis was now based on a Fine-Gray subdistributional hazard model with surgical site infection as the main event and relaparotomy and death as competing risks. All other missing surgical site infections were censored at the time of last follow-up. The sample size was then adjusted from 540 to 680 patients (290 + 290 + 100) applying the initial assumptions for the expected surgical site infection rates (event of interest).^22^ The incidence rate for the competing risks of death or relaparotomy was estimated to be a total of 13.4% in all arms. The 2 tests of interest from the Fine-Gray model had an estimated power of 80% each to detect differences between the treatments. These changes to the study protocol were approved by the local ethics committee and federal health authority.

#### Statistical Methods

The primary and secondary end points were analyzed on the intention-to-treat set, consisting of all patients included in the treatment arm to which they were randomized. The safety analysis was performed in all patients according to their actual treatment. Polyhexanide intraoperative wound irrigation was tested for superiority over no intraoperative wound irrigation and saline intraoperative wound irrigation using a Fine-Gray subdistributional hazard model with surgical site infection as the main event; relaparotomy and death as competing risks; and treatment group, level of contamination, and body mass index (BMI) as covariates. The covariate trial center was retrospectively excluded from the model, as this stratification was done for administrative reasons only and some centers recruited low numbers of patients (<10). Instead, BMI was included in the model due to detection of baseline differences. The tests were 2-sided with a global significance level of 5%. Using Bonferroni-Holm adjustment, the local significance level was 2.5% and 5% in the order of increasing *P* value.

#### Methods for Subgroup Analyses and Adjusted Analyses

Sensitivity analysis of the primary end point was performed by including the following additional covariates in the Fine-Gray model: diabetes, National Nosocomial Infections Surveillance score, type and duration of surgery, intraoperative glove change, use of wound edge protectors, and enterostomy creation. Surgical site infection incidence, relaparotomy, and death were compared using χ^2^ or Fisher exact test. Secondary end points were analyzed by treatment group in the intention-to-treat set, using appropriate descriptive statistics. Any explorative statistical testing was performed using a 2-sided significance level of 5%. Subgroup analyses of the primary end point were performed by adding various surgical site infection risk factors as covariates to the model. To accommodate interest in surgical site infection grades I or II, which are more likely to be influenced by irrigation than surgical site infection grade III, surgical site infection grades I and II were combined as the main event and surgical site infection grade III, relaparotomy, and death as competing risks. Adverse events were analyzed in the safety set according to participants’ actual treatment group, severity, and relationship to the type of irrigation. Missing primary end point data in the primary analysis were dealt with by means of competing risks and censoring. Percentages reflect the number of known values in the denominator, unless otherwise stated. All safety analyses were performed as treated; all other analyses were performed as randomized.

## Results

A total of 11 700 patients were prescreened. Most prescreened patients (n = 10 390) met 1 or more of the major exclusion criteria, and the remaining 1310 were asked for informed consent (Figure). Finally, 689 patients were included in the study (402 male and 287 female; median [range] age, 65.9 [18.5-94.9] years), 292 of whom were randomized to the polyhexanide arm, 295 to the saline arm, and 102 to the no irrigation arm. All 689 patients were included in the analyses, although 132 patients (19.2%) discontinued the study prematurely due to adverse events (n = 77 [11.9%]), withdrawal of consent (n = 11 [1.6%]), lost to follow-up (n = 20 [2.9%]), death (n = 11 [1.6%]), or other reasons (n = 13 [2.2%]). The study was completed by 557 patients (80.8%): 242 of 292 (82.9%) in the polyhexanide group, 238 of 295 (80.7%) in the saline group, and 77 of 102 (75.5%) in the no irrigation group.

**Figure.**
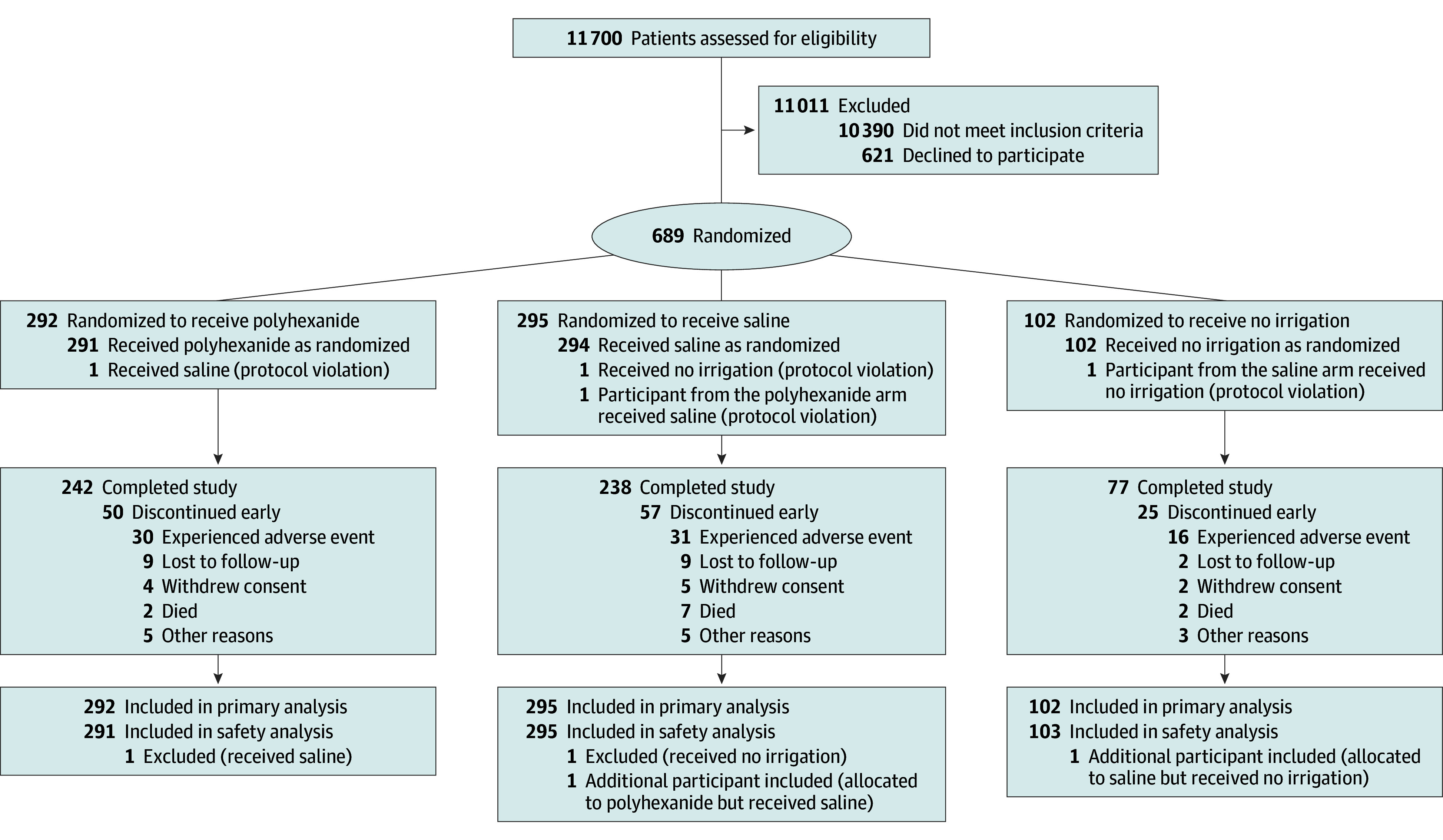
CONSORT 2010 Flow Diagram for the Intraoperative Wound Irrigation to Prevent Surgical Site Infection After Laparotomy (IOWISI) Trial^16^

The polyhexanide group included 126 women (43.2%), the saline group 124 women (42.0%), and the no irrigation group 37 women (36.3%). The median (range) age was 66.1 (20-88) years in the polyhexanide group, 66.4 (18-90) years in the saline group, and 63.3 (23-95) years in the no irrigation group. Except for BMI, all other baseline factors were evenly distributed (Table 1). The most frequent type of surgery was hepatobiliary-pancreatic (411 of 689 [59.7%]), followed by colorectal (129 or 689 [18.7%]) and upper gastrointestinal (127 of 689 [18.4%]). One procedure in each of the 2 intervention groups was classified as level of contamination I due to early randomization. Two procedures in the polyhexanide group were classified as level of contamination IV. Most of the procedures were graded level of contamination II: 271 of 292 (92.8%) in the polyhexanide group, 272 of 295 (92.2%) in the saline group, and 94 of 102 (92.2%) in the no irrigation group. There were 48 patients with level of contamination III procedures: 18 of 292 (6.2%) in the polyhexanide group, 22 of 295 (7.5%) in the saline group, and 8 of 102 (7.8%) in the no irrigation group. Wound edge protectors or glove changes were used in approximately 30% and 60% of cases (Table 1).

**Table 1.** Baseline Characteristics of Patients Undergoing Laparotomy by Wound Irrigation Groups

Characteristic	Treatment group, No. (%)		
	Polyhexanide (n = 292)	Saline (n = 295)	No irrigation (n = 102)
Patient-related factors			
Sex			
Female	126 (43.2)	124 (42.0)	37 (36.3)
Male	166 (56.9)	171 (58.0)	65 (63.7)
Age, median (range), y	66.1 (20.4-88.5)	66.4 (18.5-89.5)	63.3 (23.1-94.9)
BMI			
<18.5	3 (1.0)	8 (2.7)	8 (7.8)
18.5-<25	145 (49.7)	125 (42.4)	39 (38.2)
25-<30	94 (32.2)	110 (37.3)	39 (38.2)
≥30	50 (17.1)	52 (17.6)	16 (15.7)
Smoking			
No	192 (65.8)	205 (69.5)	63 (61.8)
Yes, currently	45 (15.4)	47 (15.9)	18 (17.7)
Yes, previously	55 (18.8)	43 (14.6)	21 (20.6)
Alcohol consumption			
No	214 (73.3)	218 (73.9)	74 (72.6)
Yes, currently	69 (23.6)	61 (20.1)	25 (24.5)
Yes, previously	9 (3.1)	16 (5.4)	3 (2.9)
ASA score			
1	19 (6.5)	17 (5.8)	5 (4.9)
2	122 (41.8)	126 (42.7)	44 (43.1)
3	151 (51.7)	152 (51.5)	53 (52.0)
Comorbidities			
No. of ongoing comorbidities			
0-1	274 (93.8)	268 (90.8)	96 (94.1)
2-3	16 (5.5)	24 (8.1)	6 (5.9)
>3	2 (0.7)	3 (1.0)	0
Diabetes	54 (18.5)	59 (20.0)	21 (20.6)
Cardiovascular disease	131 (44.9)	138 (46.8)	54 (52.9)
Pulmonary disease	28 (9.6)	37 (12.5)	19 (18.6)
Kidney disease	22 (7.5)	19 (6.4)	8 (7.8)
Malignant disease	231 (79.1)	238 (80.7)	83 (81.4)
History of radiotherapy	31 (10.6)	39 (13.2)	12 (11.8)
History of chemotherapy	83 (28.4)	99 (33.6)	33 (32.4)
Preoperative hospital stay >2d	41 (14.0)	57 (19.3)	17 (16.7)
Previous abdominal surgery	175 (59.9)	200 (67.8)	65 (63.7)
History of surgical site infection, No./total No.	17/175 (9.7)	11/200 (5.5)	9/65 (13.8)
Operation-related factors			
NNIS risk score			
0	87 (30.4)	81 (28.3)	24 (24.5)
1	131 (45.8)	133 (46.5)	48 (49.0)
2	61 (21.3)	66 (23.1)	25 (25.5)
3	7 (2.4)	6 (2.1)	1 (1.1)
Level of contamination			
I	1 (0.3)	1 (0.3)	0
II	271 (92.8)	272 (92.2)	94 (92.2)
III	18 (6.2)	22 (7.5)	8 (7.8)
IV	0	2 (0.7)	0
Type of surgery			
Colorectal	56 (19.2)	52 (17.6)	21 (20.6)
Hepatobiliary-pancreatic	174 (59.6)	177 (60.0)	60 (58.8)
Upper gastrointestinal	54 (18.5)	55 (18.6)	18 (17.7)
Other	8 (2.7)	9 (3.1)	3 (2.9)
Duration of surgery, median (range), min	280 (42-583)	255 (47-652)	263 (40-629)
Intraoperative changing of gloves	180 (61.6)	181 (61.4)	64 (62.8)
Use of wound edge protector device	83 (28.4)	81 (27.5)	28 (27.5)
Enterostomy	16 (5.5)	24 (8.1)	8 (7.8)
Preoperative antibiotic prophylaxis	292 (100)	292 (99.0)	101 (99.0)

Abbreviations: ASA, American Society of Anesthesiologists; BMI, body mass index (calculated as weight in kilograms divided by height in meters squared); NNIS, National Nosocomial Infections Surveillance.

The frequency of surgical site infection (I-III) was 81 of 689 (11.8%) overall, 31 of 292 (10.6%) in the polyhexanide group, 37 of 295 (12.5%) in the saline, and 13 of 102 (12.8%) in the no irrigation group (Table 2). Intraoperative polyhexanide irrigation showed no statistically significant difference from either no irrigation (hazard ratio [HR], 1.23; 95% CI, 0.64-2.36; *P* = .54) or saline irrigation (HR, 1.19; 95% CI, 0.74-1.94; *P* = .47). The covariates level of contamination and BMI were accounted for in the Fine-Gray subdistributional hazard model (Table 3). An additional model including diabetes, comorbidities, National Nosocomial Infections Surveillance risk score, type and duration of surgery, intraoperative glove change, application of wound edge protectors, and creation of an enterostomy was calculated as a sensitivity analysis of the primary end point. The HRs and *P* values for the respective treatment groups changed only minimally without influence on the main outcome. None of the covariates in the model were statistically significant (eTables 1 and 2 in Supplement 2).

**Table 2.** Secondary End Points by Wound Irrigation Group

End point	Treatment group, No. (%)			*P* value^a^	
	Polyhexanide (n = 292)	Saline (n = 295)	No irrigation (n = 102)	Polyhexanide vs no irrigation	Polyhexanide vs saline
Surgical site infection	31 (10.6)	37 (12.5)	13 (12.8)	.58	.52
Type of surgical site infection				.51^b^	.70^b^
None	254 (87.0)	248 (84.1)	85 (83.3)	NA	NA
Superficial	15 (5.1)	22 (7.5)	9 (8.8)	NA	NA
Deep	7 (2.4)	6 (2.0)	1 (1.0)	NA	NA
Organ/space	9 (3.1)	9 (3.1)	3 (2.9)	NA	NA
Missing	7 (2.4)	10 (3.4)	4 (3.9)	NA	NA
Relaparotomy	27 (9.2)	31 (10.5)	15 (14.7)	.12	.61
30-d Mortality	2 (0.7)	6 (2.0)	2 (2.0)	.28^b^	.29^b^
Noninfectious wound complications	53 (18.2)	51 (17.3)	21 (20.1)	.54	.83
Seroma	12 (4.1)	11 (3.7)	3 (2.9)	.77^b^	.83
Hematoma	14 (4.8)	14 (4.8)	5 (4.9)	>.99^b^	>.99^b^
Delayed wound healing	19 (6.5)	17 (5.8)	6 (5.9)	.85	.73
Necrosis	0	2 (0.7)	1 (1.0)	.26^b^	.16
Other	15 (5.1)	15 (5.1)	8 (7.8)	.30	>.99
Duration of hospital stay, median (range), d	14.5 (4-45)	15 (5-41)	13 (5-46)	.56^c^	.23^c^
No. of AEs per patient, median (range)	2 (0-18)	2 (0-15)	1 (0-20)	.82^c^	.13^c^
No. of serious AEs per patient, median (range)	0 (0-8)	0 (0-5)	0 (0-7)	.29^c^	.52^c^

Abbreviations: AE, adverse event; NA, not applicable.

^a^ χ^2^ Test, unless indicated otherwise.

^b^ Fisher exact test.

^c^ Mann-Whitney *U* test.

**Table 3.** Analysis of the Primary End Point With the Subdistributional Fine-Gray Hazard Model^a^

Variable	HR (95% CI)	*P* value
Treatment group		
No irrigation vs polyhexanide	1.23 (0.64-2.36)	.54
Saline vs polyhexanide	1.19 (0.74-1.94)	.47
Level of contamination		
III or IV vs I or II	1.63 (0.76-3.49)	.21
BMI^b^		
18.5-<25	1.51 (0.38-5.95)	.56
25-<30	1.22 (0.31-4.82)	.77
≥30	1.14 (0.28-4.72)	.86

Abbreviations: BMI, body mass index (calculated as weight in kilograms divided by height in meters squared); HR, hazard ratio.

^a^ Surgical site infection was the main event; relaparotomy and death were competing risks; and treatment group, level of contamination, and BMI class were covariates.

^b^ Compared to BMI <18.5.

Secondary end point analysis (surgical site infection depth, wound complications, length of hospital stay, 30-day mortality or morbidity, 30-day reoperation rate, and incidence of adverse events and serious adverse events) revealed no significant differences between polyhexanide and no irrigation or saline (Table 2). Most surgical site infections were grade I (46 of 81 [56.8%]), followed by grades III (21 of 81 [25.9%]) and II (14 of 81 [17.3%]). Furthermore, 73 of 689 patients overall (10.6%) required relaparotomy: 27 of 292 (9.2%) in the polyhexanide group, 31 of 295 (10.5%) in the saline group, and 15 of 102 (14.7%) in the no irrigation group. Ten of 689 patients (1.5%) died within 30 days of surgery, all of them without having had a surgical site infection: 2 of 292 (0.7%) in the polyhexanide group, 6 of 295 (2.0%) in the saline group, and 2 of 102 (2.0%) in the no irrigation group. Another patient died after day 30 before completing the last study visit. Noninfectious wound complications were recorded in 125 of 689 patients overall (18.8%), evenly distributed among the study arms (Table 2).

Prespecified subgroup analyses were conducted using the Fine-Gray subdistributional hazard model with surgical site infection grade I or II as the main event; surgical site infection grade III, relaparotomy, and death as competing risks; and treatment group, level of contamination, BMI, and the parameters of interest as covariates revealed no impact on incidence of surgical site infection grade I or II, except with colorectal procedures (HR, 2.59; 95% CI, 1.39-4.84; *P* = .003) (Table 4). A bundle outcome incorporating the application of wound edge protectors, glove change, and polyhexanide irrigation did not contribute to surgical site infection reduction either in the whole study population (HR, 1.14; 95% CI, 0.49-2.68; *P* = .76) or in colorectal procedures only.

**Table 4.** Subdistributional Fine-Gray Hazard Model^a^

Variable	HR (95% CI)	*P* value
Treatment group		
No irrigation vs polyhexanide	1.39 (0.63-3.08)	.41
Saline vs polyhexanide	1.31 (0.71-2.43)	.39
Level of contamination		
III or IV vs I or II	0.63 (0.17-2.36)	.50
BMI^b^		
18.5-<25	2.04 (0.28-15.19)	.49
25-<30	1.58 (0.20-12.22)	.66
≥30	1.06 (0.13-8.89)	.96
Colorectal surgery		
Yes vs no	2.59 (1.39-4.84)	.003

Abbreviations: BMI, body mass index (calculated as weight in kilograms divided by height in meters squared); HR, hazard ratio.

^a^ Incisional surgical site infection was the main event; organ or space surgical site infection, relaparotomy, and death were competing risks; and treatment group, BMI class, and colorectal surgery were covariates.

^b^ Compared to BMI <18.5.

The frequency, severity, causal relationship, and type of serious adverse event for the 3 treatment groups are shown in eTable 3 in Supplement 2. The median (range) number of adverse events per patient was 2 (0-20) and the median (range) number of serious adverse events per patient was 0 (0-8). Severe adverse events were detected mostly in the no irrigation-group, while life-threatening serious adverse events appeared most frequently in the polyhexanide group. Serious adverse event–related mortality was lowest in the polyhexanide group (2 of 292 [4.5%]). Mild and moderate serious adverse events were evenly distributed between the groups. One serious adverse event in the saline group was deemed related. The distribution and Clavien-Dindo classification of all surgical complications is presented in eTable 4 in Supplement 2.

## Discussion

In this randomized clinical trial, intraoperative wound irrigation with polyhexanide solution did not reduce surgical site infection incidence after laparotomy for clean-contaminated surgical procedures compared to saline or no irrigation. No differences were found among the treatment groups regarding the secondary end points. This result was unexpected, as previously available evidence revealed an anticipated 75% risk reduction. An explanation is provided by the fact that 92.8% of the included procedures were intraoperatively graded as level of contamination II, which was not anticipated when the clinical trial was designed. In line with this observation, a recent single-center randomized clinical trial^23^ found no surgical site infection reduction after aqueous povidone-iodine wound irrigation in abdominal level of contamination II procedures either. The initial pragmatic trial design aimed to include level of contamination II to IV procedures without controlling the number of patients per contamination class. However, the proportion of level of contamination III or IV procedures in the present trial was surprisingly low, which resulted in a lower than expected overall surgical site infection rate of 11.8%. On the one hand, this is attributable to the difficulty of including emergency patients into clinical trials during the night or proceeding to surgery too quickly to obtain informed consent beforehand. On the other hand, the low surgical site infection rate reflects the increased use of minimally invasive techniques especially for colorectal procedures, whereas hepatobiliary-pancreatic procedures still more commonly involve laparotomy. Furthermore, the low overall surgical site infection rate of 11.8% may reflect the contribution of preventive measures implemented in recent years (glove changes or application of wound edge protectors) in open abdominal procedures. However, the present trial did not show an additional beneficial effect of glove changes or wound edge protector application as beneficial factors in level of contamination II procedures. This observation is in accordance with the Covering of the Abdominal Wall in Laparotomies: Differences in Surgical Site Infections Between an Approved Abdominal 3M Steri-Drape Wound Edge Protector and Standard Woven Swabs at Technische Universität München (BAFO) trial,^6^ which demonstrated that surgical site infection reduction using wound edge protectors was achieved only in level of contamination III or colorectal procedures.

In contrast, the RECIPE trial^8^ evaluating intraoperative wound irrigation with polyhexanide showed a significant surgical site infection reduction in abdominal surgery. One-third of participants experienced inflammatory bowel disease and received immunosuppressive therapy at the time of surgery, which explains the high overall surgical site infection rate of 28%. The proportion of included colorectal and level of contamination III procedures was higher than in the IOWISI trial. The population of the IOWISI trial consisted of 80% oncological patients undergoing major surgery. Both the prevalence of noninfectious wound complications and the serious adverse event rates were equally distributed among the groups, which demonstrates the safety of polyhexanide in this indication. It also refutes the hypothesis that intraoperative wound irrigation itself may be harmful by washing away inflammatory defense cells^13^ or washes bacteria from the skin into the wound.

### Limitations

The following other limitations must be considered. First, surgeons performing the intervention could not be blinded, as there was no wound irrigation in the control group. Second, polyhexanide is currently not approved for intraperitoneal irrigation and thus the laparotomy wound could only be decontaminated in the epifascial layers. To investigate if intraperitoneal antiseptic lavage has a stronger effect on surgical site infection rates than lavage of the wound only, a successive study investigating hypochlorite solution was initiated in September 2022.^24^ In addition, polyhexanide requires a tissue contact time of at least 10 minutes to take full effect. In the IOWISI trial it was mandatory to keep the wound moistened with the irrigation solution after removing the excess by suction. It is conceivable that adherence to the application time at the end of lengthy surgical procedures may not have been adequate and thus full effectiveness may not have been achieved. However, a pilot trial^25^ compared long and short exposure of polyhexanide solution in laparotomy wounds and found no significant differences.

Third and most importantly, the number of dropouts was twice as high as anticipated at the planning stage of the trial, mostly because emergency relaparotomy rates for postoperative complications after trial inclusion were higher than expected. Moreover, the management of postoperative complications, such as anastomotic leakage, intraabdominal abscess, or postoperative intraabdominal hemorrhage varied among the participating centers (relaparotomy vs noninvasive treatments). To maintain the desired statistical power, the sample size calculation and statistical analysis had to be adjusted during the trial. Additionally, the generalizability of the trial results is limited due to the fact that most of the patients who were prescreened were scheduled for minimally invasive surgery and thus did not meet the inclusion criteria. Only around 11% of all prescreened patients were scheduled for primarily open abdominal surgical procedures. However, open abdominal surgery will remain relevant because there still are technical and financial limitations of minimally invasive procedures, especially in smaller community or rural hospitals and in low- to middle-income health care systems where surgical site infection prevention is crucial.

## Conclusions

To our knowledge, the IOWISI trial is the first large multicenter randomized clinical trial to investigate intraoperative polyhexanide irrigation and is characterized by high internal validity, as the surgical irrigation technique and blinded outcome assessment were consistently standardized. The multicenter approach ensures high external validity because all types of visceral procedures were included, resulting in a representative study population. Therefore, the IOWISI trial is likely to contribute substantially to any subsequent clinical recommendations in abdominal surgery and other surgical fields. In conclusion, on the basis of the present results, intraoperative wound irrigation with polyhexanide cannot be recommended as standard clinical practice in open clean-contaminated (level of contamination II) visceral surgical procedures. More clinical trials are warranted to evaluate the potential benefit of polyhexanide intraoperative wound irrigation in colorectal, contaminated (level of contamination III) and septic (level of contamination IV) procedures.

## Notes

Supplement 1. Trial protocol

Supplement 2. eAppendix 1. CDC classification of intraoperative Level of Contamination (LOC) eAppendix. 2. CDC classification of Surgical Site Infection (SSI) eTable 1. Sensitivity analysis of the primary endpoint with the subdistributional hazard model of Fine and Gray with SSI as main event, re-laparotomy and death as competing risks, treatment group, and several patient characteristics as covariates eTable 2. Analysis of the primary endpoint with the subdistributional hazard model of Fine and Gray with SSI as main event and relaparotomy and death as competing risks, and treatment group, level of contamination, BMI class, cardiovascular, and pulmonary comorbidities as covariates eTable 3. Frequency, severity, causal relationship, and types of serious adverse events for the wound irrigation groups PHX, saline, and no irrigation eTable 4. Clavien‒Dindo classification of surgical complications (including SSI I-III) in the IOWISI trial by study group

Supplement 3. IOWISI study group members

Supplement 4. Data sharing statement
