# Supplementary material for: Intraoperative Wound Irrigation for the Prevention of Surgical Site Infection After Laparotomy: A Randomized Clinical Trial by CHIR-Net
Source: JAMA Surg. 2024 Feb 21;159(5):484–92. doi: 10.1001/jamasurg.2023.7985 (PMC10882507; doi:10.1001/jamasurg.2023.7985)
Supplement: Supplement 1. — Trial protocol [file jamasurg-e237985-s001.pdf]

Supplement to Manuscript Submission JAMA Surgery

“Intraoperative wound irrigation for the prevention of surgical site infection after laparotomy

The multicenter, double-blind, randomized controlled IOWISI trial (DRKS00012251) of the Study Centre of the German Surgical Society (SDGC CHIR-Net)”

This supplement contains the following items:

1. Clinical study protocol (CSP)

1.1 CSP original version 2.0 (06.06.2017) Page 1-41

1.2 CSP final version 3.0 (02.03.2021) Page 42-82

1.3 CSP summary of changes Page 83

2. Statistical analysis plan (SAP)

2.1 SAP original version 1.0 (28.04.2021) Page 84-95

2.2 SAP final version 1.1 (08.11.2022) Page 96-107

2.3 SAP summary of changes Page 108

1. Study Protocol

1.1 CSP original version 2.0 (06.06.2017)

TECHNISCHE UNIVERSITÄT MÜNCHEN KLINIKUM RECHTS DER ISAR

STUDY PROTOCOL

IOWISI

Intraoperative wound irrigation to prevent surgical site infection after laparotomy

Sponsor:

Technische Universität München (TUM)
School of Medicine
Represented by the Dean
Ismaninger Str. 22
81675 München, Germany

Coordinating Investigator (LKP):

PD Dr. med. Daniel Reim
Klinik und Poliklinik für Chirurgie
Klinikum rechts der Isar TUM
Ismaninger Str. 22
81675 München, Germany

EudraCT Number: 2017-000152-26 **Study Code:** IOW-1755-REI-0540-I

DRKS Number: DRKS00012251

Version: Final 2.0 **Date** 06.06.2017

! Confidential !
This document is confidential and should serve as a source of information for Investigators and other personnel involved in this clinical study, consultants and ethics committees and regulatory authorities. The contents of this document shall only be disclosed to others in agreement with the coordinating investigator and/or sponsor.

Responsibility

**Protocol development**	**Sponsor**	**Technische Universität München**<br>**School of Medicine**<br>**Represented by the Dean**<br>Ismaninger Str. 22<br>81675 München<br>Tel.: 089 4140-4020<br>Fax: 089-4140-4935<br>E-Mail: dekanat.medizin@tum.de
	**Coordinating investigator (LKP)**	**PD Dr. med Daniel Reim**<br>**Klinik und Poliklinik für Chirurgie**<br>Klinikum rechts der Isar der<br>Technischen Universität München (TUM)<br>Ismaninger Str. 22<br>81675 München<br>Tel.: 089 4140-5019<br>Email: daniel.reim@tum.de
	**Clinical project management**	**Dr. med. Tara Mueller**<br>**Klinik und Poliklinik für Chirurgie**<br>Klinikum rechts der Isar der<br>Technischen Universität München (TUM)<br>Ismaninger Str. 22<br>81675 München<br>Tel.: 089 4140-2145<br>Email: tara.mueller@tum.de
	**Trial statistican**	**Dr. rer. nat. Victoria Kehl**<br>**Institut für Medizinische Statistik und Epidemiologie (IMSE)**<br>Technische Universität München (TUM)<br>Ismaninger Str. 22<br>81675 München<br>Tel: 089 4140-4355<br>Fax: 089 4140-4850<br>Email: victoria.kehl@tum.de
	**Project management**<br>**Monitoring**<br>**Data management**<br>**Safety management**	**Münchner Studienzentrum (MSZ)**<br>Ismaninger Str. 22<br>81675 München<br>Tel: 089 4140-6321<br>Fax: 089 4140-6322<br>Email: Beate.schossow@mri.tum.de

1. DECLARATION OF INVESTIGATOR

I have read the trial protocol and I confirm that it contains all information to accordingly conduct the clinical trial. I pledge the clinical trial will be conducted at my trial center according to the protocol.

The first patient will be enrolled only after all ethical and regulatory requirements are fulfilled. I pledge that written informed consent for trial participation will be obtained from all patients.

I know the requirements for accurate notification of serious adverse events and I pledge to document and notify such events as described in the protocol.

I pledge to retain all trial-related documents and source data as described. All necessary documents will be provided before trial start. I agree that these documents will be submitted to the responsible regulatory authorities and ethics committees.

2. SYNOPSIS

**Sponsor**	Technische Universität München, School of Medicine
**Name of the trial**	Intraoperative wound irrigation to prevent surgical site infection after laparotomy<br>- IOWISI
**Trial design**	Prospective, randomized, controlled, observer and patient-blinded, multicenter, surgical trial according to German drug law (AMG) phase IIIb, with three parallel comparison groups
**Objectives**	To investigate whether the use of intraoperative, epifascial wound irrigation with polyhexanide (PHX) solution can reduce surgical site infections after laparotomy for visceral surgery compared to saline irrigation or no irrigation.
**Interventions**	<u>Experimental intervention/index test:</u><br>• Intervention 1: Irrigation of the subcutaneous tissue after closure of the abdominal fascia with 1000ml PHX solution (0.04%)<br>• Intervention 2: Irrigation of the subcutaneous tissue after closure of the abdominal fascia with 1000ml saline solution (NaCl 0.9%)<br><u>Control intervention/reference test:</u><br>No epifascial wound irrigation<br><u>Follow-up per patient</u>:<br>Postoperative day 30 (+6 at the latest)<br><u>Duration of intervention per patient</u>:<br>One intraoperative application<br><u>Experimental and/or control off-label or on-label in Germany</u>:<br>All interventions are on-label in Germany
**Key inclusion and exclusion criteria**	<u>Key inclusion criteria</u>:<br>• Clean-contaminated, contaminated or dirty surgery (class II-IV) according to Centre for Disease Control (CDC) classification;<br>• Abdominal surgery by midline or transverse laparotomy; elective and emergency procedures;<br>• Age ≥ 18 years;<br>• American Society of Anesthesiologists (ASA) score ≤ 3;<br>• Ability to understand the nature and extent of the trial and to give written informed consent;<br><u>Key exclusion criteria</u>:<br>• Pregnancy or breast feeding;<br>• Known hypersensitivity/allergy to PHX;<br>• Inability to give/understand informed consent;<br>• Critical medical condition of emergency patients, precluding informed consent or sufficient time to reflect on the decision to participate in the trial;<br>• ASA >3;<br>• Inability to attend follow-up visits;<br>• Clean procedures according to the CDC classification or surgery without opening of the abdominal cavity;<br>• Laparoscopic surgery;<br>• Revision-surgery (previous abdominal surgery within the last 30 days);<br>• Planned re-laparotomy within 30 days;<br>• Severe immunosuppression;<br>• Concurrent abdominal wall infections;<br>• Pre-operative systemic antibiotic therapy within 5 days prior to surgery (except emergency pre-operative antibiotic treatment due to septic peritonitis after admission to the hospital);<br>• Participation in another clinical trial that interferes with the primary or secondary outcomes of this trial.
**Outcomes**	<u>Primary efficacy endpoint</u>:<br>SSI according to CDC criteria within 30 days postoperatively<br><br><u>Key secondary endpoint(s)</u>:<br>• Non-infectious wound complications (*e.g.* seroma, hematoma, delayed healing) within 30 days postoperatively

	• Duration of hospital stay<br>• Mortality and morbidity within 30 days postoperatively<br>• Incidence of reoperation within 30 days postoperatively<br>• Incidence of AE/SAE within 30 days postoperatively<br>Pre-specified subgroup analysis by category of SSI (superficial, deep, organ space), NNSI risk score, ASA score, BMI, age, diabetes, smoking, alcohol consumption, history of SSI, history of radio-/chemotherapy, pre-operative hospital stay >2d, administration and timing of antibiotic prophylaxis, type and duration of surgery, intraoperative use of wound-edge protectors and changing of gloves, presence of an enterostomy.<br><br><u>Safety:</u><br>Adverse events (AE) and serious adverse events (SAE) are documented for all groups. Surgical complications will be additionally evaluated according to the Clavien-Dindo classification
**Study registry**	German CTR (DRKS): DRKS00012251 / EudraCT: 2017-000152-26
**Statistical analysis**	<u>Efficacy</u>: The incidence of SSI within 30 days after surgery will be compared between three study groups in two ways:<br>Test 1: PHX irrigation *vs*. saline irrigation<br>Test 2: PHX irrigation *vs.* no intervention<br><u>Description of the primary efficacy analysis and population</u>:<br>The incidence of SSI within 30 days of surgery will be compared in test 1 and test 2 using the Fisher Exact test. Both tests will be performed on the ITT set, consisting of all patients included in the study in the treatment arm they were randomized to. First analysis will be based on all patients with complete follow-up. For sensitivity, multiple imputations for missing primary endpoint data will be used. The tests will be performed two-sided and with a global significance level of 5%. Using the Bonferroni-Holm adjustment, the local significance level for test 1 will be 2.5% and for Test 2 it will be 5%.<br><u>Safety</u>: The assessment of safety will be based on the frequency of AE/SAE other than SSI within the safety population (according to CTCAE Version 4.03), consisting of all patients randomized into the study.<br><u>Secondary endpoint(s)</u>: Secondary endpoints will be analyzed on the ITT set using appropriate descriptive statistics. Any explorative statistical testing will be performed two-sided using a significance level of 5%.
**Sample size**	<u>To be assessed for eligibility (n):</u> approximately 1500<br><u>To be assigned to the trial (n)</u>: 540<br><u>To be analyzed (n)</u>: 540<br>The sample size was calculated assuming 30-day SSI rates of 2.2% in the PHX group, 8.7% in the saline group, and 16.2% in the control group. If 230 patients are recruited in the PHX group, 230 patients in the saline group and 80 patients in the no irrigation group (a total of 540 patients), the two-sided Fisher exact test with a global significance level of 5% will have a power of 94% for test 1 (α=2.5%) and a power of 85% for test 2 (α=5%) to detect differences between the treatment groups.
**Trial duration subject**	<u>Intervention: Single intraoperative intervention</u><br><u>Follow-up: max. 36 days</u>
**Trial duration project**	<u>First patient in to last patient out (months)</u>: 28<br><u>Recruitment period (months)</u>: 27<br><u>Duration of the entire trial (months)</u>: 42
**Participating centers**	Planned: n= 10
**Financing**	Deutsche Forschungsgemeinschaft (DFG) grant number: MU 3928/1-1

3. ABBREVIATIONS

AE	Adverse Event
ALT/ALAT	Alanine Aminotransferase
AMG	Arzneimittelgesetz
aPTT	Activated partial Thromboplastin time
ASA	American Society of Anesthesiologists
AST/ASAT	Aspartate Aminotransferase
BfArM	Bundesinstitut für Arzneimittel und Medizinprodukte
BMI	Body-Mass Index
CDC	Centre for Disease Control and Prevention
CI	Confidence Interval
Cr	Creatinine
CTCAE	Common Terminology Criteria for Adverse Events
DFG	Deutsche Forschungsgemeinschaft
DRKS	Deutsches Register Klinischer Studien
DSUR	Development Safety Update Report
eCRF	electronic Case Report Form
EDTA	Ethylene-diamineteraacetic acid
GCP	Good Clinical Practice
Glu	Glucose
ICF	Informed consent form
ICH	International Conference on Harmonization
ICMJE	International Committee of Medical Journal Editors
IMP	Investigational Medicinal Product
IMSE	Institut für Medizinische Statistik und Epidemiologie
INR	International normalized ratio
IOWI	Intraoperative wound irrigation
ISF	Investigator site file
ITT	Intention-To-Treat
K	Potassium
MeSH	Medical Subject Heading
MRI	Klinikum München rechts der Isar
MSZ	Münchner Studienzentrum
Na	Sodium
NaCl	Sodium chloride
NICE	National Institute for Health and Clinical Excellence
NNIS	National Nosocomial Infections Surveillance
PHX	Polyhexanide
PRISMA	Preferred Reporting Items for Systematic Reviews and Meta-Analyses
PT	Prothrombin time
PVP	Polyvinylpyrrolidone, Povidone
RCT	Randomized Controlled Trial
RDE	Remote Data Entry
SAE	Serious Adverse Event
SAR	Serious Adverse Reaction
SAS	Statistical analysis system
SGOT	Serum glutamic oxaloacetic transaminase
SGPT	Serum glutamic pyruvic transaminase
SMB	Safety Monitoring Board
SmPC	Summary of product characteristics
SOP	Standard operating procedure
SSI	Surgical site infection
SUSAR	Suspected Unexpected Serious Adverse events
TUM	Technical University of Munich
WHO	World Health Organization

4. INTRODUCTION

4.1 The medical problem

Postoperative surgical site infection (SSI) represents the third most common hospital infection. According to the CDC's classification [1], SSI can be subdivided into infections of the subcutaneous tissue (superficial SSI), deep soft tissues such as fascial and muscle layers (deep SSI) and infections of organs or spaces (organ/space SSI) that occur within 30 days after surgery (attachment 1). In abdominal surgery, SSI rates are especially high. Recent high-level randomized controlled trials (RCTs) with standardized SSI definitions found rates between 14.5% (BaFO trial) [2], 15.4% (PROUD trial) [3] and 25.0% (ROSSINI trial) [4] following laparotomy. Therefore, measures to prevent SSI in this field are urgently needed. Prophylactic intraoperative wound irrigation (IOWI) of the subcutaneous and deep soft tissue before skin closure with saline or antiseptic solutions hypothetically represents an easy and economical option to reduce SSI rates and is already frequently used in clinical practice, even though there are currently no definite recommendations on this practice [5]. The latest official guideline for the prevention of SSI by the World Health Organization (WHO) published in 2016, states that IOWI with saline is not efficient, but IOWI with diluted Polyvinylpyrrolidone (PVP)-iodine solutions has a potential benefit in preventing SSI, however, due to the low level of underlying evidence these recommendations are conditional and not limited to abdominal surgery [6]. In contrast, the clinical guidelines of the British National Institute for Health and Clinical Excellence (NICE) from 2008 state that IOWI's efficacy is unproven and its use should be avoided at all. However, this recommendation too, is based on a small number of unstandardized RCTs evaluating different types of surgery and irrigation solutions [7]. Antiseptic PHX-based solutions are approved for intraoperative soft-tissue wound irrigation in surgery, and have been shown to be tissue tolerable and even promote wound healing. To our knowledge prophylactic PHX wound irrigation has not yet been evaluated in RCTs in abdominal, visceral surgery [8, 9].

4.2 Evidence

Even though the literature concerning prevention of SSI is substantial, high-level evidence to guide decisions on the use of IOWI with saline or antiseptics remains scarce. Clinical trials investigating the efficacy of IOWI have been conducted mainly in the 1980-90's and their results are inconclusive and heterogeneous patient inclusion and outcome criteria were used. A few authors conducted systematic reviews and meta-analyses investigating specific irrigation solutions such as PVP-iodine or antibiotic solutions [10-13]. However, none of these reviews resulted in a definite conclusion, although they all observed a positive trend in the reduction of SSI rates through IOWI. Furthermore, more recent clinical trials have been conducted in the meantime. Therefore, we performed a large-scale meta-analysis in accordance with the Cochrane guidelines of the existing evidence on IOWI with saline, PVP-iodine or antibiotic

irrigation solutions. Pubmed/MEDLINE, EMBASE, and the Cochrane Central Register of Controlled Trials (CENTRAL) were searched in May 2013. The following search terms were used in various combinations: prevention of surgical site infection, abdominal surgery, surgical wound infection/prevention and control [MeSH Terms], wound irrigation, wound lavage, incisional surgical site infection, intra operative irrigation, intra operative lavage, antibiotic irrigation, antibiotic irrigation solutions, iodine irrigation, povidone iodine irrigation, saline irrigation, and topical anti-infective agents [MeSH Terms]. The abstract and title search was limited to clinical trials published in English or German between January 1, 1970 and May 1, 2013. In addition, all articles within the reference list of retrieved studies and reviews were hand-searched. The search was performed by two independent reviewers and followed the published protocol corresponding to the PRISMA statement and the Cochrane Handbook of systematic reviews of interventions. Prospective RCTs investigating the primary outcome of postoperative SSI after IOWI of the surgical incision after closure of the fascia or peritoneum and before skin closure were eligible for inclusion. Eligible irrigation solutions were saline, PVP-iodine, or topical antibiotics in different forms and concentrations (dry powder sprays or wound powder were also acceptable), irrespective of the closure and irrigation technique. Acceptable comparators were 'no irrigation' or irrigation with saline. All types of open abdominal surgeries were eligible, including visceral, gynecological, urological, or vascular procedures irrespective of the urgency of operation (elective or emergency). All trials reporting clinical SSI were included irrespective of the SSI definition used. Trials in which only one of the compared treatment arms received systemic prophylactic antibiotics were excluded, as this would have caused substantial bias. Methodological quality of individual clinical trials was assessed by examination of the allocation sequence, allocation concealment and double blinding using the Cochrane tool for assessing the risk of bias [21]. The risk of bias was graded as low, unclear, or high. In addition, the risk of publication bias was investigated by means of a funnel plot. Due to the naturally expected heterogeneity in performance of surgical procedures between different types of surgery, grade of contamination, and hence trials, random effect models with Mantel-Haenszel weights were used to estimate the average treatment effect and a corresponding 95 % CI. Forest plots were shown to illustrate treatment effects estimated for each trial and the estimated average treatment effect for all investigated subgroups. A two-sided level of significance of less than 5.0 % was considered for all tests. The results of this analysis show a risk reduction of 46 % in the treatment group (IOWI with any irrigation solution). Incidence of SSI was 9% in the irrigation group compared to 16% in the untreated group [14]. However, the majority of included trials have been published from 1970 to 1990, and the quality assessment revealed that most of them were at a high risk of bias, mainly because of insufficient data reporting and methodological flaws. Methods of sequence generation, allocation concealment, and blinding were often inadequate or not reported. In addition, interventions, follow-up times, and definitions

of SSI varied widely between studies, which might explain the large variance in overall SSI rates between 3.0 and 58.2%. Most studies used a non-standardized definition of SSI. The current internationally accepted CDC definition was not published until 1999. The funnel plot showed an asymmetry, which indicates a possible publication bias, as all included trials with a high standard error for the log odds ratio show a large benefit for the experimental group. Furthermore, PVP-I and antibiotic solutions are currently not recommended for this indication due to potential adverse side effects, tissue toxicity and the increased development of antimicrobial resistances. The only standardized RCT comparing IOWI with saline irrigation *vs.* no irrigation after open appendectomies was published in 2000 and found a reduction of SSI from 25% to 8.7% in the saline group [15]. Recently, PHX-based antiseptic solutions are successfully and widely used in orthopedic and trauma surgery. Wound irrigation with PHX showed a reduction of the SSI rate of almost 75% compared to Ringers solution in traumatic dirty contaminated soft tissue wounds [16].

4.3 The need for a trial

SSIs contribute significantly to postoperative morbidity and mortality. In Germany approximately 128,000 SSIs are reported annually [17]. Studies have shown an increase of 6-24 days in the mean length of hospital stay if SSI occurs [18]. In addition to the risk and discomfort for the patient, SSIs dramatically increase treatment costs and indirect costs such as loss of workforce or insurance payments. In Germany, postoperative SSIs account for approximately 1 million extra days of hospitalization and additional costs of around € 3 billion per year [19, 20]. Clinical guidelines and clinical practice vary largely in terms of the use of IOWI to reduce the incidence of SSI [5]. The aim of this prospective, multicenter, randomized clinical trial is to show the reduction of SSI rates by IOWI with PHX compared to saline or no irrigation. Individual patients participating in this trial have the opportunity of directly benefitting of the anticipated positive effect of PHX and/or saline irrigation, whilst no negative effects are to be expected. The results of the trial will provide evidence for definite clinical recommendations that would change current clinical guidelines and practice. A commercial interest is not expected as PHX solutions are widely available and several companies offer this product in their portfolio. The trial further does not request a certain product in order to avoid compliance conflicts, but encourages collaborators to use the available product in their respective study sites.

4.4 Summary and aims of the study

SSI is one of the most common complications following abdominal visceral surgery (14-25%) [2-4, 21] and dramatically increases length of hospital stay and costs. Hypothetically, IOWI before skin closure with saline or antiseptics might be a potential pragmatic option to reduce SSI rates. Currently, there are no official recommendations on its use and clinical practice varies largely.

Solutions containing the antiseptic agent PHX are approved for IOWI, and were shown to promote wound healing [8, 9], but have not been evaluated in RCTs in abdominal visceral surgery. Therefore, we designed a multicenter, randomized, observer-blinded clinical trial evaluating the efficacy of IOWI with PHX solution or saline before skin closure after laparotomy. Based on a meta-analysis on IOWI with various solutions, a sample-size of 540 patients was calculated for a 3-armed study design (PHX- *vs.* saline irrigation *vs.* no irrigation). The trial shall be conducted in 10 centers within the German surgical trial network CHIR-*Net*. All patients undergoing visceral surgery by laparotomy within the recruitment period of 27 months will be screened for the trial. The primary endpoint is the incidence of SSI 30 days postoperatively, according to the CDC definition (attachment 1). The results of the trial will provide evidence for definite clinical recommendations regarding the use of IOWI and influence current guidelines and provide all participating patients the opportunity of an improved treatment.

5. OUTCOME MEASURES

5.1 Rationale of outcome measures

The primary efficacy endpoint of this trial is SSI within 30 days postoperatively, according to the internationally accepted and recommended SSI definition by the CDC [1]. This endpoint has been used in previous trials and assures comparability of the results [2-4, 21]. This endpoint is further considered to be of clinical relevance as SSI increases morbidity and mortality of individual patients, direct and indirect costs and prolongs hospital stay as outlined before. The secondary endpoint of non-infectious wound complications was chosen to evaluate, if PHX irrigation has an additional positive effect on wound healing. Furthermore, secondary endpoints are morbidity and mortality within 30 days postoperatively. For safety analyses and the duration of hospital stay to evaluate the potential economical benefit.

5.2 Determination of primary and secondary measures

The primary efficacy endpoint measure of the trial is the incidence of SSI within 30 days after surgery diagnosed. Furthermore, in case of SSI, the depth of infection will be classified into one of three categories according to CDC definition (superficial, deep, organ-space, see attachment 1). In addition, the following outcome measures have been defined as secondary endpoint measures and will be determined by the unit given in parentheses: a) Duration of hospital stay (in days); b) 30-days rate of reoperation in both groups (%); c) 30-days rate of non-infectious wound complications in both groups (in %); d) 30-days rate of postoperative AE/SAE in both groups (%); e) 30-days mortality in both groups (%); (f) 30-days morbidity in both groups (%). All AE/SAEs that are surgical complications will be additionally classified according to the Clavien Dindo classification of surgical complications (attachment 2) [22].

6. FINANCING

The clinical trial is financed by a grant from the German Research Society (Deutsche Forschungsgemeinschaft; DFG), grant number: MU 3928/1-1. No co-financing by industry or other third parties applies. There is no conflict of interest for the management of the study. All participating trial sites have officially declared no conflict of interest within the eligibility evaluation of the MSZ. A commercial interest does not apply as PHX solutions are widely available and several companies offer this product in their portfolio. The trial further does not request a certain product in order to avoid compliance conflicts, but encourages collaborators to use the available product in their respective study sites.

7. RISK / BENEFIT ANALYSIS

No additional risks for study patients are anticipated, since IOWI represents a clinically established standard method. PHX 0.04% irrigation solution is approved for surgical wound irrigation of soft tissue wounds. The study will be planned, conducted and analysed according to all relevant national and international rules and regulations according to AMG [23], ICH-GCP E6 [24], and the Declaration of Helsinki, 2008 (see 27.). No specific risks are expected because IOWI is locally applied and neither application of PHX or saline will have systemic effects on the participants. Safety of PHX solutions has been demonstrated before in the marketing studies. Adverse effects may only be expected in the improbable event of accidental contamination of the respective irrigation solutions or in case of unknown hypersensitivity to PHX. The potential benefits of reduced SSIs outweigh the mentioned negligible adverse effects of PHX and saline. The subjects´ safety is ensured by regular study visits, enforcing GCP-guidelines. A subject-insurance for all trial participants is mandatory according to AMG. The informed consent process adheres to GCP-guidelines, which maximize patients´ safety and guarantee confidentiality.

8. TRIAL IMPLEMENTATION

8.1 General study design

This study is a prospective, randomized, controlled, observer and patient-blinded, multicenter, surgical trial with three parallel comparison groups. Pre-screening of potential patients (evaluation of inclusion and exclusion criteria) is possible up to 14 days prior to the planned procedure. Patients can be included in the trial if inclusion and exclusion criteria apply and

written informed consent has been provided. In case of emergency procedures inclusion is possible on the same day as the procedure, if the patient is able to understand and provide written informed consent and has had a reasonable amount of time to think about the decision (see 12.3). Included patients are randomized to no epifascial wound irrigation, epifascial wound irrigation with saline 0.9% or epifascial wound irrigation with PHX 0.04% solution. Screened but excluded patients will be documented in a screening log.

8.2 Trial duration

The estimated overall length of the study is 42 months, which assembles as follows:

I. Trial preparation: ~ 6 months

II. Execution of study: First patient in to last patient out: ~ 28 months

1. Begin of study: 3rd quarter, 2017
2. End of study: 4th quarter, 2019 (Completion of the last visit for the last patient represents the end of study)
3. Recruitment period: ~ 27 months
4. Duration of treatment per patient:
   a) Group with intervention 1: Surgery according to institutional standard, followed by one-time wound irrigation with PHX 0.04% solution.
   b) Group with intervention 2: Surgery according to institutional standard, followed by one-time wound irrigation with saline 0.9% solution.
   c) Control group: Surgery according to institutional standard, followed by no wound irrigation.
5. Duration of follow-up per patient: 30 days (+5 days at the latest)

For all three groups, documentation of the primary and secondary endpoints up to postoperative day 30 is warranted.

III. Analysis, publication ~ 8 months

Graph 1: IOWISI intervention scheme / trial flow

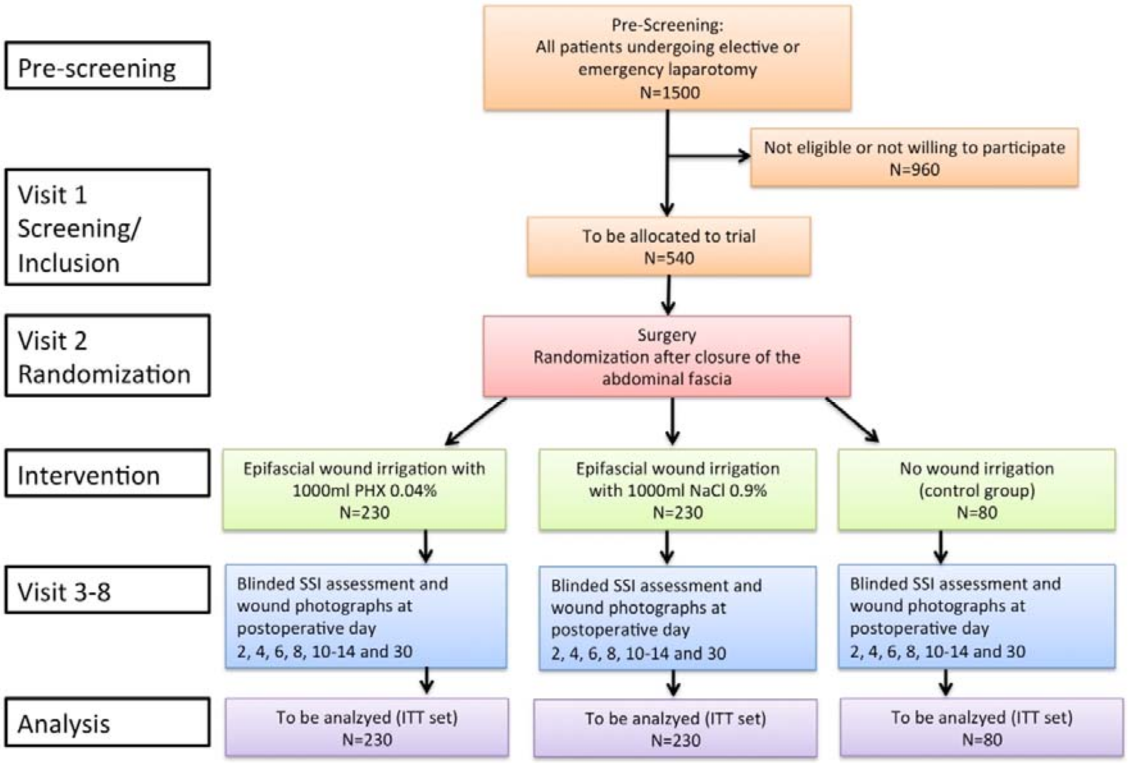

Graph 2: IOWISI study visits (according to SPIRIT statement 2013 [25])

	**STUDY PERIOD**							
	**INCLU.**	**RAND.**	**POST-ALLOCATION**					**CLOSE-OUT**
**STUDY VISIT**	**1**	**2**	**3**	**4**	**5**	**6**	**7**	**8**
**TIMEPOINT**	- 1-3 days*	Surgery (day 0)	day 2	day 4	day 6	day 8	day 10-14	day 30§
**INCLUSION**								
Informed consent	X							
Inclusion and exclusion criteria	X							
**RANDOMIZATION**		X						
**INTERVENTIONS**								
Intervention 1(IOWI with 1000ml PHX 0.04%)		X						
Intervention 2(IOWI with 1000ml NaCl 0.9%)		X						
Control group (no IOWI)		X						
**ASSESSMENTS**								
Demographical data	X							
Medical history	X							
Concurrent medication	X							
Physical examination	X							
NNSI Risk score	X							
Pregnancy test**	X**							
Blood sample***	X			X****				
Type of operation		X						
Duration of operation		X						
Level of contamination		X						
Type and length of incision		X						
Wound closure technique and suture material		X						
Creation of an enterostomy		X						
Administration and timing of antibiotic prophylaxis		X						
Intraoperative use of wound edge protectors		X						
Changing of gloves during operation		X						
Postoperative medication with effect on wound healing			X	X	X	X	X	X
Documentation of SSI			X	X	X	X	X	X
Documentation of other wound complications			X	X	X	X	X	X
Wound swab for microbiology+			X+	X+	X+	X+	X+	X+
Photograph of the wound			X	X	X	X	X	X
Documentation of re-operation			X	X	X	X	X	X
Documentation of AE/SAE		X	X	X	X	X	X	X
Duration of hospital stay								X

* In case of emergency surgery enrolment is possible on the same day as the procedure
**For women of child-bearing potential only (serum or urine)
***Includes hemoglobin, hematocrit, platelets and white blood cell count, Na, K, Cr, Glu (non-fasting), AST/ASAT (SGOT), ALT/ALAT (SGPT), Bilirubin, Uric acid, Prothrombin time (PT), activated partial thromboplastin time (aPTT), international normalized ratio (INR) according to local in-house standards
****Between post-OP day 4-8 (visit 4-6)
+In case of SSI a swab will be taken from the wound or wound secretion for microbiological differentiation and testing of resistance to antibiotics according to local in-house standards

§ Visit window +6 days. If the patient is unable to attend visit 8 due to postoperative treatment in a rehabilitation facility or other medical reasons, a standardized protocol for evaluation and documentation of the wound will be sent to and filled out by the treating physician.

9. JUSTIFICATION OF DESIGN ASPECTS

9.1 Study design

This trial is a prospective, randomized, controlled, observer and patient-blinded, multicenter, surgical trial according to German drug law (AMG) phase IIIb with three parallel comparison groups. Reduction of SSI (according to CDC criteria) by IOWI after abdominal surgery is postulated. The IOWISI trial will be conducted in approximately 10 surgical departments (university and community hospitals), all of which are members of the trial network (CHIR-*Net*) of the German Surgical Society (*Deutsche Gesellschaft für Chirurgie*) and have experience in previous multicenter RCTs. Feasibility evaluation of all participating centers was done according to the SOPs of MSZ. All of the study personnel involved in the trial require GCP training and will be specifically instructed in all trial-specific procedures before initiation of the trial. According to AMG, the investigator requires 2 years' experience in drug trials. The leading surgeon of the operating team will perform the interventions since they represent standard techniques. All participating surgeons will be instructed and authorized by the investigator, prior to the first trial procedure.

9.2 Control and comparators

The WHO published the latest clinical guideline addressing the topic of IOWI in surgery in 2016. The consensus is that there is not sufficient evidence to support the use of IOWI with saline, diluted PVP-solutions should be considered and antibiotic solutions avoided. However, the underlying RCTs included all types of surgery (*i.e.* neuro-, orthopedic surgery.) and are of low level of evidence [6]. The guideline of the British National Institute of Clinical Excellence (NICE) from 2008 [7] states that, due to the lack of evidence any IOWI should be avoided. However, in clinical practice this advice is mostly not being followed. Most hospitals do not have standard protocols but leave the decision to irrigate or not to irrigate the wound up to the surgeon. Given these circumstances it is acceptable to recruit a control group receiving no intervention. So far, no gold standard was determined within RCTs in abdominal surgery. Therefore, the trial proposes an irrigation procedure on the best available evidence, which is either irrigation with PHX-solution or saline or no irrigation. PHX and saline solutions are widely used in clinical practice, but efficacy trials are not available momentarily. As PHX solution is a market-approved drug, safety is ensured and the trial subjects are not exposed to specific risks.

9.3 Additional treatments

No additional treatments will be performed within the trial. Antibiotic treatment 5 days prior to surgery is an exclusion criterion. Pre-operative antibiotic treatment due to septic peritonitis (dirty / contaminated wounds) after admission to the hospital is allowed, but has to be recorded in the CRF. Application of routine intraoperative single shot antibiotic prophylaxis will be recorded in the CRF (type and dose of antibiotics). The application of abdominal wall protectors is recommended for contaminated procedures and has to be recorded in the CRF. A change of gloves ahead of wound closure is recommended for contaminated procedures and has to be recorded in the CRF. If indicated for medical reasons, all kind of medication is permitted during the trial. Postoperative medication with adverse effects on wound healing (*e.g.* corticoids and other immunosuppressive agents) will be recorded in the CRF. Any operative and / or interventional revision of the wound will be documented as AE/ SAE and classified after Clavien Dindo.

9.4 Blinding

The blinding procedure is restricted to participating patients, outcome assessors and the trial statistician. Blinding of the surgical team that performs the intervention is impossible because the control arm does not receive any wound irrigation. A member of the local study team, who will not take part in postoperative patient visits, performs randomization after confirmed closure of the abdominal fascia. A central online randomization tool of the MSZ (RANDOBASE) will effectuate randomization. After informing the surgical team of the result, the investigator has to print out, date and sign the randomization sheets. Subsequently, the randomization sheets have to be stored away from the patient records, trial documents and ISF to ensure blinding of the rest of the local study team.

Postoperatively, a GCP-trained investigator of the local study group, who is unaware of the patient’s intraoperative treatment, will assess wounds on 6 study visits and take a standardized photograph of the wound at each visit which will be uploaded to a central database. However, in case of SSI or any other AE/SAE that has to be reported the local investigator needs to be unblinded.

In addition, independent, blinded outcome-assessors of spatially separated centers participating in the trial will assess the pseudonymized wound photographs of every study visit in a centralized database online. These online outcome-assessors receive training in rating of the primary endpoints according to the CDC classification, which will be documented in a separate training log. These independent outcome-assessors will only access the photo-database for evaluation of the primary endpoint (SSI up to postoperative day 30) and will not be aware of the randomization results or any other patient data. All treatment-specific data are documented in a

separate, undisclosed file. Wound photographs from all trial sites will be assessed by outcome assessors of the coordinating study site in Munich. Photographs from the Munich study site will be assessed in the study site Heidelberg.

9.5Exclusion of participants after initial inclusion

Participants of the study can withdraw their consent to take part at any time without declaration of reasons. All hitherto collected data are subject to analysis. The coordinating investigator or the investigator may exclude patients from the study, if patients' safety is at risk or if there is insufficient compliance of the patient. In order to generate a meaningful database, excluded patients can be replaced by recruitment of new patients. If a patient does not receive PHX or saline irrigation of the wound, this does not automatically lead to exclusion of the study.

10. INCLUSION- AND EXCLUSION CRITERIA

10.1 Inclusion criteria

- Clean-contaminated, contaminated or dirty surgery according to CDC classification (attachment 3);
- Abdominal surgery by midline or transverse laparotomy; elective and emergency procedures;
- Age ≥ 18 years;
- American Society of Anesthesiologists (ASA) score ≤ 3; (attachment 4)
- Ability to understand the nature and extent of the trial and to give written informed consent

10.2 Exclusion criteria

- Pregnancy or breast feeding;
- Known hypersensitivity/allergy to PHX;
- Inability to give/understand informed consent;
- Critical medical condition of emergency patients, precluding informed consent or sufficient time to reflect on the decision to participate in the trial;
- ASA >3;
- Inability to attend follow-up visits;
- Clean procedures according to the CDC classification or surgery without opening of the abdominal cavity;
- Laparoscopic surgery;
- Revision-surgery (previous abdominal surgery within the last 30 days);
- Planned re-laparotomy within 30 days;

- Severe immunosuppression;
- Concurrent abdominal wall infections;
- Pre-operative systemic antibiotic therapy within 5 days prior to surgery (except emergency pre-operative antibiotic treatment due to septic peritonitis after admission to the hospital);
- Participation in another clinical trial that interferes with the primary or secondary outcomes of this trial.

10.3 Explanation of inclusion and exclusion criteria

To enhance generalizability and representativeness, all patients undergoing elective and emergency laparotomy (transverse or midline) for visceral surgery will be screened for this trial. However, only clean-contaminated, contaminated or dirty (class II-IV), open abdominal surgery, according to the CDC classification [1] will be eligible, since in clean (class I) procedures the risk of SSI is low. Laparoscopic surgery as well as surgery without opening of the abdominal cavity or revision surgery (previous abdominal surgery within the last 30 days or planned re-laparotomy within the next 30 days of surgery) will be excluded, since these types of procedures are not comparable in terms of SSI risk.

Pre-operative antibiotic therapy within 5 days prior to surgery was chosen to be an exclusion criterion to avoid bias of the results, since this might lead to a lower individual risk of infection. However, this does not apply to patients that receive pre-operative antibiotics after admission to the hospital in an emergency situation of septic peritonitis. Furthermore, this does not include standard intraoperative single shot antibiotic prophylaxis.

Patients have to be ≥ 18 years of age and able to understand and give written informed consent. Any patient in a very bad general medical condition (ASA > 3) will be excluded to avoid too many patient-related confounders. Emergency patients in a critical medical condition that does not allow them to fully understand and provide informed consent or does not leave them sufficient time to reflect on the decision to participate in the trial will not be included. Furthermore, patients have to be able to attend follow-up visits.

Patients with severe immunosuppression (*e.g.* after: organ or bone marrow transplantation, concurrent steroid treatment with >10 mg prednisone daily or an equivalent dose of any other steroid), concurrent infliximab treatment or treatment with an equivalent immunosuppressive substance, chemotherapy within the last 2 weeks prior to trial intervention) or patients with severe pre-operative neutropenia (≤ 0.5 x 10^9/L) or liver cirrhosis Child-Pugh B/C will not be included. Pregnant or breast feeding women, as well as patients with a known hypersensitivity/allergy to PHX will not be included in the trial either.

Patients that participate in other clinical trials that could interfere with the primary (SSI) or secondary outcomes of the IOWISI trial will be excluded.

11. FREQUENCY AND SCOPE OF TRIAL VISITS

Graph 1 and 2 reflect the intervention scheme, trial flow, and visits for the IOWISI trial. Visits are the same for all participants of the study, regardless the treatment group.

11.1 Recruitment and screening

Only surgical departments with adequate patient numbers, providing a written commitment on their recruitment capacity were included in the trial to reach the target sample size. The recruitment period is set to 27 months (first patient in to last patient out 28 months). In case of elective procedures, pre-screening (this is just a pre-selection of eligible patients within the study team) of patients can be performed up to 14 days prior to the scheduled surgical procedure. Screening and inclusion of patients will be performed not earlier than 3 days and not later than on the day before the planned surgical procedure, to ensure the patient has enough time to consider the decision to participate. In case of emergencies, screening and inclusion can take place on the day of admission to the hospital, which is usually the same day as surgery. All screened patients are documented in a screening log. If patients do not wish to participate in the study, reasons are documented accordingly. If patients fit inclusion/exclusion criteria and agree to participate, they will need to give written informed consent to the local GCP-trained investigator, after adequate time for consideration in order to participate in the study (representing visit 1). Therefore, at the screening visit, a detailed description of the study and further instructions are discussed with the patient, including methods of wound irrigation, risk-benefit-ratio, and follow up schedule.

11.2 Visit 1 (Inclusion)

After the local investigator has reviewed the inclusion and exclusion criteria again and having received written consent by a patient, demographical data / medical history (date of birth [mm/yyyy], gender, body height, body weight, BMI, ASA, medical history, concurrent medication, history of SSI, history of radio/chemotherapy, diabetes, smoking, alcohol consumption, medication, duration of pre-operative hospital stay), diagnosis and the NNIS Risk score for determining the intrinsic risk of SSI (attachment 5) will be documented according to the eCRF. The investigator will perform a physical exam (blood pressure, heart frequency, condition of the planned abdominal surgical incision area, clinical relevant findings [normal or abnormal (please specify), respiratory system, cardiovascular system, liver, kidney, neurological or other free text

and date dd/mm/yyyy]) and take a blood sample (EDTA, Serum, and Citrate). Measurements of the blood sample are:

- Hemoglobin
- Hematocrit
- Platelets
- White blood cell count
- Sodium
- Potassium
- Creatinine
- Non-fasting glucose
- AST/ASAT
- ALT/ALAT
- Bilirubin
- Uric acid
- Prothrombin time (PT)
- Activated partial thromboplastin time (aPTT)
- International normalized ratio (INR)

In case of women of child-bearing potential, a pregnancy test will be performed additionally (serum or urine [negative/positive/not performed with specification of reason as free text]).

11.3 Visit 2 (Surgery/Randomization)

Documented parameters of the surgical procedure include the urgency (emergency/elective), type of surgical procedure (colorectal and/or small bowel and/or,hepato-biliary and/or pancreatic and/or splenectomy and/or gastric and/or esophageal and/or nephrectomy and/or urogenital tract and/or others (freetext)) the duration of surgery (incision until complete skin closure, minutes), the level of contamination according to CDC classification (class II-IV; see attachment 3), the intraoperative use of wound edge protectors (yes/no), and prophylactic changing of gloves during of the operation (yes/no), type (transverse/midline) and length (cm) of the incision, creation of an enterostomy (yes/no), the wound closure technique (subcutaneous sutures (yes/no), stapler/suture, if suture: continuous/single) and used suture material, the administration (yes/no) and timing (>1h/≤1h prior to incision) of antibiotic prophylaxis. If the operating surgeon decides that incomplete closure of the wound and/or any other wound related procedure after the study intervention (e.g. negative pressure treatment) is necessary for the benefit of the patient, the patient will have to be excluded from the trial.

Randomization (see section 24.) will take place at the end of surgery, after closure of the abdominal fascia, when the level of contamination is definitely determined by the surgeon. A

designated member of the local study team (who will not perform postoperative study visits) will perform randomization instantly by using the online tool of the MSZ (RANDOBASE) and inform the surgeon of the result and according treatment. Date of randomization (mm:hh, dd/mm/yyy), successful randomization (yes/no), and the result of the randomization process are documented (Printout). Subsequently, the randomization sheets have to be stored away from the patients file to ensure blinding.

Study treatment according to randomization:

- Wound irrigation with PHX 0,04% 1000ml
- Wound irrigation with NaCl 0,9% 1000ml
- No wound irrigation

Furthermore, any AE or SAE is documented during this visit.

11.4 Visit 3 to 8 (Post-op days 2, 4, 6, 8, 10-14, and 30-36)

Postoperatively, there will be 6 trial visits where an independent, blinded outcome assessor trained in the diagnosis and classification of SSI according to CDC definitions will examine wounds (SSI superficial or deep or organ/space, see attachment 1). In addition, pseudonymized, electronic pictures of the wound will be uploaded to a centralized database for independent and blinded evaluation (see 11.4). The assessors will not be aware of the study procedure or other details of the examined wound photograph. Postoperative medication with adverse effects on wound healing (*e.g.* corticoids and other immunosuppressive agents) will be documented in the eCRF:

In case of SSI, microbiological swabs will be taken from the wound secretion for microbiological differentiation and testing of resistance to antibiotics according to in-house standards by each local institution. Other wound complications like seroma, hematoma, delayed healing or necrosis will be documented as secondary endpoint. In case of any surgical complication, including SSI, will be reported as AE/SAE and the Clavien Dindo classification (attachment 2) will be applied to specify the severity and consequent treatment. Furthermore, the rate of re-operations, mortality and occurrence of any AE or SAE will be documented (see 16). Additionally, the duration of the hospital stay (from admission to discharge or day of the visit, in days) will be documented on visit 8 (post-op day 30-36). To promote complete follow-up, a visit window of 6 additional days was implemented. In addition, patients will be recompensed for any travel expenses needed to attend study visit 8. If however, the patient is unable to attend visit 8 due to postoperative treatment in a rehabilitation facility or other medical reasons, a

standardized protocol for evaluation and documentation of the wound (incl. wound photograph) will be sent to and filled out by the treating physician.

Between post-op day 4 and 8 (visit 4, 5 or 6) one study-specific, post-operative blood sample will be taken, and the same measurements as upon visit 1 will be analyzed according to local clinical routine:

- Hemoglobin
- Hematocrit
- Platelets
- White blood cell count
- Sodium
- Potassium
- Creatinine
- Non-fasting glucose
- AST/ASAT
- ALT/ALAT
- Bilirubin
- Uric acid
- Prothrombin time (PT)
- Activated partial thromboplastin time (aPTT)
- International normalized ratio (INR)

12. DOSE, MODE AND SCHEME OF INTERVENTION

After closure of the abdominal fascia, patients will be randomized stratified by level of contamination of the operation. In the experimental group 1, the subcutaneous soft tissue will be irrigated with 1000 ml of a 0.04% PHX solution, which is the recommended concentration for surgical wound irrigation according to the SMPC. PHX solutions (0.04%) are approved for this indication in Germany. The wound shall be carefully rinsed throughout with the irrigation solution and the excess removed with suction. Debris and blood clots should be removed from the wound using irrigation/suction. The wound shall not be rubbed dry with abdominal cloths, but left moistened with the irrigation solution to ensure sufficient contact time for PHX to have the desired antiseptic effect. After irrigation with PHX the wound shall not be irrigated with saline or any other solution again. Since PHX is a cation-active substance, it is not compatible with anionic organic substances (*e.g.* lactate). Furthermore, the combination of PHX with PVP-I products should be avoided.

In the experimental group 2, the same intervention will be performed using 1000ml of isotonic saline solution (NaCl 0.9%).

The irrigation volume of 1000ml was chosen to be sure that even large laparotomy wounds would be sufficiently irrigated. This was determined by senior surgeons´ clinical experience, since so far no recommendations for the optimal volume of surgical irrigation exist. After irrigation of the wound, the skin closure will be performed according to local standards, without any further wound-related procedure.

In the control group, wounds will not be surgically irrigated, as is currently recommended in the NICE guideline. PHX solutions or saline are to be purchased, stored, and distributed according to the respective trial centers standard operating procedures. Trade name, dosage, batch and dispensed amount will be documented on a separate form.

13. PATIENT, STUDY AND SITE DISCONTINUATION

13.1 Patient discontinuation

Patients have the right to voluntarily withdraw from the study at any time for reason. In addition, the investigator has the right to withdraw a patient from the study at any time. Reasons for withdrawal from the study may include but are not limited to the following:

- Patient withdrawal of consent at any time;
- Any medical condition that the investigator or sponsor determines may jeopardize the patient's safety if he or she continues in the study;
- If it is discovered that a study subject is pregnant or may have been pregnant at the time of intervention (see point 16.9);
- Investigator or sponsor determines it is in the best interest of the patient to discontinue the study.

Every effort should be made to obtain information on patients who withdraw from the study. The primary reason for withdrawal from the study should be documented on the appropriate eCRF. However, patients will not be followed for any reason after consent has been withdrawn. Patients who withdraw from the study will not be replaced.

13.2 Study and site discontinuation

The **sponsor** has the right to terminate this study at any time. Reasons for terminating the study may include but are not limited to the following:

- The incidence or severity of AEs in this or other studies indicates a potential health hazard to patients;

- Unsatisfactory patient enrolment;
- The continuation of study is unethical or it has been proven that the therapy has a clearly negative influence;
- Unforeseen complications arise that no longer justify a continuation of the study;

The **sponsor** will notify the investigator of a decision to discontinue the study. The sponsor has the right to **close a site** at any time.

Reasons for closing a site may include, but are not limited to, the following:

- Excessively slow recruitment;
- Poor protocol adherence;
- Inaccurate or incomplete data recording;
- Non-compliance with the ICH-GCP guideline;
- No study activity (*i.e.* all patients have completed and all obligations have been fulfilled);

The **investigator** can discontinue the clinical study at his site at any time if he no longer considers the continuation of the study, for example if there are ethical and/or medical concerns.

14. ADVERSE EVENTS (AES)

14.1 Definition adverse event (AE)

An AE is any untoward medical occurrence in a patient or in a clinical investigation subject administered a pharmaceutical product, which does not necessarily have a causal relationship with this treatment. An AE can therefore be any unfavorable and unintended sign (including an abnormal laboratory finding), symptom or disease temporally associated with the use of a medicinal product, whether or not related to the treatment. Any AE has to be documented in the eCRF on the respective “Adverse Event Report Form”.

14.2 Specific definitions of AEs in the IOWISI trial

The obligation to document any AE in the study, starts with the randomization and ends with completion of the last study visit. AE/SAEs are documented according to the standard grading on the AE/SAE reporting forms. Surgical site infections (primary endpoint) and all other local wound complications (secondary endpoint) will be documented as AE/SAE. In addition, their severity and the consequent treatment will be documented according to the Clavien Dindo classification (attachment 2). All laboratory values or events that will be assessed as “clinically significant” in the eCRF have to be documented as an AE. The responsible medical investigator will judge the clinical significance in the context of the postoperative course after laparotomy and the correspondent laboratory values before intervention.

14.3 Serious adverse events (SAE) and other definitions

Serious adverse events (SAEs)

A SAE is defined as any clinical event that at any time during the study participation:

- Results in death;
- Is life-threatening (the term life-threatening refers to an event in which the subject was at risk of death at the time of the event and not to an event which hypothetically might have caused death if it was more severe);
- Requires subject hospitalization or prolongation of existing hospitalization;
- Results in persistent or significant disability/ incapacity.
- Results in a congenital anomaly/birth defect or
- Is rated as another significant event or condition by the investigator

Any SAE has to be reported to the MSZ immediately (*i.e.* within 24 hours after becoming aware of the event, see chapter 16.7).

Suspected Unexpected Serious Adverse Reaction (SUSAR)

Serious AEs that are both suspected, *i.e.* possibly related to the investigational medicinal product (IMP) and 'unexpected', *i.e.* the nature and/ or severity of which is not consistent with the applicable product information, are to be classified as Suspected Unexpected Serious Adverse Reactions (SUSARs). If the second assessor classifies the SAR as 'suspected' (the relationship to the IMP is "related", "probable" or "possible") and unexpected, it will be categorized as a SUSAR. All SUSARs are subject to an expedited reporting to the responsible ethics committee(s), the competent federal authority (BfArM) and to all participating investigators (see 16.7). Furthermore, a report on all observed SAEs / SARs / SUSARs will be submitted once a year in the DSUR (Development Safety Update Report) format.

Period of observation and documentation

In this trial, all AEs that occur between the randomization (during surgery) and the last study visit or premature study termination will be documented on the pages provided in the eCRF. AEs must also be documented in the subject's medical records. All subjects who have AEs, whether considered associated with the use of the trial medication or not, must be monitored to determine the outcome. The clinical course of the AE will be followed up until resolution or normalization of changed laboratory parameters or until it has changed to a stable condition.

14.4 Evaluation of the severity

The grading of AEs in this trial will be carried out on the basis of the 5-grade scale defined in the CTCAE V4.0:

Grade 1: Mild AE

Grade 2: Moderate AE
Grade 3: Severe AE
Grade 4: Life-threatening AE or AE causing disablement
Grade 5: Death related to AE

The grading of all AEs listed in the CTCAE v4.0 will be based on the information contained therein. The grading of all other AEs, i.e. those which are not listed in the CTCAE v4.0 will be performed by a responsible investigator, based on definitions given above. In addition, surgical complications will be evaluated according to the Clavien Dindo classification.

14.5 Evaluation of the causal relationship

Investigators will estimate the causal relationship between the AE/SAE and the treatment. When estimating the causality the investigator may draw on known biophysical parameters, incorporate previous knowledge on the AE profile of the investigational product and possible simultaneously factor in the efficacy against other substances and the concomitant diagnoses of the patient. The investigator will categorize each AE that occurred after administration of the IMP regarding the coherency with the administration of the IMP as:

- Related: There is a reasonable possibility that the event may have been caused by the IMP. A certain event has a strong temporal relationship and an alternative cause is unlikely.

- Probable: An AE that has a reasonable possibility that the event is likely to have been caused by the IMP. The AE has a timely relationship and follows a known pattern of response, but a potential alternative cause may be present.

- Possibility: An AE that has a reasonable possibility that the event may have been caused by the IMP. The AE has a timely relationship to the IMP; however, the pattern of response is untypical, and an alternative cause seems more likely, or there is significant uncertainty about the cause of the event.

- Unlikely: Only a remote connection exists between the IMP and the reported AE. Other conditions including concurrent illness, progression or expression of the disease state or reaction of the concomitant medication appear to explain the reported AE.

- Not related: An AE that does not follow a reasonable temporal sequence related to the IMP and is likely to have been produced by the subject’s clinical state, other modes of therapy or other known aetiology.

14.6 Outcome of AEs

The outcome of an AE at the time of the last observation will be classified as:

- Recovered/ Resolved: All signs and symptoms of an AE disappeared without any sequels at the time of the last interrogation.

- Recovering/ Resolving: The intensity of signs and symptoms has been diminishing and/ or their clinical pattern has been changing up to the time of the last interrogation in a way typical for its resolution. Further follow-up is possibly needed.

- Not recovered/ Not resolved: Signs and symptoms of an AE are mostly unchanged at the time of the last interrogation. Further follow-up is possibly needed.

- Recovered/ Resolved with sequels: The patient recovered with sequels from the AE / the AE resolved with sequels, *i.e.* the patient suffers from late complications or damage resulting from the AE.

- **Fatal:** An AE resulting in death. If there are more than one AE only the AE leading to death (possibly, related) will be characterized as 'fatal'.

- **Unknown:** The outcome is unknown or implausible and the information cannot be supplemented or verified.

14.7 Reporting of serious adverse events (SAEs)

Primary reporting of SAEs

All SAEs must be reported immediately, by fax (number 089/4140-6480) by the investigator to the responsible officer at the MSZ using the designated form.

Münchner Studienzentrum
SAE-Reporting
Ismaninger Straße 22
81675 München
Tel.: +49/89/4140-6477
Fax: +49/89/4140-6480

Reporting should under no circumstances take place after more than 24 hours from the moment the investigator becomes aware of the event.

The initial report must be as complete as possible including details of the current illness and SAE and an assessment of the causal relationship between the event and the trial medication.

Second assessment of SAEs

All SAEs will be subject to a second assessment by a designated person. This person is elected by the sponsor and will be independent from the sponsor and the reporting investigator. The second assessor will fill out a 'Second Assessment Form' for each SAE. The 'Second Assessment Form' will contain the following information:

I) Assessment of seriousness of the event (investigator and second assessor)

II) Assessment of relationship between SAE and IMP (investigator and second assessor)

III) Assessment of expectedness of SAE, derived from IMP (second assessor)

IV) A statement if the benefit/ risk assessment for the trial did change as a result of SAE (second assessor)

The responsible safety officer of the MSZ will carry out the expedited reporting. Only SUSARs/ SAEs occurring after administration of IMPs will undergo expedited reporting.

14.8 Expedited reporting

Pursuant to the German Drug Law (AMG) and the GCP Regulation, the ethics committee and the competent federal authority (BfArM) will be informed of all suspected SUSARs and all SAEs resulting in death or being live-threatening occurring during the trial. Both institutions and all participating investigators will be informed in case the risk/ benefit assessment did change or any others new and significant hazards for subjects' safety or welfare occur. The sponsor has to ensure that all relevant information about a SUSAR, which occurs during the course of a clinical trial and is fatal or life threatening is reported as soon as possible and not later than seven days after the sponsor was first aware of the reaction. Any additional relevant information should be sent within eight days of the report. A SUSAR, which is not fatal, or life threatening has to be reported as soon as possible and in any event not longer than 15 days after the sponsor was first aware of the reaction.

14.9 Pregnancy

If, following initiation of the investigational product, it is subsequently discovered that a study subject is pregnant or may have been pregnant at the time of investigational product exposure, the investigator must immediately notify the sponsor of this event via the "Report on the drug exposure during pregnancy" within 24 hours and in accordance with SAE reporting procedures. The patient will be withdrawn from the study. Follow-up information regarding the course of the pregnancy, including perinatal and neonatal outcome and, where applicable, offspring information must be reported on a "Report on the pregnancy outcome during drug exposure". Any pregnancy occurring in a female partner of a male study participant the investigator becomes aware of should be reported to the sponsor. Information on this pregnancy may also be collected on the pregnancy reporting forms.

15. SAFETY MONITORING BOARD (SMB)

An independent Safety Monitoring Board (SMB according to the Guidance E3, ICH note for Guidance E6, ICH note for Guidance E9, Directive 2001/20EC “relating to the implementation of good clinical practice in the conduct of clinical trials on medicinal products for human use) is a group of experts external to the study that addresses the patient’s safety and performs risk / benefit assessments. According to its operating procedures the SMB reviews accumulating safety data from ongoing trials to fulfill the safety monitoring. The rules of the SMB are deposited in the SMB Charta, (SOP_MSZ_AE04-H-A01_V02). The aim of this Charta is to define the composition, responsibilities, purpose and timing of meetings, details of the operation, including documentation and reporting and specifying the procedures to ensure confidentiality and appropriate communication of the SMB.

16. ENSURING DATA QUALITY

16.1 Documentation

All raw data such as patient records are declared as source documents. It must be ensured that they are available during routine monitoring visits. Apart from that the investigator of each site must maintain a separate patient identification list. The patient identification list will be maintained at the site separate from the documentation. The eCRF covers all the important forms, sorted according to visits. If a patient withdraws from the study, the reason must be recorded on the eCRF.

Data collection

The documentation of the study data in adherence to the GCP-guidelines and the clinical trial protocol is the responsibility of the investigator. Original data (source documents) remain in hospital medical record and information on the eCRF must be traceable and consistent with the original data. Source documents are *e.g.* laboratory results, photography, skin biopsy histology description and quality of life questionnaire, EASI, Pruritus VAS, TSQM. Original written informed consent signed by the patient is kept by the investigator and a signed copy will be given to the patient. No information in source documents about the identity of the patients will be disclosed. All data collected in this study must be entered in an eCRF which has to be completed by the investigator or authorized trial personnel and signed by the investigator. This also applies for those patients who do not complete the study. If a patient withdraws from the study, the reason must be recorded on the eCRF. The investigator is responsible for ensuring the accuracy, completeness, and timeliness of all data reported to the sponsor in the eCRFs and in all required reports.

Database management

Data are administered and processed by data management of the MSZ with the support of a study database (eCRF) according to the SOPs of the MSZ. A description of the study specific processes is given in the Data Management Plan that details the key planning and control elements for the data management component of the study.

The evaluation of the data takes place by programmed validity- and consistency checks. In addition a manual/visual evaluation of plausibility is performed in accordance to the requirements of GCP. Queries may occur, which will be visualized on the study database. The investigator has to resolve all data discrepancies in the study database. After entry of all collected data and clarification of all queries, the database will be closed at the completion of the study. The database closure has to be documented. Data and results electronically recorded will be archived according to legal guidelines at least 10 years after study termination.

16.2 Audits and inspections

As part of quality assurance according to GCP, the sponsor and the competent health authorities have the right to audit/inspect the study sites and any other institutions involved in the trial. The aim of an audit/inspection is to verify the validity, accuracy and completeness of data, to establish the credibility of the clinical trial, and to check whether the trial subject's rights and trial subject safety are being maintained. The sponsor may assign these activities to persons otherwise not involved in the trial (auditors). These persons as well as inspectors are allowed to access all trial documentation (especially the trial protocol, eCRFs, trial subjects' medical records, drug accountability documentation, and trial-related correspondence).

The sponsor and all investigators of the participating study sites undertake to support auditors and inspections by the competent authorities at all times and to allow the persons charged with these duties access to the necessary original documentation. All persons conducting audits undertake to keep all trial subject data and other trial data confidential.

After each external audit the investigator receives an audit confirmation from the responsible auditor. This confirmation has to be stored in the ISF in order to provide access to it in case of an inspection by the competent authorities. The audit report is provided to the sponsor for control.

16.3 Monitoring

Monitoring activities are performed to ensure that the trial is conducted in accordance with the trial protocol, the principles of GCP and local legislation. A monitoring manual describing the scope of the monitoring activities in detail will be prepared.

The responsible monitor will contact the investigator and will be allowed, on request, to inspect the various records of the trial (eCRF and other pertinent data) provided that patient confidentiality is maintained in accord with local requirements. The monitor should have access

to patient records, any information needed to verify the entries in the eCRF and all necessary information and essential study documents. The investigator agrees to cooperate with the monitor to ensure that any problems detected in the course of these monitoring visits are resolved. A monitoring visit report is prepared for each visit describing the progress of the clinical trial and all identified problems.

16.4 Archiving

At the end of the clinical study all study-relevant data must be archived as required by law and when indicated in addition according to the Clinical Trial Agreement. All documentation forms, ICFs and other essential study documents must be retained as required by law. Patient ID lists and patient files are retained in the respective study sites separately. The ICFs are kept in with the study documents.

17. ETHICAL AND REGULATORY ASPECTS

17.1 Sponsor‘s and investigator’s responsibilities

This study is conducted in compliance with all applicable laws and regulations and also the Declaration of Helsinki. **The sponsor has the overall responsibility for the ethical and scientific conduct of the study. All participating investigators agree to adhere to the instructions and procedures described in the study protocol and thereby to adhere to the principles of GCP that it conforms to.**

The responsible ethics committee of TUM and health authority (BfArM) will review the final study documents. The ethics committee's and BfArM’s decision concerning the conduct of the study will be communicated in written form to the sponsor. The sponsor will assure submission of required progress reports, annual safety reports and substantial amendments for approval to the ethics committee and BfArM. Before initiating the study, the sponsor must submit any required amendments to BfArM for review and acceptance to begin the trial according to § 42 AMG. Furthermore, the sponsor has to inform the ethics committee and BfArM within 90 days about completion of the trial and provide a brief report of its outcome 1 year after completion of the trial. Results of the study will be reported following ICH-GCP-E6 and published according to the CONSORT statement.

17.2 Independent ethics committees and health authorities

Prior to the start of this study, the protocol and other required documents would have to be reviewed and approved by the locally responsible ethics committees of each study site. Their reports as well as a signed and dated approval by the BfArM must be obtained and assessed by the leading ethics committee of the TUM before study initiation. Any amendments to the

protocol, other than administrative ones (of which the leading ethics committee and BfArM will merely be informed), must be reviewed and approved by both authorities.
Before inclusion of the first patient the federal state authorities (*zuständige Regierungsbehörden der Länder*) will be informed about the study. A copy of this report needs to be forwarded to BfArM and needs to be filed in the ISF and TMF.

17.3 Ethical performance of the study

The study is conducted according to the ethical principles as defined in the Declaration of Helsinki, version of 2008 (see 28.). The present clinical study is conducted in accordance with principles published in the ICH-GCP Guideline and the applicable legal regulations (AMG, GCP-V, see 19.1) These principles concern ethics committee procedures, patient information and informed consent procedures, adherence to the protocol, administrative documents, documentation of the study medication, data collection, patient records (source documents), recording and reporting of AEs/SAEs, preparation of inspections and audits as well as storage and safekeeping of the documents. All the investigators and personnel involved in the study have been informed that international monitoring authorities, the competent federal authorities and the sponsor are authorised to review the study documents and patient files.

17.4 Public register

Before the clinical study will be initiated, it will be filed at the German Clinical Trials Register (DRKS), which is part of the International Clinical Trials Registry Platform (ICTRP) of the WHO. After ethical approval the trial will be registered under the ID-number: DRKS00012251.

17.5 Informed consent of the study participants

A patient can only be included in the study, if he provides written consent after being informed by a GCP-trained investigator (orally and in writing) about the nature, significance and scope of the clinical study in an appropriate and understandable way. The investigator must fully explain the purpose of the study to the patient or his/her guardian prior to entering the patient into the study. The investigator is responsible for obtaining written informed consent from each patient The person signing the consent form will receive a copy of the signed form. By providing such consent the patient is declaring that he understands and accepts the recording of data that is part of the study and its verification by authorised monitors or federal authorities. The patient will be educated about the potential benefits and complications of the IMP used in the study. It must be clear for him that he can withdraw his consent at any point of time without any disadvantages to his further treatment. The original copy of the written ICF will be kept in the study folder of the study site. The patient will be given the copies of the written patient information and ICF. Additionally, copies of both documents will be filed in the patient’s medical file. Patient

information and ICF are attached at the end of this protocol. The patient information and ICF will be submitted to the responsible ethics committee for assessment before the study will be initiated.

18. INSURANCE FOR TRIAL PARTICIPANTS

In the clinical trial of an IMP, all the participants are insured in accordance with the AMG. The scope of the insurance coverage is derived from the insurance documents that are included in the ISF. Before inclusion the insurance conditions shall be submitted to the patient for review without request to do so. The insurance conditions should be furnished to the patient to take with him before being included in the study on request and after inclusion in any case. Insurance coverage is being provided by:

HDI-Gerling Industrie Versicherung AG
Niederlassung München
Vertragsservice/M-B
Ganghofer Strasse 37-39
80339 Munich
Tel.: +49 (89) 9243-420
Fax.: +49 (89) 9243-356
Insurance number: 65-963496-03037/390 (Studie: 2/17)

If an insured event is suspected to have occurred, the sponsor is to be notified immediately. He then has to notify the insurance provider about the damages immediately. The patient will receive a copy of the notification to the insurance provider. The patient may also inform the insurance provider by bypassing the study personnel, reporting any claims. In this case, he should be notified that the sponsor of the clinical trial should still be informed about the event. Patients have to be informed about both options.

19. DATA PRIVACY PROTECTION / CONFIDENTIALITY PROTECTION

The applicable local regulations of data privacy protection will be followed. The patients will be informed that any patient-related data and materials will be appropriately made pseudonymous (pursuant to § 12 and § 13 of the GCP Regulations) and that these data may be used for analysis and publication purposes. Furthermore, the patients will be informed that their data may be inspected by representatives of BfArM or of the sponsor for the purpose of validation of a proper study conduct. Patients who do not provide consent for transmission of their data, according to the data protection agreement included in the ICF, will not be included in the clinical study.

20. PROTOCOL AMENDMENTS OR CHANGES IN TRIAL CONDUCT

In order to insure comparable conditions in all study sites and in the interest of standardized evaluations of the trial, changes in this protocol are not foreseen. However, changes in trial conduct are possible. Any change (besides administrative changes) of this protocol requires a written protocol amendment that must be reviewed by the sponsor before implementation. Furthermore, consent needs to be obtained by the investigator of each participating center. Amendments that significantly affect the safety of subjects, the scope of the investigation or the scientific quality of the study, additionally require approval of the leading ethics committee and BfArM. A copy of the written approval of these amendments must be provided to the sponsor and the investigator at each study site. Examples of amendments requiring such approval are:

- Significant changes in the study design;
- Increases in the number of invasive procedures.

However, these requirements for approval should in no way prevent the investigator or sponsor to take any immediate action in the interests of preserving patient safety. If the investigator feels an immediate change to the protocol is necessary and is implemented for safety reasons, the sponsor, ethics committee and BfArM must be informed immediately. Amendments affecting only administrative aspects of the study do not require formal protocol amendments or ethics committee and BfArM approval. However, the ethics committee and BfArM must still be notified about the changes.

21. STATISTICAL CONSIDERATIONS

21.1 Proposed sample size / Power calculations

The sample size was calculated (nQuery Advisor software version 7.0, Statistical Solutions Ltd, Cork, Ireland) based on the primary endpoints of the study, assuming SSI rates of 2.2% in the PHX group (assuming a 75% risk reduction according to the trial by Roth *et al*. [1]), 8.7% in the saline group (according to the results of the trial by Cervantes-Sanchez *et al.*[2]), and 16.2% in the control group (according to the meta-analysis by Mueller *et al*. [3]). The global significance level was set to 5%. Since the PHX arm will be used twice for a comparison, the Bonferroni-Holm procedure was used to set the local alpha level for test 1 (PHX *vs.* saline irrigation) to 2.5% and for test 2 (PHX *vs.* no intervention) to 5%. If 230 patients are recruited in the PHX arm, 230 patients in the saline arm and 80 patients in the control arm (a total of 540 patients), the two-sided Fisher exact test for test 1 will have a power of 94% and for test 2 – a power of 85% to detect differences between the treatment groups. The comparison saline irrigation *vs.*

control is not included in the sample size calculation, as it will not be analyzed in a confirmatory manner. The low medical interest cannot justify the large increase in patient numbers.

An interim analysis is not necessary since patients in this trial do not undergo any specific additional risks, as all products used are on-label in Germany. Baseline adjustments will be performed according to the pre-specified subgroup analyses. No dropout rates are calculated, as the analysis will be based on the ITT set.

21.2 Statistical analysis

The primary and secondary endpoints will be analyzed on the Intention-To-Treat (ITT) set, consisting of all patients included in the study in the treatment arm they were randomized to. The safety analysis will be performed on the safety set, consisting of all patients randomized into the study and assigned to the treatment group of their actual treatment.

21.3 Primary endpoint

Wound irrigation with PHX solution will be tested for superiority over no irrigation (Test 1) and irrigation with saline (Test 2) with respect to the incidence of SSI within 30 days of surgery using two Fisher exact tests with the following hypotheses:

Test 1: $H_{1\_0}: \pi_P = \pi_N$ vs. $H_{1\_A}: \pi_{P\neq}\pi_N$

Test 2: $H_{2\_0}: \pi_P = \pi_S$ vs. $H_{2\_A}: \pi_P \neq \pi_S$

Where π_P,π_N, and π_S denote the incidence of SSI within 30 days of surgery in the PHX, no irrigation, and saline groups respectively. The tests will be performed two-sided and with a global significance level of 5%. Using the Bonferroni-Holm adjustment, the local significance level for Test 1 will be 2.5% and for Test 2 it will be 5%.

21.4 Supportive analysis of the primary endpoint

Since randomization will be stratified by study center and level of contamination, supportive analysis of the primary endpoint will also be performed using a binary logistic regression model with dependent variable SSI and covariates treatment group, study center, and level of contamination. In case there are differences between the treatment groups in terms of baseline characteristics, those will also be included as covariates in the model. Operation related risk factors (*e.g.* type and duration of surgery, administration and timing of antibiotic prophylaxis, use of wound-edge protectors, intraoperative changing of gloves, presence of an ostomy) and patient related risk factors (*e.g.* NNIS risk score, ASA, BMI, age, diabetes, smoking, alcohol consumption, duration of preoperative hospital stay, history of SSI, history of

radio/chemotherapy) might influence the outcome, which is why they will also be included as model covariates.

21.5 Secondary endpoints

Secondary endpoints will be analyzed by treatment group on the ITT set, using appropriate descriptive statistics. Any explorative statistical testing will be performed using a significance level of 5%. Subgroup analyses or treatment group comparisons will be performed for rate of superficial/deep/organ space SSI (according to CDC [1], attachment 1) stratified by the NNIS risk score and by level of contamination (class II,III or IV) during surgery (according to CDC [1] attachment 3). All AEs including SSI and local wound complications will be analyzed with incidence rates by treatment group and according to severity. AEs rated as related to the study treatment will be listed separately. In addition, the duration of hospital stay in days will be compared between the three study groups.

21.6 Missing data

First analysis will be based on all patients with complete follow-up. For sensitivity, multiple imputations will be used for missing primary endpoint data. A dropout rate of 8-10% is expected in this study.

22. RANDOMIZATION AND METHODS AGAINST BIAS

Participating, GCP-certified investigators will perform the screening and recruitment of patients and will obtain the ICF prior to inclusion. Every patient fulfilling inclusion and exclusion criteria will be documented. Reasons for non-inclusion into the study will have to be documented as well in a screening-list. A GCP-trained member of the study group will perform randomization during surgery after closure of the abdominal fascia is completed using RANDOBASE, the online-randomization tool at MSZ. RANDOBASE uses pre-defined randomization lists, which will be created at IMSE and will be stratified by level of contamination of the surgical procedure (clean-contaminated, contaminated or dirty) and by study center. To assure balanced group sizes in the course of the accrual, a block-wise randomization is applied. Basic characteristics of the patient and day of randomization must be documented on the randomization sheets. Subsequently, randomization sheets must be printed out, dated, signed and stored away from the patient records, trial documents and ISF to ensure blinding. Details on the blinding procedure are presented under point 11.4.

23. FINAL REPORTING

After completion of the trial, BfArM and the leading ethics committee (TUM) have to be informed within 90 days by a final study report. Within one year of the completion of the trial, BfArM and the ethics committee will be supplied with a summary of the final report on the clinical trial containing the principle results. The sponsor is responsible for the generation of these final reports.

24. PUBLICATION OF STUDY RESULTS

After completion of the clinical study, a multi-center manuscript of the study results will be prepared for publication in a reputable scientific journal according to the CONSORT statement. For this manuscript, final analyses will be generated from the study database and it will be subject to review by the sponsor. The publication of the principal results from any single center experience within the trial is not allowed until the preparation and publication of the multi-center results. Exceptions to this rule require prior approval of the sponsor. For purposes of abstract presentation and publication, any secondary publications will be delegated to the appropriate principal authors. However, final analyses and manuscript review for all multi-center data will require the approval of the sponsor. The use of professional writers is not intended. Details on publication rules and author order will be provided in the Clinical Trial Agreement.

25. DECLARATION OF HELSINKI

The Declaration of Helsinki, 2008 (Seoul), is attached to the protocol.

26. ATTACHMENTS

Attachment 1: Definition and classification of SSI according to CDC

**Superficial Incisional SSI**	Infection occurs within 30 days after the operation **and** infection involves only skin or subcutaneous tissue of the incision **and** at least ***one*** of the following:<br><br>1 Purulent drainage, with or without laboratory confirmation, from the superficial incision.<br>2 Organisms isolated from an aseptically obtained culture of fluid or tissue from the superficial incision.<br>3 At least one of the following signs or symptoms of infection: pain or tenderness, localized swelling, redness, or heat *and* superficial incision is deliberately opened by surgeon, *unless* incision is culture-negative.<br>4 Diagnosis of superficial incisional SSI by the surgeon or attending physician.<br><br>*Notes:*<br>Do *not* report the following conditions as SSI:<br>1 Stitch abscess (minimal inflammation and discharge confined to the points of suture penetration).
**Deep Incisional SSI**	Infection occurs within 30 days after the operation **and** the infection appears to be related to the operation **and** infection involves deep soft tissues (*e.g*. fascial and muscle layers) of the incision **and** at least *one* of the following:<br><br>1 Purulent drainage from the deep incision but not from the organ/space component of the surgical site.<br>2 A deep incision spontaneously dehisces or is deliberately opened by a surgeon when the patient has at least one of the following signs or<br>3 Symptoms: fever (>38°C), localized pain, or tenderness, unless site is culture-negative.<br>4 An abscess or other evidence of infection involving the deep incision is found on direct examination, during reoperation, or by histopathologic or radiologic examination.<br>5 Diagnosis of a deep incisional SSI by a surgeon or attending physician.<br><br>*Notes:*<br>1 Report infection that involves both superficial and deep incision sites as deep incisional SSI.<br>2 Report an organ/space SSI that drains through the incision as a deep incisional SSI.
**Organ/Space SSI**	Infection occurs within 30 days after the operation and the infection appears to be related to the operation **and** infection involves any part of the anatomy (*e.g.,* organs or spaces), other than the incision, which was opened or manipulated during an operation **and** at least *one* of the following:<br><br>1 Purulent drainage from a drain that is placed through a stab wound into the organ/space.<br>2 Organisms isolated from an aseptically obtained culture of fluid or tissue in the organ/space.<br>3 An abscess or other evidence of infection involving the organ/space that is found on direct examination, during reoperation, or by histopathologic or radiologic examination.<br>4 Diagnosis of an organ/space SSI by a surgeon or attending physician.

Attachment 2: Clavien Dindo classification of surgical complications

Grade	Definition
Grade I	Any deviation from the normal postoperative course without the need for pharmacological treatment or surgical, endoscopic, and radiological interventions
Grade II	Allowed therapeutic regimens are: drugs as antiemetics, antipyretics, analgetics, diuretics, electrolytes, and physiotherapy. This grade also includes wound infections opened at the bedside
Grade III	Requiring pharmacological treatment with drugs other than such allowed for grade I complications Blood transfusions and total parenteral nutrition are also included Requiring surgical, endoscopic or radiological intervention
Grade IIIa	Intervention not under general anesthesia
Grade IIIb	Intervention under general anesthesia
Grade IV	Life-threatening complication (including CNS complications)* requiring IC/ICU management
Grade IVa	Single organ dysfunction (including dialysis)
Grade IVb	Multiorgan dysfunction
Grade V	Death of a patient
Suffix “d”	If the patient suffers from a complication at the time of discharge, the suffix “d” (for “disability”) is added to the respective grade of complication. This label indicates the need for a follow-up to fully evaluate the complication.
	*Brain hemorrhage, ischemic stroke, subarrachnoidal bleeding, but excluding transient ischemic attacks. CNS, central nervous system; IC, intermediate care; ICU, intensive care unit

Attachment 3. Classification of wound contamination levels according to CDC

Class I/ Clean	These are uninfected operative wounds in which no inflammation is encountered and the respiratory, alimentary, genital, or uninfected urinary tracts are not entered. In addition, clean wounds are primarily closed, and if necessary, drained with closed drainage. Operative incisional wounds that follow non-penetrating (blunt) trauma should be included in this category if they meet the criteria. Laparoscopic surgeries, surgeries involving the skin (such as biopsies), eye or vascular surgeries are good examples.
Class II/ Clean-Contaminated	**An operative wound in which the respiratory, alimentary, genital, or urinary tracts are entered under controlled conditions and without unusual contamination.** Specifically, operations involving the biliary tract, appendix, vagina, and oropharynx are included in this category, provided no evidence of infection or major break in technique is encountered.
Class III/ Contaminated	Open, fresh, accidental wounds. In addition, **operations with major breaks in sterile technique (e.g., open cardiac massage) or gross spillage from the gastrointestinal tract, and incisions in which acute, non-purulent inflammation is encountered are included in this category**. Contaminated wounds are also created when an outside object comes in contact with the wound (e.g. a bullet, knife blade or other pointy object).
Class IV/ Dirty-Infected	Old traumatic wounds with retained devitalized tissue and those that involve existing clinical infection or perforated viscera or a foreign object lodged in the wound **or any wound that has been exposed to pus or fecal matter.** This definition suggests that the organisms causing postoperative infection were present in the operative field before the operation.

This classification scheme has been shown in numerous studies to predict the relative probability that a wound will become infected. Clean wounds have a 1-5% risk of infection; clean-contaminated 3-11%; contaminated, 10-17%; and dirty over 27% (CDC).

Attachment 4: ASA classification

ASA Score	Patient's Preoperative Physical Status
1	Normally healthy patient
2	Patient with mild systemic disease
3	Patient with severe systemic disease that is not incapacitating
4	Patient with an incapacitating systemic disease that is a constant threat to life
5	Moribund patient who is not expected to survive for 24 hours with or without operation

Attachment 5: NNIS risk index

The NNIS risk index is operation-specific and applied to prospectively collected surveillance data. The index values range from 0 to 3 points and are defined by three independent and equally weighted variables. 0 indicating the lowest and 3 the highest risk of SSI.

One point is scored for each of the following when present:

(1) American Society of Anesthesiologists (ASA) Physical Status Classification of >2
(2) Either contaminated or dirty/infected wound classification (class III and IV)
(3) Length of operation >T hours, where T is the approximate 75th percentile of the duration of the specific operation being performed.

The T Point for Common Surgical Procedures (NNIS report 2004)

Operation	T Point (hrs)
Bile duct, liver, or pancreatic surgery	4
Colonic surgery	3
Herniorrhaphy	2
Appendectomy	1
Other digestive	2
Laparotomy	2
Small bowel	3
Splenectomy	3
Cholecystectomy	2
Gastric	3
Nephrectomy	4
Organ transplant	6

27. REFERENCES

1. Mangram, A.J., et al., *Guideline for Prevention of Surgical Site Infection, 1999. Centers for Disease Control and Prevention (CDC) Hospital Infection Control Practices Advisory Committee.* Am J Infect Control, 1999. **27**(2): p. 97-132; quiz 133-4; discussion 96.
2. Mihaljevic, A.L., et al., *Multicenter Double-Blinded Randomized Controlled Trial of Standard Abdominal Wound Edge Protection With Surgical Dressings Versus Coverage With a Sterile Circular Polyethylene Drape for Prevention of Surgical Site Infections: A CHIR-Net Trial (BaFO; NCT01181206).* Ann Surg, 2014. **260**(5): p. 730-9.
3. Diener, M.K., et al., *Effectiveness of triclosan-coated PDS Plus versus uncoated PDS II sutures for prevention of surgical site infection after abdominal wall closure: the randomised controlled PROUD trial.* Lancet, 2014. **384**(9938): p. 142-52.
4. Pinkney, T.D., et al., *Impact of wound edge protection devices on surgical site infection after laparotomy: multicentre randomised controlled trial (ROSSINI Trial).* Bmj, 2013. **347**: p. f4305.
5. Barnes, S., et al., *Surgical wound irrigation: A call for evidence-based standardization of practice.* American Journal of Infection Control, 2014. **42**(5): p. 525-529.
6. *Global Guidelines for the Prevention of Surgical Site Infection*. 2016, World Heath Orgnaization: Geneva.
7. *National Institute for Health and Clinical Excellence (NICE). Surgical Site Infection - Prevention and Treatment of Surgical Site Infection. NICE Clinical Guideline 74.* 2008, RCGO Press: London, England.
8. Rohner, E., et al., *Preferred use of polyhexanide in orthopedic surgery.* Orthopedics, 2011. **34**(10): p. e664-8.
9. Roth, B., et al., *[Recommendations for the use of polyhexanide-containing products for the treatment of wounds].* Praxis (Bern 1994), 2011. **100**(9): p. 531-7.
10. Fournel, I., et al., *Meta-analysis of intraoperative povidone-iodine application to prevent surgical-site infection.* Br J Surg, 2010. **97**(11): p. 1603-13.
11. Chundamala, J. and J.G. Wright, *The efficacy and risks of using povidone-iodine irrigation to prevent surgical site infection: an evidence-based review.* Can J Surg, 2007. **50**(6): p. 473-81.
12. Charalambous, C.P., et al., *When should old therapies be abandoned? A modern look at old studies on topical ampicillin.* J Infect, 2003. **47**(3): p. 203-9.
13. McHugh, S.M., et al., *The role of topical antibiotics used as prophylaxis in surgical site infection prevention.* J Antimicrob Chemother, 2011. **66**(4): p. 693-701.
14. Mueller, T.C., et al., *Intra-operative wound irrigation to reduce surgical site infections after abdominal surgery: a systematic review and meta-analysis.* Langenbecks Arch Surg, 2015.
15. Cervantes-Sanchez, C.R., et al., *Syringe pressure irrigation of subdermic tissue after appendectomy to decrease the incidence of postoperative wound infection.* World J Surg, 2000. **24**(1): p. 38-41; discussion 41-2.
16. Roth B, A.O., Wurmitzer F, Kramer A, *[Surgical site infections after primary antiseptic cleansing of dirty-contaminated wounds by polihexanide, PVP iodine resp. hydrogen peroxide].* GMS Krankenhaushyg Interdiszip 2007. **2**(2): p. Doc58.
17. Wundinfektionen, K.S.S.p. 2013 [cited 2014; Available from: http://www.nrz-hygiene.de/surveillance/kiss/.
18. de Lissovoy, G., et al., *Surgical site infection: incidence and impact on hospital utilization and treatment costs.* Am J Infect Control, 2009. **37**(5): p. 387-97.
19. Gastmeier, P., et al., *Postoperative Wundinfektionen nach stationären und ambulanten Operationen.* Bundesgesundheitsblatt - Gesundheitsforschung - Gesundheitsschutz, 2004. **47**(4): p. 339-344.
20. Geffers, C., M.D., *Postoperative Wundinfektionen*, B.M. e.V., Editor. 2011, Institut für Hygiene und Umweltmedizin der Charité, Berlin.
21. Mihaljevic, A.L., et al., *Wound edge protectors in open abdominal surgery to reduce surgical site infections: a systematic review and meta-analysis.* PLoS One, 2015. **10**(3): p. e0121187.
22. Clavien, P.A., et al., *The Clavien-Dindo classification of surgical complications: five-year experience.* Ann Surg, 2009. **250**(2): p. 187-96.
23. *Gesetz über den Verkehr mit Arzneimitteln (Arzneimittelgesetz - AMG)*. 2005, Bundesministerium für Justiz und Verbraucherschutz.
24. *International Conference on Harmonisation of Technical Requirements for Registration of Pharmaceuticals for Human Use, ICH E6*. 1996, International Conference on Harmonisation; http://www.ich.org: Geneva.
25. Chan, A.W., et al., *SPIRIT 2013 statement: defining standard protocol items for clinical trials.* Ann Intern Med, 2013. **158**(3): p. 200-7.

1.2 CSP final version 3.0 (02.03.2021)

TECHNISCHE UNIVERSITÄT MÜNCHEN   KLINIKUM RECHTS DER ISAR

STUDY PROTOCOL

IOWISI

Intraoperative wound irrigation to prevent surgical site infection after laparotomy

Sponsor:

Technische Universität München (TUM)
Fakultät für Medizin
Represented by the Dean
Ismaninger Str. 22
81675 München, Germany

Sponsor Delegated Person (SDP):

Prof. Dr. med. Daniel Reim
Klinik und Poliklinik für Chirurgie
Klinikum rechts der Isar TUM
Ismaninger Str. 22
81675 München, Germany

EudraCT Number: 2017-000152-26   **Study Code:** IOW-1755-REI-0540-I

DRKS Number: DRKS00012251

Version: 3.0 AM2.0   **Date** 02.03.2021

! Confidential !
This document is confidential and should serve as a source of information for Investigators and other personnel involved in this clinical study, consultants and ethics committees and regulatory authorities. The contents of this document shall only be disclosed to others in agreement with the coordinating investigator and/or sponsor.

Responsibility

Protocol development	**Sponsor**	**Technische Universität München (TUM), Fakultät für Medizin**<br>**Represented by the Dean**<br>Ismaninger Str. 22<br>81675 München<br>Tel.: 089 4140-4020<br>Fax: 089-4140-4935<br>E-Mail: dekanat.medizin@tum.de
	**SDP, Coordinating investigator (LKP)**	**PD Dr. med Daniel Reim**<br>**Klinik und Poliklinik für Chirurgie**<br>Klinikum rechts der Isar der TUM<br>Ismaninger Str. 22<br>81675 München<br>Tel.: 089 4140-5019<br>Email: daniel.reim@tum.de
	**Clinical project management**	**Dr. med. Tara Mueller**<br>**Klinik und Poliklinik für Chirurgie**<br>Klinikum rechts der Isar der TUM<br>Ismaninger Str. 22<br>81675 München<br>Tel.: 089 4140-2145<br>Email: tara.mueller@tum.de
	**Trial statistican**	**Dr. rer. nat. Victoria Kehl**<br>TUM, Fakultät für Medizin, Münchner Studienzentrum (MSZ)<br>Ismaninger Str. 22<br>81675 München<br>Tel: 089 4140-4355<br>Fax: 089 4140-4850<br>Email: victoria.kehl@tum.de
	**Project management**<br>**Monitoring**<br>**Data management**<br>**Safety management**	**TUM, Fakultät für Medizin, MSZ**<br>Ismaninger Str. 22<br>81675 München<br>Tel: 089 4140-6321<br>Fax: 089 4140-6322<br>Email:<br>muenchner.studienzentrum@mri.tum.de

28. DECLARATION OF INVESTIGATOR

I have read the trial protocol and I confirm that it contains all information to accordingly conduct the clinical trial. I pledge the clinical trial will be conducted at my trial center according to the protocol.

The first patient will be enrolled only after all ethical and regulatory requirements are fulfilled. I pledge that written informed consent for trial participation will be obtained from all patients.

I know the requirements for accurate notification of serious adverse events and I pledge to document and notify such events as described in the protocol.

I pledge to retain all trial-related documents and source data as described. All necessary documents will be provided before trial start. I agree that these documents will be submitted to the responsible regulatory authorities and ethics committees.

29. SYNOPSIS

**Sponsor**	Technische Universität München, Fakultät für Medizin
**Name of the trial**	Intraoperative wound irrigation to prevent surgical site infection after laparotomy - IOWISI
**Trial design**	Prospective, randomized, controlled, observer and patient-blinded, multicenter, surgical trial according to German drug law (AMG) phase IIIb, with three parallel comparison groups
**Objectives**	To investigate whether the use of intraoperative, epifascial wound irrigation with polyhexanide (PHX) solution can reduce surgical site infections after laparotomy for visceral surgery compared to saline irrigation or no irrigation.
**Interventions**	<u>Experimental intervention/index test:</u><br>• Intervention 1: Irrigation of the subcutaneous tissue after closure of the abdominal fascia with 1000ml PHX solution (0.04%)<br>• Intervention 2: Irrigation of the subcutaneous tissue after closure of the abdominal fascia with 1000ml saline solution (NaCl 0.9%)<br><u>Control intervention/reference test:</u><br>No epifascial wound irrigation<br><u>Follow-up per patient</u>:<br>Postoperative day 30 (+6 at the latest)<br><u>Duration of intervention per patient</u>:<br>One intraoperative application<br><u>Experimental and/or control off-label or on-label in Germany</u>:<br>All interventions are on-label in Germany
**Key inclusion and exclusion criteria**	<u>Key inclusion criteria</u>:<br>• Clean-contaminated, contaminated or dirty surgery (class II-IV) according to Centre for Disease Control (CDC) classification;<br>• Abdominal surgery by midline or transverse laparotomy; elective and emergency procedures;<br>• Age ≥ 18 years;<br>• American Society of Anesthesiologists (ASA) score ≤ 3;<br>• Ability to understand the nature and extent of the trial and to give written informed consent;<br><u>Key exclusion criteria</u>:<br>• Pregnancy or breast feeding;<br>• Known hypersensitivity/allergy to PHX;<br>• Inability to give/understand informed consent;<br>• Critical medical condition of emergency patients, precluding informed consent or sufficient time to reflect on the decision to participate in the trial;<br>• ASA >3;<br>• Inability to attend follow-up visits;<br>• Clean procedures according to the CDC classification or surgery without opening of the abdominal cavity;<br>• Laparoscopic surgery;<br>• Revision-surgery (previous abdominal surgery within the last 30 days);<br>• Planned re-laparotomy within 30 days;<br>• Severe immunosuppression;<br>• Concurrent abdominal wall infections;<br>• Pre-operative systemic antibiotic therapy within 5 days prior to surgery (except emergency pre-operative antibiotic treatment due to septic peritonitis after admission to the hospital);<br>• Participation in another clinical trial that interferes with the primary or secondary outcomes of this trial.
**Outcomes**	<u>Primary efficacy endpoint</u>:<br>SSI according to CDC criteria within 30 days postoperatively<br><u>Key secondary endpoint(s)</u>:<br>• Non-infectious wound complications (*e.g.* seroma, hematoma, delayed healing) within 30 days postoperatively

	• Duration of hospital stay<br>• Mortality and morbidity within 30 days postoperatively<br>• Incidence of reoperation within 30 days postoperatively<br>• Incidence of AE/SAE within 30 days postoperatively<br>Pre-specified subgroup analysis by category of SSI (superficial, deep, organ space), NNSI risk score, ASA score, BMI, age, diabetes, smoking, alcohol consumption, history of SSI, history of radio-/chemotherapy, pre-operative hospital stay >2d, administration and timing of antibiotic prophylaxis, type and duration of surgery, intraoperative use of wound-edge protectors and changing of gloves, presence of an enterostomy.<br>Safety: Adverse events (AE) and serious adverse events (SAE) are documented for all groups. Surgical complications will be additionally evaluated according to the Clavien-Dindo classification
**Study registry**	German CTR (DRKS): DRKS00012251 / EudraCT: 2017-000152-26
**Statistical analysis**	Efficacy: The incidence of SSI within 30 days after surgery will be compared between three study groups in two ways:<br>Test 1: PHX irrigation *vs*. no intervention<br>Test 2: PHX irrigation *vs.* saline irrigation<br>Description of the primary efficacy analysis and population:<br>The incidence of SSI within 30 days of surgery will be compared in test 1 and test 2 using two Fine and Gray subdistributional hazard models with SSI as main event and relaparotomy and death as competing risks. Since randomization is stratified by study centre and level of contamination, the models will include covariates treatment group, study centre, and level of contamination.. Both analyses will be performed on the ITT set, consisting of all patients included in the study in the treatment arm they were randomized to. The global significance level is set to 5%. Using the Bonferroni-Holm adjustment, the local significance level will be 2.5% and 5% in the order of increasing p-value.<br>Missing data: Missing primary endpoint data in the primary analysis will be dealt with using competing risks and censoring. Missing SSI evaluation due to death or relaparotomy will be considered a competing risk. Missing SSI for all other reasons will be censored. Data will not be imputed for other analyses such as secondary or subgroup analyses.<br>Safety: The assessment of safety will be based on the frequency of AE/SAE other than SSI within the safety population (according to CTCAE Version 4.03), consisting of all patients randomized into the study.<br>Secondary endpoint(s): Secondary endpoints will be analyzed on the ITT set using appropriate descriptive statistics. Subgroup analyses will be performed by use of logistic regression models involving main effects and interaction effects. Any explorative statistical testing will be performed two-sided using a significance level of 5%.
**Sample size**	To be assessed for eligibility (n): approximately 5500<br>To be assigned to the trial (n): 680<br>To be analyzed (n): 680<br>The sample size was adjusted based on the changed analysis of SSI. The global significance level was set to 5% (two-sided tests). Since the PHX arm will be used twice for a comparison, the Bonferroni-Holm procedure was used to set the local alpha level for test 1 (PHX vs. no intervention) to 2.5% and for test 2 (PHX vs. saline irrigation) to 5%. If 290 patients are recruited in the PHX arm, 290 patients in the saline arm and 100 patients in the control arm (a total of 680 patients), the two Fine and Gray sub-distributional hazard models will have a power of 80% each to detect differences between the treatments.
**Trial duration subject**	Intervention: Single intraoperative intervention<br>Follow-up: max. 36 days
**Trial duration project**	First patient in to last patient out (months): 55<br>Recruitment period (months): 54<br>Duration of the entire trial (months): 61
**Participating centers**	Planned: n about 11
**Financing**	Deutsche Forschungsgemeinschaft (DFG) grant number: MU 3928/1-1

30. ABBREVIATIONS

AE	Adverse Event
ALT/ALAT	Alanine Aminotransferase
AMG	Arzneimittelgesetz
aPTT	Activated partial Thromboplastin time
ASA	American Society of Anesthesiologists
AST/ASAT	Aspartate Aminotransferase
BfArM	Bundesinstitut für Arzneimittel und Medizinprodukte
BMI	Body-Mass Index
CDC	Centre for Disease Control and Prevention
CI	Confidence Interval
Cr	Creatinine
CTCAE	Common Terminology Criteria for Adverse Events
DFG	Deutsche Forschungsgemeinschaft
DRKS	Deutsches Register Klinischer Studien
DSUR	Development Safety Update Report
eCRF	electronic Case Report Form
EDTA	Ethylene-diamineteraacetic acid
GCP	Good Clinical Practice
Glu	Glucose
ICF	Informed consent form
ICH	International Conference on Harmonization
ICMJE	International Committee of Medical Journal Editors
IMP	Investigational Medicinal Product
IMSE	Institut für Medizinische Statistik und Epidemiologie
INR	International normalized ratio
IOWI	Intraoperative wound irrigation
ISF	Investigator site file
ITT	Intention-To-Treat
K	Potassium
MeSH	Medical Subject Heading
MRI	Klinikum München rechts der Isar
MSZ	Münchner Studienzentrum
Na	Sodium
NaCl	Sodium chloride
NICE	National Institute for Health and Clinical Excellence
NNIS	National Nosocomial Infections Surveillance
PHX	Polyhexanide
PRISMA	Preferred Reporting Items for Systematic Reviews and Meta-Analyses
PT	Prothrombin time
PVP	Polyvinylpyrrolidone, Povidone
RCT	Randomized Controlled Trial
RDE	Remote Data Entry
SAE	Serious Adverse Event
SAR	Serious Adverse Reaction
SAS	Statistical analysis system
SGOT	Serum glutamic oxaloacetic transaminase
SGPT	Serum glutamic pyruvic transaminase
SMB	Safety Monitoring Board
SmPC	Summary of product characteristics
SOP	Standard operating procedure
SSI	Surgical site infection
SUSAR	Suspected Unexpected Serious Adverse events
TUM	Technical University of Munich
WHO	World Health Organization

31. INTRODUCTION

31.1 The medical problem

Postoperative surgical site infection (SSI) represents the third most common hospital infection. According to the CDC's classification [1], SSI can be subdivided into infections of the subcutaneous tissue (superficial SSI), deep soft tissues such as fascial and muscle layers (deep SSI) and infections of organs or spaces (organ/space SSI) that occur within 30 days after surgery (attachment 1). In abdominal surgery, SSI rates are especially high. Recent high-level randomized controlled trials (RCTs) with standardized SSI definitions found rates between 14.5% (BaFO trial) [2], 15.4% (PROUD trial) [3] and 25.0% (ROSSINI trial) [4] following laparotomy. Therefore, measures to prevent SSI in this field are urgently needed. Prophylactic intraoperative wound irrigation (IOWI) of the subcutaneous and deep soft tissue before skin closure with saline or antiseptic solutions hypothetically represents an easy and economical option to reduce SSI rates and is already frequently used in clinical practice, even though there are currently no definite recommendations on this practice [5]. The latest official guideline for the prevention of SSI by the World Health Organization (WHO) published in 2016, states that IOWI with saline is not efficient, but IOWI with diluted Polyvinylpyrrolidone (PVP)-iodine solutions has a potential benefit in preventing SSI, however, due to the low level of underlying evidence these recommendations are conditional and not limited to abdominal surgery [6]. In contrast, the clinical guidelines of the British National Institute for Health and Clinical Excellence (NICE) from 2008 state that IOWI's efficacy is unproven and its use should be avoided at all. However, this recommendation too, is based on a small number of unstandardized RCTs evaluating different types of surgery and irrigation solutions [7]. Antiseptic PHX-based solutions are approved for intraoperative soft-tissue wound irrigation in surgery, and have been shown to be tissue tolerable and even promote wound healing. To our knowledge prophylactic PHX wound irrigation has not yet been evaluated in RCTs in abdominal, visceral surgery [8, 9].

31.2 Evidence

Even though the literature concerning prevention of SSI is substantial, high-level evidence to guide decisions on the use of IOWI with saline or antiseptics remains scarce. Clinical trials investigating the efficacy of IOWI have been conducted mainly in the 1980-90's and their results are inconclusive and heterogeneous patient inclusion and outcome criteria were used. A few authors conducted systematic reviews and meta-analyses investigating specific irrigation solutions such as PVP-iodine or antibiotic solutions [10-13]. However, none of these reviews resulted in a definite conclusion, although they all observed a positive trend in the reduction of SSI rates through IOWI. Furthermore, more recent clinical trials have been conducted in the meantime. Therefore, we performed a large-scale meta-analysis in accordance with the Cochrane guidelines of the existing evidence on IOWI with saline, PVP-iodine or antibiotic

irrigation solutions. Pubmed/MEDLINE, EMBASE, and the Cochrane Central Register of Controlled Trials (CENTRAL) were searched in May 2013. The following search terms were used in various combinations: prevention of surgical site infection, abdominal surgery, surgical wound infection/prevention and control [MeSH Terms], wound irrigation, wound lavage, incisional surgical site infection, intra operative irrigation, intra operative lavage, antibiotic irrigation, antibiotic irrigation solutions, iodine irrigation, povidone iodine irrigation, saline irrigation, and topical anti-infective agents [MeSH Terms]. The abstract and title search was limited to clinical trials published in English or German between January 1, 1970 and May 1, 2013. In addition, all articles within the reference list of retrieved studies and reviews were hand-searched. The search was performed by two independent reviewers and followed the published protocol corresponding to the PRISMA statement and the Cochrane Handbook of systematic reviews of interventions. Prospective RCTs investigating the primary outcome of postoperative SSI after IOWI of the surgical incision after closure of the fascia or peritoneum and before skin closure were eligible for inclusion. Eligible irrigation solutions were saline, PVP-iodine, or topical antibiotics in different forms and concentrations (dry powder sprays or wound powder were also acceptable), irrespective of the closure and irrigation technique. Acceptable comparators were ‘no irrigation’ or irrigation with saline. All types of open abdominal surgeries were eligible, including visceral, gynecological, urological, or vascular procedures irrespective of the urgency of operation (elective or emergency). All trials reporting clinical SSI were included irrespective of the SSI definition used. Trials in which only one of the compared treatment arms received systemic prophylactic antibiotics were excluded, as this would have caused substantial bias. Methodological quality of individual clinical trials was assessed by examination of the allocation sequence, allocation concealment and double blinding using the Cochrane tool for assessing the risk of bias [21]. The risk of bias was graded as low, unclear, or high. In addition, the risk of publication bias was investigated by means of a funnel plot. Due to the naturally expected heterogeneity in performance of surgical procedures between different types of surgery, grade of contamination, and hence trials, random effect models with Mantel-Haenszel weights were used to estimate the average treatment effect and a corresponding 95 % CI. Forest plots were shown to illustrate treatment effects estimated for each trial and the estimated average treatment effect for all investigated subgroups. A two-sided level of significance of less than 5.0 % was considered for all tests. The results of this analysis show a risk reduction of 46 % in the treatment group (IOWI with any irrigation solution). Incidence of SSI was 9% in the irrigation group compared to 16% in the untreated group [14]. However, the majority of included trials have been published from 1970 to 1990, and the quality assessment revealed that most of them were at a high risk of bias, mainly because of insufficient data reporting and methodological flaws. Methods of sequence generation, allocation concealment, and blinding were often inadequate or not reported. In addition, interventions, follow-up times, and definitions

of SSI varied widely between studies, which might explain the large variance in overall SSI rates between 3.0 and 58.2%. Most studies used a non-standardized definition of SSI. The current internationally accepted CDC definition was not published until 1999. The funnel plot showed an asymmetry, which indicates a possible publication bias, as all included trials with a high standard error for the log odds ratio show a large benefit for the experimental group. Furthermore, PVP-I and antibiotic solutions are currently not recommended for this indication due to potential adverse side effects, tissue toxicity and the increased development of antimicrobial resistances. The only standardized RCT comparing IOWI with saline irrigation *vs.* no irrigation after open appendectomies was published in 2000 and found a reduction of SSI from 25% to 8.7% in the saline group [15]. Recently, PHX-based antiseptic solutions are successfully and widely used in orthopedic and trauma surgery. Wound irrigation with PHX showed a reduction of the SSI rate of almost 75% compared to Ringers solution in traumatic dirty contaminated soft tissue wounds [16].

31.3 The need for a trial

SSIs contribute significantly to postoperative morbidity and mortality. In Germany approximately 128,000 SSIs are reported annually [17]. Studies have shown an increase of 6-24 days in the mean length of hospital stay if SSI occurs [18]. In addition to the risk and discomfort for the patient, SSIs dramatically increase treatment costs and indirect costs such as loss of workforce or insurance payments. In Germany, postoperative SSIs account for approximately 1 million extra days of hospitalization and additional costs of around € 3 billion per year [19, 20]. Clinical guidelines and clinical practice vary largely in terms of the use of IOWI to reduce the incidence of SSI [5]. The aim of this prospective, multicenter, randomized clinical trial is to show the reduction of SSI rates by IOWI with PHX compared to saline or no irrigation. Individual patients participating in this trial have the opportunity of directly benefitting of the anticipated positive effect of PHX and/or saline irrigation, whilst no negative effects are to be expected. The results of the trial will provide evidence for definite clinical recommendations that would change current clinical guidelines and practice. A commercial interest is not expected as PHX solutions are widely available and several companies offer this product in their portfolio. The trial further does not request a certain product in order to avoid compliance conflicts, but encourages collaborators to use the available product in their respective study sites.

31.4 Summary and aims of the study

SSI is one of the most common complications following abdominal visceral surgery (14-25%) [2-4, 21] and dramatically increases length of hospital stay and costs. Hypothetically, IOWI before skin closure with saline or antiseptics might be a potential pragmatic option to reduce SSI rates. Currently, there are no official recommendations on its use and clinical practice varies largely.

Solutions containing the antiseptic agent PHX are approved for IOWI, and were shown to promote wound healing [8, 9], but have not been evaluated in RCTs in abdominal visceral surgery. Therefore, we designed a multicenter, randomized, observer-blinded clinical trial evaluating the efficacy of IOWI with PHX solution or saline before skin closure after laparotomy. Based on a meta-analysis on IOWI with various solutions, a sample-size of 540 patients was calculated for a 3-armed study design (PHX- *vs.* saline irrigation *vs.* no irrigation). The trial shall be conducted in 10 centers within the German surgical trial network CHIR-*Net*. All patients undergoing visceral surgery by laparotomy within the recruitment period of 27 months will be screened for the trial. The primary endpoint is the incidence of SSI 30 days postoperatively, according to the CDC definition (attachment 1). The results of the trial will provide evidence for definite clinical recommendations regarding the use of IOWI and influence current guidelines and provide all participating patients the opportunity of an improved treatment.

32. OUTCOME MEASURES

32.1 Rationale of outcome measures

The primary efficacy endpoint of this trial is SSI within 30 days postoperatively, according to the internationally accepted and recommended SSI definition by the CDC [1]. This endpoint has been used in previous trials and assures comparability of the results [2-4, 21]. This endpoint is further considered to be of clinical relevance as SSI increases morbidity and mortality of individual patients, direct and indirect costs and prolongs hospital stay as outlined before. The secondary endpoint of non-infectious wound complications was chosen to evaluate, if PHX irrigation has an additional positive effect on wound healing. Furthermore, secondary endpoints are morbidity and mortality within 30 days postoperatively. For safety analyses and the duration of hospital stay to evaluate the potential economical benefit.

32.2 Determination of primary and secondary measures

The primary efficacy endpoint measure of the trial is the incidence of SSI within 30 days after surgery diagnosed. Furthermore, in case of SSI, the depth of infection will be classified into one of three categories according to CDC definition (superficial, deep, organ-space, see attachment 1). In addition, the following outcome measures have been defined as secondary endpoint measures and will be determined by the unit given in parentheses: a) Duration of hospital stay (in days); b) 30-days rate of reoperation in both groups (%); c) 30-days rate of non-infectious wound complications in both groups (in %); d) 30-days rate of postoperative AE/SAE in both groups (%); e) 30-days mortality in both groups (%); (f) 30-days morbidity in both groups (%). All AE/SAEs that are surgical complications will be additionally classified according to the Clavien Dindo classification of surgical complications (attachment 2) [22].

33. FINANCING

The clinical trial is financed by a grant from the German Research Society (Deutsche Forschungsgemeinschaft; DFG), grant number: MU 3928/1-1. No co-financing by industry or other third parties applies. There is no conflict of interest for the management of the study. All participating trial sites have officially declared no conflict of interest within the eligibility evaluation of the MSZ. A commercial interest does not apply as PHX solutions are widely available and several companies offer this product in their portfolio. The trial further does not request a certain product in order to avoid compliance conflicts, but encourages collaborators to use the available product in their respective study sites.

34. RISK / BENEFIT ANALYSIS

No additional risks for study patients are anticipated, since IOWI represents a clinically established standard method. PHX 0.04% irrigation solution is approved for surgical wound irrigation of soft tissue wounds. The study will be planned, conducted and analysed according to all relevant national and international rules and regulations according to AMG [23], ICH-GCP E6 [24], and the Declaration of Helsinki, 2008 (see 27.). No specific risks are expected because IOWI is locally applied and neither application of PHX or saline will have systemic effects on the participants. Safety of PHX solutions has been demonstrated before in the marketing studies. Adverse effects may only be expected in the improbable event of accidental contamination of the respective irrigation solutions or in case of unknown hypersensitivity to PHX. The potential benefits of reduced SSIs outweigh the mentioned negligible adverse effects of PHX and saline. The subjects´ safety is ensured by regular study visits, enforcing GCP-guidelines. A subject-insurance for all trial participants is mandatory according to AMG. The informed consent process adheres to GCP-guidelines, which maximize patients´ safety and guarantee confidentiality.

35. TRIAL IMPLEMENTATION

35.1 General study design

This study is a prospective, randomized, controlled, observer and patient-blinded, multicenter, surgical trial with three parallel comparison groups. Pre-screening of potential patients (evaluation of inclusion and exclusion criteria) is possible up to 14 days prior to the planned procedure. Patients can be included in the trial if inclusion and exclusion criteria apply and

written informed consent has been provided. In case of emergency procedures inclusion is possible on the same day as the procedure, if the patient is able to understand and provide written informed consent and has had a reasonable amount of time to think about the decision (see 12.3). Included patients are randomized to no epifascial wound irrigation, epifascial wound irrigation with saline 0.9% or epifascial wound irrigation with PHX 0.04% solution. Screened but excluded patients will be documented in a screening log.

35.2 Trial duration

The estimated overall length of the study is 42 months, which assembles as follows:

IV. Trial preparation: ~ 6 months

V. Execution of study: First patient in to last patient out: ~ 55 months

1. Begin of study: 3rd quarter, 2017
2. End of study: 2nd quarter, 2022 (Completion of the last visit for the last patient represents the end of study)
3. Recruitment period: ~ 54 months
4. Duration of treatment per patient:
   a) Group with intervention 1: Surgery according to institutional standard, followed by one-time wound irrigation with PHX 0.04% solution.
   b) Group with intervention 2: Surgery according to institutional standard, followed by one-time wound irrigation with saline 0.9% solution.
   c) Control group: Surgery according to institutional standard, followed by no wound irrigation.
5. Duration of follow-up per patient: 30 days (+6 days at the latest)

For all three groups, documentation of the primary and secondary endpoints up to postoperative day 30 is warranted.

VI. Analysis, publication ~ 7 months

Graph 1: IOWISI intervention scheme / trial flow

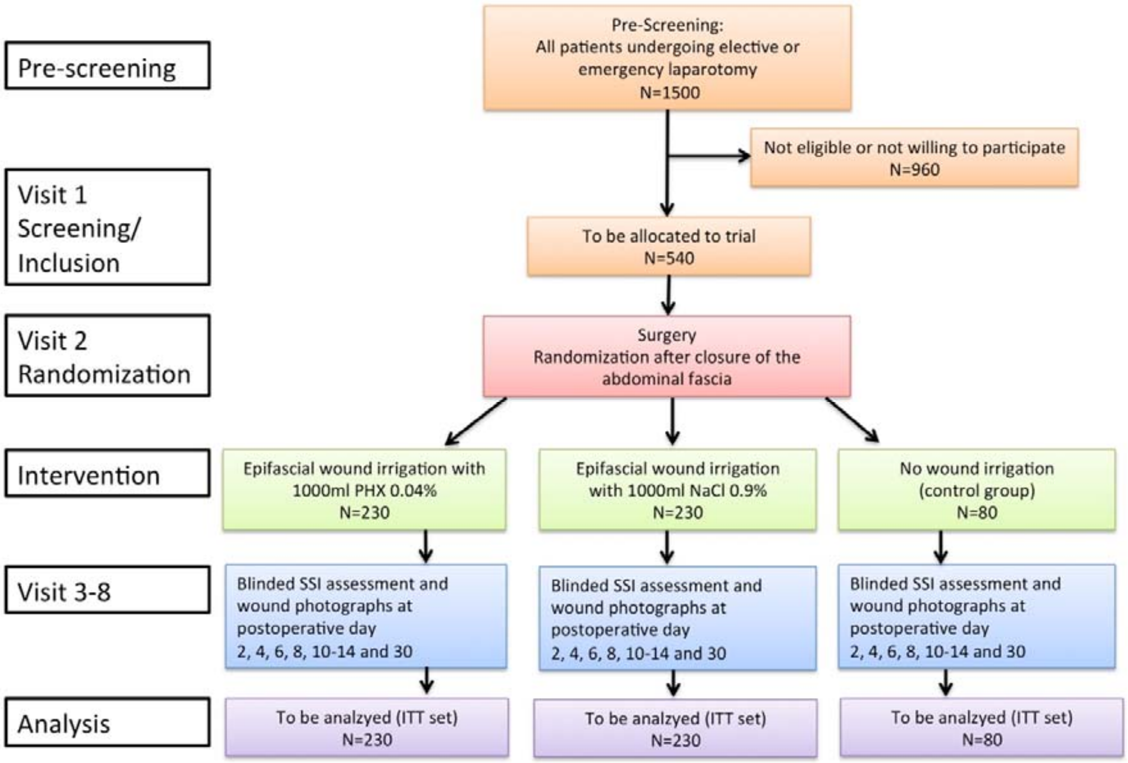

Graph 2: IOWISI study visits (according to SPIRIT statement 2013 [25])

	STUDY PERIOD							
	INCLU.	RAND.	POST-ALLOCATION					CLOSE-OUT
**STUDY VISIT**	**1**	**2**	**3**	**4**	**5**	**6**	**7**	**8**
**TIMEPOINT**	- 1-3 days*	Surgery (day 0)	day 2	day 4	day 6	day 8	day 10-14	day 30[§]
**INCLUSION**								
Informed consent	X							
Inclusion and exclusion criteria	X							
**RANDOMIZATION**		X						
**INTERVENTIONS**								
Intervention 1(IOWI with 1000ml PHX 0.04%)		X						
Intervention 2(IOWI with 1000ml NaCl 0.9%)		X						
Control group (no IOWI)		X						
**ASSESSMENTS**								
Demographical data	X							
Medical history	X							
Concurrent medication	X							
Physical examination	X							
NNSI Risk score	X							
Pregnancy test**	X**							
Blood sample***	X			X****				
Type of operation		X						
Duration of operation		X						
Level of contamination		X						
Type and length of incision		X						
Wound closure technique and suture material		X						
Creation of an enterostomy		X						
Administration and timing of antibiotic prophylaxis		X						
Intraoperative use of wound edge protectors		X						
Changing of gloves during operation		X						
Postoperative medication with effect on wound healing			X	X	X	X	X	X
Documentation of SSI			X	X	X	X	X	X
Documentation of other wound complications			X	X	X	X	X	X
Wound swab for microbiology[+]			X[+]	X[+]	X[+]	X[+]	X[+]	X[+]
Photograph of the wound			X	X	X	X	X	X
Documentation of re-operation			X	X	X	X	X	X
Documentation of AE/SAE		X	X	X	X	X	X	X
Duration of hospital stay								X

* In case of emergency surgery enrolment is possible on the same day as the procedure
**For women of child-bearing potential only (serum or urine)
***Includes hemoglobin, hematocrit, platelets and white blood cell count, Na, K, Cr, Glu (non-fasting), AST/ASAT (SGOT), ALT/ALAT (SGPT), Bilirubin, Uric acid, Prothrombin time (PT), activated partial thromboplastin time (aPTT), international normalized ratio (INR) according to local in-house standards
****Between post-OP day 4-8 (visit 4-6)
[+]In case of SSI a swab will be taken from the wound or wound secretion for microbiological differentiation and testing of resistance to antibiotics according to local in-house standards

§ Visit window +6 days. If the patient is unable to attend visit 8 due to postoperative treatment in a rehabilitation facility or other medical reasons, a standardized protocol for evaluation and documentation of the wound will be sent to and filled out by the treating physician.

36. JUSTIFICATION OF DESIGN ASPECTS

36.1 Study design

This trial is a prospective, randomized, controlled, observer and patient-blinded, multicenter, surgical trial according to German drug law (AMG) phase IIIb with three parallel comparison groups. Reduction of SSI (according to CDC criteria) by IOWI after abdominal surgery is postulated. The IOWISI trial will be conducted in approximately 10 surgical departments (university and community hospitals), all of which are members of the trial network (CHIR-*Net*) of the German Surgical Society (*Deutsche Gesellschaft für Chirurgie*) and have experience in previous multicenter RCTs. Feasibility evaluation of all participating centers was done according to the SOPs of MSZ. All of the study personnel involved in the trial require GCP training and will be specifically instructed in all trial-specific procedures before initiation of the trial. According to AMG, the investigator requires 2 years' experience in drug trials. The leading surgeon of the operating team will perform the interventions since they represent standard techniques. All participating surgeons will be instructed and authorized by the investigator, prior to the first trial procedure.

36.2 Control and comparators

The WHO published the latest clinical guideline addressing the topic of IOWI in surgery in 2016. The consensus is that there is not sufficient evidence to support the use of IOWI with saline, diluted PVP-solutions should be considered and antibiotic solutions avoided. However, the underlying RCTs included all types of surgery (*i.e.* neuro-, orthopedic surgery.) and are of low level of evidence [6]. The guideline of the British National Institute of Clinical Excellence (NICE) from 2008 [7] states that, due to the lack of evidence any IOWI should be avoided. However, in clinical practice this advice is mostly not being followed. Most hospitals do not have standard protocols but leave the decision to irrigate or not to irrigate the wound up to the surgeon. Given these circumstances it is acceptable to recruit a control group receiving no intervention. So far, no gold standard was determined within RCTs in abdominal surgery. Therefore, the trial proposes an irrigation procedure on the best available evidence, which is either irrigation with PHX-solution or saline or no irrigation. PHX and saline solutions are widely used in clinical practice, but efficacy trials are not available momentarily. As PHX solution is a market-approved drug, safety is ensured and the trial subjects are not exposed to specific risks.

36.3 Additional treatments

No additional treatments will be performed within the trial. Antibiotic treatment 5 days prior to surgery is an exclusion criterion. Pre-operative antibiotic treatment due to septic peritonitis (dirty / contaminated wounds) after admission to the hospital is allowed, but has to be recorded in the CRF. Application of routine intraoperative single shot antibiotic prophylaxis will be recorded in the CRF (type and dose of antibiotics). The application of abdominal wall protectors is recommended for contaminated procedures and has to be recorded in the CRF. A change of gloves ahead of wound closure is recommended for contaminated procedures and has to be recorded in the CRF. If indicated for medical reasons, all kind of medication is permitted during the trial. Postoperative medication with adverse effects on wound healing (*e.g.* corticoids and other immunosuppressive agents) will be recorded in the CRF. Any operative and / or interventional revision of the wound will be documented as AE/ SAE and classified after Clavien Dindo.

36.4 Blinding

The blinding procedure is restricted to participating patients, outcome assessors and the trial statistician. Blinding of the surgical team that performs the intervention is impossible because the control arm does not receive any wound irrigation. A member of the local study team, who will not take part in postoperative patient visits, performs randomization after confirmed closure of the abdominal fascia. A central online randomization tool of the MSZ (RANDOBASE) will effectuate randomization. After informing the surgical team of the result, the member of the study team has to print out, date and sign the randomization sheets. Subsequently, the randomization sheets have to be stored away from the patient records, trial documents and ISF to ensure blinding of the rest of the local study team.

Postoperatively, a GCP-trained investigator of the local study group, who is unaware of the patient's intraoperative treatment, will clinically assess the primary endpoint (SSI) on 6 study visits.

In addition, standardized photographs of the wound will be taken at each visit and uploaded to a central database. Independent, blinded outcome-assessors of spatially separated centers participating in the trial will assess those pseudonymized wound photographs in the database online. These online outcome-assessors receive training in rating of wounds according to the CDC classification of SSIs, which will be documented in a separate training log. These independent outcome-assessors will only access the photo-database for evaluation of SSI and will not be aware of the randomization results or any other patient data. All treatment-specific data are documented in a separate, undisclosed file. Wound photographs from all trial sites will

be assessed by outcome assessors of the coordinating study site TUM in Munich. Photographs from the Munich TUM study site will be assessed in the study site Munich LMU.

36.5 Exclusion of participants after initial inclusion

Participants of the study can withdraw their consent to take part at any time without declaration of reasons. All hitherto collected data are subject to analysis. The coordinating investigator or the investigator may exclude patients from the study, if patients' safety is at risk or if there is insufficient compliance of the patient. In order to generate a meaningful database, excluded patients can be replaced by recruitment of new patients. If a patient does not receive PHX or saline irrigation of the wound, this does not automatically lead to exclusion of the study.

37. INCLUSION- AND EXCLUSION CRITERIA

37.1 Inclusion criteria

- Clean-contaminated, contaminated or dirty surgery according to CDC classification (attachment 3);
- Abdominal surgery by midline or transverse laparotomy; elective and emergency procedures;
- Age ≥ 18 years;
- American Society of Anesthesiologists (ASA) score ≤ 3 (attachment 4);
- Ability to understand the nature and extent of the trial and to give written informed consent

37.2 Exclusion criteria

- Pregnancy or breast feeding;
- Known hypersensitivity/allergy to PHX;
- Inability to give/understand informed consent;
- Critical medical condition of emergency patients, precluding informed consent or sufficient time to reflect on the decision to participate in the trial;
- ASA > 3;
- Inability to attend follow-up visits;
- Clean procedures according to the CDC classification or surgery without opening of the abdominal cavity;
- Laparoscopic surgery;
- Revision-surgery (previous abdominal surgery within the last 30 days);
- Planned re-laparotomy within 30 days;
- Severe immunosuppression;

- Concurrent abdominal wall infections;
- Pre-operative systemic antibiotic therapy within 5 days prior to surgery (except emergency pre-operative antibiotic treatment due to septic peritonitis after admission to the hospital);
- Participation in another clinical trial that interferes with the primary or secondary outcomes of this trial.

37.3 Explanation of inclusion and exclusion criteria

To enhance generalizability and representativeness, all patients undergoing elective and emergency laparotomy (transverse or midline) for visceral surgery will be screened for this trial. However, only clean-contaminated, contaminated or dirty (class II-IV), open abdominal surgery, according to the CDC classification [1] will be eligible, since in clean (class I) procedures the risk of SSI is low. Laparoscopic surgery as well as surgery without opening of the abdominal cavity or revision surgery (previous abdominal surgery within the last 30 days or planned re-laparotomy within the next 30 days of surgery) will be excluded, since these types of procedures are not comparable in terms of SSI risk.

Pre-operative antibiotic therapy within 5 days prior to surgery was chosen to be an exclusion criterion to avoid bias of the results, since this might lead to a lower individual risk of infection. However, this does not apply to patients that receive pre-operative antibiotics after admission to the hospital in an emergency situation of septic peritonitis. Furthermore, this does not include standard intraoperative single shot antibiotic prophylaxis.

Patients have to be ≥ 18 years of age and able to understand and give written informed consent. Any patient in a very bad general medical condition (ASA > 3) will be excluded to avoid too many patient-related confounders. Emergency patients in a critical medical condition that does not allow them to fully understand and provide informed consent or does not leave them sufficient time to reflect on the decision to participate in the trial will not be included. Furthermore, patients have to be able to attend follow-up visits.

Patients with severe immunosuppression (*e.g.* after: organ or bone marrow transplantation, concurrent steroid treatment with >10 mg prednisone daily or an equivalent dose of any other steroid), concurrent infliximab treatment or treatment with an equivalent immunosuppressive substance, chemotherapy within the last 2 weeks prior to trial intervention) or patients with severe pre-operative neutropenia (≤ 0.5×10^9/L) or liver cirrhosis Child-Pugh B/C will not be included. Pregnant or breast feeding women, as well as patients with a known hypersensitivity/allergy to PHX will not be included in the trial either.

Patients that participate in other clinical trials that could interfere with the primary (SSI) or secondary outcomes of the IOWISI trial will be excluded.

38. FREQUENCY AND SCOPE OF TRIAL VISITS

Graph 1 and 2 reflect the intervention scheme, trial flow, and visits for the IOWISI trial. Visits are the same for all participants of the study, regardless the treatment group.

38.1 Recruitment and screening

Only surgical departments with adequate patient numbers, providing a written commitment on their recruitment capacity were included in the trial to reach the target sample size. The recruitment period is set to 54 months (first patient in to last patient out 55 months). In case of elective procedures, pre-screening (this is just a pre-selection of eligible patients within the study team) of patients can be performed up to 14 days prior to the scheduled surgical procedure. Screening and inclusion of patients will be performed not earlier than 3 days and not later than on the day before the planned surgical procedure, to ensure the patient has enough time to consider the decision to participate. In case of emergencies, screening and inclusion can take place on the day of admission to the hospital, which is usually the same day as surgery. All screened patients are documented in a screening log. If patients do not wish to participate in the study, reasons are documented accordingly. If patients fit inclusion/exclusion criteria and agree to participate, they will need to give written informed consent to the local GCP-trained investigator, after adequate time for consideration in order to participate in the study (representing visit 1). Therefore, at the screening visit, a detailed description of the study and further instructions are discussed with the patient, including methods of wound irrigation, risk-benefit-ratio, and follow up schedule.

38.2 Visit 1 (Inclusion)

After the local investigator has reviewed the inclusion and exclusion criteria again and having received written consent by a patient, demographical data / medical history (date of birth [mm/yyyy], gender, body height, body weight, BMI, ASA, medical history, concurrent medication, history of SSI, history of radio/chemotherapy, diabetes, smoking, alcohol consumption, medication, duration of pre-operative hospital stay), diagnosis and the NNIS Risk score for determining the intrinsic risk of SSI (attachment 5) will be documented according to the eCRF. The investigator will perform a physical exam (blood pressure, heart frequency, condition of the planned abdominal surgical incision area, clinical relevant findings [normal or abnormal (please specify), respiratory system, cardiovascular system, liver, kidney, neurological or other free text] and take a blood sample (EDTA, Serum, and Citrate). Measurements of the blood sample are:

- Hemoglobin
- Hematocrit

- Platelets
- White blood cell count
- Sodium
- Potassium
- Creatinine
- Non-fasting glucose
- AST/ASAT
- ALT/ALAT
- Bilirubin
- Uric acid
- Prothrombin time (PT)
- Activated partial thromboplastin time (aPTT)
- International normalized ratio (INR)

In case of women of child-bearing potential, a pregnancy test will be performed additionally (serum or urine [negative/positive/not performed with specification of reason as free text]).

38.3 Visit 2 (Surgery/Randomization)

Documented parameters of the surgical procedure include the urgency (emergency/elective), type of surgical procedure (colorectal and/or small bowel and/or hepato-biliary and/or pancreatic and/or splenectomy and/or gastric and/or esophageal and/or nephrectomy and/or urogenital tract and/or others (freetext)) the duration of surgery (incision until complete skin closure, minutes), the level of contamination according to CDC classification (class II-IV; see attachment 3), the intraoperative use of wound edge protectors (yes/no), and prophylactic changing of gloves during of the operation (yes/no), type (transverse/midline) and length (cm) of the incision, creation of an enterostomy (yes/no), the wound closure technique (subcutaneous sutures (yes/no), stapler/suture, if suture: continuous/single) and used suture material, the administration (yes/no) and timing (>1h/≤1h prior to incision) of antibiotic prophylaxis. If the operating surgeon decides that incomplete closure of the wound and/or any other wound related procedure after the study intervention (*e.g.* negative pressure treatment) is necessary for the benefit of the patient, the patient will have to be excluded from the trial.

Randomization (see section 24.) will take place at the end of surgery, after closure of the abdominal fascia, when the level of contamination is definitely determined by the surgeon. A designated member of the local study team (who will not perform postoperative study visits) will perform randomization instantly by using the online tool of the MSZ (RANDOBASE) and inform the surgeon of the result and according treatment. Date of randomization (mm:hh, dd/mm/yyy), successful randomization (yes/no), and the result of the randomization process are

documented (printout). Subsequently, the randomization sheets have to be stored away from the patients file to ensure blinding.

Study treatment according to randomization:

- Wound irrigation with PHX 0,04% 1000ml
- Wound irrigation with NaCl 0,9% 1000ml
- No wound irrigation

Furthermore, any AE or SAE is documented during this visit.

38.4 Visit 3 to 8 (Post-op days 2, 4, 6, 8, 10-14, and 30-36)

Postoperatively, there will be 6 trial visits where an independent, blinded outcome assessor trained in the diagnosis and classification of SSI according to CDC definitions will examine wounds (SSI superficial or deep or organ/space, see attachment 1). In addition, pseudonymized, electronic pictures of the wound will be uploaded to a centralized database for independent and blinded evaluation (see 11.4). The assessors will not be aware of the study procedure or other details of the examined wound photograph. Postoperative medication with adverse effects on wound healing (*e.g.* corticoids and other immunosuppressive agents) will be documented in the eCRF.

In case of SSI, microbiological swabs will be taken from the wound secretion for microbiological differentiation and testing of resistance to antibiotics according to in-house standards by each local institution. Other wound complications like seroma, hematoma, delayed healing or necrosis will be documented as secondary endpoint. Any surgical complication, including SSI, will be reported as AE/SAE and the Clavien Dindo classification (attachment 2) will be applied to specify the severity and consequent treatment. Furthermore, the rate of re-operations, mortality and occurrence of any AE or SAE will be documented (see 16). Additionally, the duration of the hospital stay (from admission to discharge or day of the visit, in days) will be documented on visit 8 (post-op day 30-36). To promote complete follow-up, a visit window of 6 additional days was implemented. In addition, patients can be recompensed for any travel expenses needed to attend study visit 8. If however, the patient is unable to attend visit 8 due to postoperative treatment in a rehabilitation facility or other medical reasons, a standardized protocol for evaluation and documentation of the wound (incl. wound photograph) will be sent to and filled out by the treating physician.

Between post-op day 4 and 8 (visit 4, 5 or 6) one study-specific, post-operative blood sample will be taken, and the same measurements as upon visit 1 will be analyzed according to local clinical routine:

- Hemoglobin
- Hematocrit
- Platelets
- White blood cell count
- Sodium
- Potassium
- Creatinine
- Non-fasting glucose
- AST/ASAT
- ALT/ALAT
- Bilirubin
- Uric acid
- Prothrombin time (PT)
- Activated partial thromboplastin time (aPTT)
- International normalized ratio (INR)

39. DOSE, MODE AND SCHEME OF INTERVENTION

After closure of the abdominal fascia, patients will be randomized stratified by level of contamination of the operation. In the experimental group 1, the subcutaneous soft tissue will be irrigated with 1000 ml of a 0.04% PHX solution, which is the recommended concentration for surgical wound irrigation according to the SMPC. PHX solutions (0.04%) are approved for this indication in Germany. The wound shall be carefully rinsed throughout with the irrigation solution and the excess removed with suction. Debris and blood clots should be removed from the wound using irrigation/suction. The wound shall not be rubbed dry with abdominal cloths, but left moistened with the irrigation solution to ensure sufficient contact time for PHX to have the desired antiseptic effect. After irrigation with PHX the wound shall not be irrigated with saline or any other solution again. Since PHX is a cation-active substance, it is not compatible with anionic organic substances (*e.g.* lactate). Furthermore, the combination of PHX with PVP-I products should be avoided.

In the experimental group 2, the same intervention will be performed using 1000ml of isotonic saline solution (NaCl 0.9%).

The irrigation volume of 1000ml was chosen to be sure that even large laparotomy wounds would be sufficiently irrigated. This was determined by senior surgeons´ clinical experience, since so far no recommendations for the optimal volume of surgical irrigation exist. After

irrigation of the wound, the skin closure will be performed according to local standards, without any further wound-related procedure.

In the control group, wounds will not be surgically irrigated, as is currently recommended in the NICE guideline. PHX solutions or saline are to be purchased, stored, and distributed according to the respective trial centers standard operating procedures. Trade name, dosage, batch and dispensed amount will be documented on a separate form.

40. PATIENT, STUDY AND SITE DISCONTINUATION

40.1 Patient discontinuation

Patients have the right to voluntarily withdraw from the study at any time for reason. In addition, the investigator has the right to withdraw a patient from the study at any time. Reasons for withdrawal from the study may include but are not limited to the following:

- Patient withdrawal of consent at any time;
- Any medical condition that the investigator or sponsor determines may jeopardize the patient's safety if he or she continues in the study;
- If it is discovered that a study subject is pregnant or may have been pregnant at the time of intervention (see point 16.9);
- Investigator or sponsor determines it is in the best interest of the patient to discontinue the study.

Every effort should be made to obtain information on patients who withdraw from the study. The primary reason for withdrawal from the study should be documented on the appropriate eCRF. However, patients will not be followed for any reason after consent has been withdrawn. Patients who withdraw from the study will not be replaced.

40.2 Study and site discontinuation

The **sponsor** has the right to terminate this study at any time. Reasons for terminating the study may include but are not limited to the following:

- The incidence or severity of AEs in this or other studies indicates a potential health hazard to patients;
- Unsatisfactory patient enrolment;
- The continuation of study is unethical or it has been proven that the therapy has a clearly negative influence;
- Unforeseen complications arise that no longer justify a continuation of the study;

The **sponsor** will notify the investigator of a decision to discontinue the study. The sponsor has the right to **close a site** at any time.

Reasons for closing a site may include, but are not limited to, the following:

- Excessively slow recruitment;
- Poor protocol adherence;
- Inaccurate or incomplete data recording;
- Non-compliance with the ICH-GCP guideline;
- No study activity (*i.e.* all patients have completed and all obligations have been fulfilled);

The **investigator** can discontinue the clinical study at his site at any time if he no longer considers the continuation of the study, for example if there are ethical and/or medical concerns.

41. ADVERSE EVENTS (AES)

41.1 Definition adverse event (AE)

An AE is any untoward medical occurrence in a patient or in a clinical investigation subject administered a pharmaceutical product, which does not necessarily have a causal relationship with this treatment. An AE can therefore be any unfavourable and unintended sign (including an abnormal laboratory finding), symptom or disease temporally associated with the use of a medicinal product, whether or not related to the treatment. Any AE has to be documented in the eCRF on the respective “Adverse Event Report Form”.

41.2 Specific definitions of AEs in the IOWISI trial

The obligation to document any AE in the study, starts with the randomization and ends with completion of the last study visit. AE/SAEs are documented according to the standard grading on the AE/SAE reporting forms. Surgical site infections (primary endpoint) and all other local wound complications (secondary endpoint) will be documented as AE/SAE. In addition, their severity and the consequent treatment will be documented according to the Clavien Dindo classification (attachment 2). All laboratory values or events that will be assessed as “clinically significant” in the eCRF have to be documented as an AE. The responsible medical investigator will judge the clinical significance in the context of the postoperative course after laparotomy and the correspondent laboratory values before intervention.

41.3 Serious adverse events (SAE) and other definitions

Serious adverse events (SAEs)

A SAE is defined as any clinical event that at any time during the study participation:

- Results in death;

- Is life-threatening (the term life-threatening refers to an event in which the subject was at risk of death at the time of the event and not to an event which hypothetically might have caused death if it was more severe);
- Requires subject hospitalization or prolongation of existing hospitalization;
- Results in persistent or significant disability/ incapacity.
- Results in a congenital anomaly/birth defect or
- Is rated as another significant event or condition by the investigator

Any SAE has to be reported to the MSZ immediately after becoming aware of the event (see chapter 16.7).

Suspected Unexpected Serious Adverse Reaction (SUSAR)

Serious AEs that are both suspected, *i.e.* possibly related to the investigational medicinal product (IMP) and ‘unexpected’, *i.e.* the nature and/ or severity of which is not consistent with the applicable product information, are to be classified as Suspected Unexpected Serious Adverse Reactions (SUSARs). If the second assessor classifies the SAR as ‘suspected’ (the relationship to the IMP is “related”, “probable” or “possible”) and unexpected, it will be categorized as a SUSAR. All SUSARs are subject to an expedited reporting to the responsible ethics committee(s), the competent federal authority (BfArM) and to all participating investigators (see 16.7). Furthermore, a report on all observed SAEs / SARs / SUSARs will be submitted once a year in the DSUR (Development Safety Update Report) format.

Period of observation and documentation

In this trial, all AEs that occur between the randomization (during surgery) and the last study visit or premature study termination will be documented on the pages provided in the eCRF. AEs must also be documented in the subject’s medical records. All subjects who have AEs, whether considered associated with the use of the trial medication or not, must be monitored to determine the outcome. The clinical course of the AE will be followed up until resolution or normalization of changed laboratory parameters or until it has changed to a stable condition.

41.4 Evaluation of the severity

The grading of AEs in this trial will be carried out on the basis of the 5-grade scale defined in the CTCAE V4.03:

Grade 1: Mild AE
Grade 2: Moderate AE
Grade 3: Severe AE
Grade 4: Life-threatening AE or AE causing disablement
Grade 5: Death related to AE

The grading of all AEs listed in the CTCAE v4.03 will be based on the information contained therein. The grading of all other AEs, i.e. those which are not listed in the CTCAE v4.03 will be performed by a responsible investigator, based on definitions given above. In addition, surgical complications will be evaluated according to the Clavien Dindo classification.

41.5 Evaluation of the causal relationship

Investigators will estimate the causal relationship between the AE/SAE and the treatment. When estimating the causality the investigator may draw on known biophysical parameters, incorporate previous knowledge on the AE profile of the investigational product and possible simultaneously factor in the efficacy against other substances and the concomitant diagnoses of the patient. The investigator will categorize each AE that occurred after administration of the IMP regarding the coherency with the administration of the IMP as:

- **Related:** There is a reasonable possibility that the event may have been caused by the IMP. A certain event has a strong temporal relationship and an alternative cause is unlikely.
- **Probable:** An AE that has a reasonable possibility that the event is likely to have been caused by the IMP. The AE has a timely relationship and follows a known pattern of response, but a potential alternative cause may be present.
- **Possibility:** An AE that has a reasonable possibility that the event may have been caused by the IMP. The AE has a timely relationship to the IMP; however, the pattern of response is untypical, and an alternative cause seems more likely, or there is significant uncertainty about the cause of the event.
- **Unlikely:** Only a remote connection exists between the IMP and the reported AE. Other conditions including concurrent illness, progression or expression of the disease state or reaction of the concomitant medication appear to explain the reported AE.
- **Not related:** An AE that does not follow a reasonable temporal sequence related to the IMP and is likely to have been produced by the subject's clinical state, other modes of therapy or other known aetiology.

41.6 Outcome of AEs

The outcome of an AE at the time of the last observation will be classified as:

- **Recovered/ Resolved**: All signs and symptoms of an AE disappeared without any sequels at the time of the last interrogation.
- **Recovering/ Resolving:** The intensity of signs and symptoms has been diminishing and/ or their clinical pattern has been changing up to the time of the last interrogation in a way typical for its resolution. Further follow-up is possibly needed.
- **Not recovered/ Not resolved**: Signs and symptoms of an AE are mostly unchanged at the time of the last interrogation. Further follow-up is possibly needed.

- **Recovered/ Resolved with sequels:** The patient recovered with sequels from the AE / the AE resolved with sequels, *i.e.* the patient suffers from late complications or damage resulting from the AE.
- **Fatal:** An AE resulting in death. If there are more than one AE only the AE leading to death (possibly, related) will be characterized as ‘fatal‘.
- **Unknown:** The outcome is unknown or implausible and the information cannot be supplemented or verified.

41.7 Reporting of serious adverse events (SAEs)

Primary reporting of SAEs

All SAEs must be reported immediately, by fax (number 089/4140-6480) by the investigator to the responsible officer at the MSZ using the designated form.

Münchner Studienzentrum
SAE-Reporting
Ismaninger Straße 22
81675 München
Tel.: +49/89/4140-6477
Fax: +49/89/4140-6480 or Email: sae-msz@mri.tum.de

Reporting should be immediately after the investigator becomes aware of the event.

The initial report must be as complete as possible including details of the current illness and SAE and an assessment of the causal relationship between the event and the trial medication.

Second assessment of SAEs

All SAEs will be subject to a second assessment by a designated person. This person is elected by the sponsor and will be independent from the sponsor and the reporting investigator. The second assessor will fill out a ‘Second Assessment Form’ for each SAE. The ‘Second Assessment Form’ will contain the following information:

II) Assessment of seriousness of the event (investigator and second assessor)
II) Assessment of relationship between SAE and IMP (investigator and second assessor)
III) Assessment of expectedness of SAE, derived from IMP (second assessor)
IV) A statement if the benefit/ risk assessment for the trial did change as a result of SAE (second assessor)

The responsible safety officer of the MSZ will carry out the expedited reporting. Only SUSARs/ SAEs occurring after administration of IMPs will undergo expedited reporting.

41.8 Expedited reporting

Pursuant to the German and applicable EU laws and regulations, the ethics committee and health authorities will be informed of all suspected SUSARs and all SAEs resulting in death or being live-threatening occurring during the trial. Both institutions and all participating investigators will be informed in case the risk/ benefit assessment did change or any others new and significant hazards for subjects' safety or welfare occur. The sponsor has to ensure that all relevant information about a SUSAR, which occurs during the course of a clinical trial and is fatal or life threatening is reported as soon as possible and not later than seven days after the sponsor was first aware of the reaction. Any additional relevant information should be sent within eight days of the report. A SUSAR, which is not fatal, or life threatening has to be reported as soon as possible and in any event not longer than 15 days after the sponsor was first aware of the reaction.

41.9 Pregnancy

If, following initiation of the investigational product, it is subsequently discovered that a study subject is pregnant or may have been pregnant at the time of investigational product exposure, the investigator must immediately notify the sponsor of this event via the "Report on the drug exposure during pregnancy" and in accordance with SAE reporting procedures. The patient will be withdrawn from the study. Follow-up information regarding the course of the pregnancy, including perinatal and neonatal outcome and, where applicable, offspring information must be reported on a "Report on the pregnancy outcome during drug exposure". Any pregnancy occurring in a female partner of a male study participant the investigator becomes aware of should be reported to the sponsor. Information on this pregnancy may also be collected on the pregnancy reporting forms.

42. SAFETY MONITORING BOARD (SMB)

An independent Safety Monitoring Board (SMB according to the Guidance E3, ICH note for Guidance E6, ICH note for Guidance E9, Directive 2001/20EC "relating to the implementation of good clinical practice in the conduct of clinical trials on medicinal products for human use) is a group of experts external to the study that addresses the patient's safety and performs risk / benefit assessments. According to its operating procedures the SMB reviews accumulating safety data from ongoing trials to fulfill the safety monitoring. The rules of the SMB are deposited in the SMB Charta, (SOP_MSZ_AE04-H-A01_V02). The aim of this Charta is to define the composition, responsibilities, purpose and timing of meetings, details of the operation, including documentation and reporting and specifying the procedures to ensure confidentiality and appropriate communication of the SMB.

43. ENSURING DATA QUALITY

43.1 Documentation

All raw data such as patient records are declared as source documents. It must be ensured that they are available during routine monitoring visits. Apart from that the investigator of each site must maintain a separate patient identification list. The patient identification list will be maintained at the site separate from the documentation. The eCRF covers all the important forms, sorted according to visits. If a patient withdraws from the study, the reason must be recorded on the eCRF.

Data collection

The documentation of the study data in adherence to the GCP-guidelines and the clinical trial protocol is the responsibility of the investigator. Original data (source documents) remain in hospital medical record and information on the eCRF must be traceable and consistent with the original data. Source documents are *e.g.* laboratory results, photography, skin biopsy histology description and quality of life questionnaire, EASI, Pruritus VAS, TSQM. Original written informed consent signed by the patient is kept by the investigator and a signed copy will be given to the patient. No information in source documents about the identity of the patients will be disclosed. All data collected in this study must be entered in an eCRF which has to be completed by the investigator or authorized trial personnel and signed by the investigator. This also applies for those patients who do not complete the study. If a patient withdraws from the study, the reason must be recorded on the eCRF. The investigator is responsible for ensuring the accuracy, completeness, and timeliness of all data reported to the sponsor in the eCRFs and in all required reports.

Database management

Data are administered and processed by data management of the MSZ with the support of a study database (eCRF) according to the SOPs of the MSZ. A description of the study specific processes is given in the Data Management Plan that details the key planning and control elements for the data management component of the study.

The evaluation of the data takes place by programmed validity- and consistency checks. In addition a manual/visual evaluation of plausibility is performed in accordance to the requirements of GCP. Queries may occur, which will be visualized on the study database. The investigator has to resolve all data discrepancies in the study database. After entry of all collected data and clarification of all queries, the database will be closed at the completion of the study. The database closure has to be documented. Data and results electronically recorded will be archived according to legal guidelines at least 10 years after study termination.

43.2 Audits and inspections

As part of quality assurance according to GCP, the sponsor and the competent health authorities have the right to audit/inspect the study sites and any other institutions involved in the trial. The aim of an audit/inspection is to verify the validity, accuracy and completeness of data, to establish the credibility of the clinical trial, and to check whether the trial subject's rights and trial subject safety are being maintained. The sponsor may assign these activities to persons otherwise not involved in the trial (auditors). These persons as well as inspectors are allowed to access all trial documentation (especially the trial protocol, eCRFs, trial subjects' medical records, drug accountability documentation, and trial-related correspondence).

The sponsor and all investigators of the participating study sites undertake to support auditors and inspections by the competent authorities at all times and to allow the persons charged with these duties access to the necessary original documentation. All persons conducting audits undertake to keep all trial subject data and other trial data confidential.

After each external audit the investigator receives an audit confirmation from the responsible auditor. This confirmation has to be stored in the ISF in order to provide access to it in case of an inspection by the competent authorities. The audit report is provided to the sponsor for control.

43.3 Monitoring

Monitoring activities are performed to ensure that the trial is conducted in accordance with the trial protocol, the principles of GCP and local legislation. A monitoring manual describing the scope of the monitoring activities in detail will be prepared.

The responsible monitor will contact the investigator and will be allowed, on request, to inspect the various records of the trial (eCRF and other pertinent data) provided that patient confidentiality is maintained in accord with local requirements. The monitor should have access to patient records, any information needed to verify the entries in the eCRF and all necessary information and essential study documents. The investigator agrees to cooperate with the monitor to ensure that any problems detected in the course of these monitoring visits are resolved. A monitoring visit report is prepared for each visit describing the progress of the clinical trial and all identified problems.

43.4 Archiving

At the end of the clinical study all study-relevant data must be archived as required by law and when indicated in addition according to the Clinical Trial Agreement. All documentation forms, ICFs and other essential study documents must be retained as required by law. Patient ID lists

and patient files are retained in the respective study sites separately. The ICFs are kept in with the study documents.

44. ETHICAL AND REGULATORY ASPECTS

44.1 Sponsor‘s and investigator’s responsibilities

This study is conducted in compliance with all applicable laws and regulations and also the Declaration of Helsinki. **The sponsor has the overall responsibility for the ethical and scientific conduct of the study. All participating investigators agree to adhere to the instructions and procedures described in the study protocol and thereby to adhere to the principles of GCP that it conforms to.**

The responsible ethics committee of TUM and health authority (BfArM) will review the final study documents. The ethics committee's and BfArM’s decision concerning the conduct of the study will be communicated in written form to the sponsor. The sponsor will assure submission of required progress reports, annual safety reports and substantial amendments for approval to the ethics committee and BfArM. Before initiating the study, the sponsor must submit any required amendments to BfArM for review and acceptance to begin the trial according to § 42 AMG. Furthermore, the sponsor has to inform the ethics committee and BfArM within 90 days about completion of the trial and provide a brief report of its outcome 1 year after completion of the trial. Results of the study will be reported following ICH-GCP-E6 and published according to the CONSORT statement.

44.2 Independent ethics committees and health authorities

Prior to the start of this study, the protocol and other required documents would have to be reviewed and approved by the locally responsible ethics committees of each study site. Their reports as well as a signed and dated approval by the BfArM must be obtained and assessed by the leading ethics committee of the TUM before study initiation. Any amendments to the protocol, other than administrative ones (of which the leading ethics committee and BfArM will merely be informed), must be reviewed and approved by both authorities.

Before inclusion of the first patient the federal state authorities (*zuständige Regierungsbehörden der Länder*) will be informed about the study. A copy of this report needs to be filed in the ISF and TMF.

44.3 Ethical performance of the study

The study is conducted according to the ethical principles as defined in the Declaration of Helsinki, version of 2008 (see 28.). The present clinical study is conducted in accordance with principles published in the ICH-GCP Guideline and the applicable legal regulations (AMG, GCP-

V, see 19.1) These principles concern ethics committee procedures, patient information and informed consent procedures, adherence to the protocol, administrative documents, documentation of the study medication, data collection, patient records (source documents), recording and reporting of AEs/SAEs, preparation of inspections and audits as well as storage and safekeeping of the documents. All the investigators and personnel involved in the study have been informed that international monitoring authorities, the competent federal authorities and the sponsor are authorised to review the study documents and patient files.

44.4 Public register

Before the clinical study will be initiated, it will be filed at the German Clinical Trials Register (DRKS), which is part of the International Clinical Trials Registry Platform (ICTRP) of the WHO. After ethical approval the trial will be registered under the ID-number: DRKS00012251.

44.5 Informed consent of the study participants

A patient can only be included in the study, if he provides written consent after being informed by a GCP-trained investigator (orally and in writing) about the nature, significance and scope of the clinical study in an appropriate and understandable way. The investigator must fully explain the purpose of the study to the patient or his/her guardian prior to entering the patient into the study. The investigator is responsible for obtaining written informed consent from each patient The person signing the consent form will receive a copy of the signed form. By providing such consent the patient is declaring that he understands and accepts the recording of data that is part of the study and its verification by authorised monitors or federal authorities. The patient will be educated about the potential benefits and complications of the IMP used in the study. It must be clear for him that he can withdraw his consent at any point of time without any disadvantages to his further treatment. The original copy of the written ICF will be kept in the study folder of the study site. The patient will be given the copies of the written patient information and ICF. Additionally, copies of both documents will be filed in the patient’s medical file. Patient information and ICF are attached at the end of this protocol. The patient information and ICF will be submitted to the responsible ethics committee for assessment before the study will be initiated.

45. INSURANCE FOR TRIAL PARTICIPANTS

In the clinical trial of an IMP, all the participants are insured in accordance with the AMG. The scope of the insurance coverage is derived from the insurance documents that are included in the ISF. Before inclusion the insurance conditions shall be submitted to the patient for review without request to do so. The insurance conditions should be furnished to the patient to take

with him before being included in the study on request and after inclusion in any case. Insurance coverage is being provided by:

HDI-Gerling Industrie Versicherung AG
Niederlassung München
Vertragsservice/M-B
Ganghofer Strasse 37-39
80339 Munich
Tel.: +49 (89) 9243-420
Fax.: +49 (89) 9243-356
Insurance number: 65-963496-03037/390 (Studie: 2/17)

If an insured event is suspected to have occurred, the sponsor is to be notified immediately. He then has to notify the insurance provider about the damages immediately. The patient will receive a copy of the notification to the insurance provider. The patient may also inform the insurance provider by bypassing the study personnel, reporting any claims. In this case, he should be notified that the sponsor of the clinical trial should still be informed about the event. Patients have to be informed about both options.

46. DATA PRIVACY PROTECTION / CONFIDENTIALITY PROTECTION

The applicable local regulations of data privacy protection will be followed. The patients will be informed that any patient-related data and materials will be appropriately made pseudonymous (pursuant to § 12 and § 13 of the GCP Regulations) and that these data may be used for analysis and publication purposes. Furthermore, the patients will be informed that their data may be inspected by representatives of BfArM or of the sponsor for the purpose of validation of a proper study conduct. Patients who do not provide consent for transmission of their data, according to the data protection agreement included in the ICF, will not be included in the clinical study.

47. PROTOCOL AMENDMENTS OR CHANGES IN TRIAL CONDUCT

In order to insure comparable conditions in all study sites and in the interest of standardized evaluations of the trial, changes in this protocol are not foreseen. However, changes in trial conduct are possible. Any change (besides administrative changes) of this protocol requires a written protocol amendment that must be reviewed by the sponsor before implementation. Furthermore, consent needs to be obtained by the investigator of each participating center. Amendments that significantly affect the safety of subjects, the scope of the investigation or the scientific quality of the study, additionally require approval of the leading ethics committee and

BfArM. A copy of the written approval of these amendments must be provided to the sponsor and the investigator at each study site. Examples of amendments requiring such approval are:

- Significant changes in the study design;
- Increases in the number of invasive procedures.

However, these requirements for approval should in no way prevent the investigator or sponsor to take any immediate action in the interests of preserving patient safety. If the investigator feels an immediate change to the protocol is necessary and is implemented for safety reasons, the sponsor, ethics committee and BfArM must be informed immediately. Amendments affecting only administrative aspects of the study do not require formal protocol amendments or ethics committee and BfArM approval. However, the ethics committee and BfArM must still be notified about the changes.

48. STATISTICAL CONSIDERATIONS

48.1 Proposed sample size / Power calculations

Due to the unexpected high number of dropouts, the sample size was adjusted based on the changed analysis of SSI (see section 23.3). The sample size was calculated (Sample Size Software, Sample Size Tables, D. Machin et al., 2009) based on the primary endpoints of the study, assuming SSI rates (event of interest) of 2.2% in the PHX group (assuming a 75% risk reduction according to the trial by Roth *et al*. [1]), 8.7% in the saline group (according to the results of the trial by Cervantes-Sanchez *et al.[*2]), and 16.2% in the control group (according to the meta-analysis by Mueller *et al*. [3]). The incidence rate of SSI over all study arms is then expected to be 7%, given the approximate 3:3:1 group assignment. The actual SSI rate up to now is 7.2%, which is very close to our assumption and we consider it valid. The incidence rate for the competing risks of death or re-laparotomy is estimated to be a total of 13.4% in all arms. This estimation is done based on the data collected up to now.

The global significance level was set to 5% (two-sided tests). Since the PHX arm will be used twice for a comparison, the Bonferroni-Holm procedure was used to set the local alpha level for test 1 (PHX *vs.* no intervention) to 2.5% and for test 2 (PHX *vs.* saline irrigation) to 5%. If 290 patients are recruited in the PHX arm, 290 patients in the saline arm and 100 patients in the control arm (a total of 680 patients, an increase of 140 in the sample size), the two Fine and Gray sub-distributional hazard models will have a power of 80% each to detect differences between the treatment groups. The comparison saline irrigation *vs.* control is not included in the sample size calculation, as it will not be analyzed in a confirmatory manner. The low medical interest cannot justify the large increase in patient numbers.

48.2 Statistical analysis

The primary and secondary endpoints will be analyzed on the Intention-To-Treat (ITT) set, consisting of all patients included in the study in the treatment arm they were randomized to. The safety analysis will be performed on the safety set, consisting of all patients randomized into the study and assigned to the treatment group of their actual treatment.

48.3 Primary endpoint

Wound irrigation with PHX solution will be tested for superiority over no irrigation (Test 1) and irrigation with saline (Test 2) with respect to the incidence of SSI within 30 days of surgery using two Fine and Gray sub-distributional hazard models with SSI as main event and relaparotomy and death as competing risks. Since randomization is stratified by study centre and level of contamination, the models will include covariates treatment group, study centre, and level of contamination. The global significance level is set to 5%. Using the Bonferroni-Holm adjustment, the local significance level will be 2.5% and 5% in the order of increasing p-value.

48.4 Supportive analysis of the primary endpoint

Since randomization will be stratified by study center and level of contamination, supportive analysis of the primary endpoint will also be performed using a binary logistic regression model with dependent variable SSI and covariates treatment group, study center, and level of contamination. In case there are differences between the treatment groups in terms of baseline characteristics, those will also be included as covariates in the model. Operation related risk factors (*e.g.* type and duration of surgery, administration and timing of antibiotic prophylaxis, use of wound-edge protectors, intraoperative changing of gloves, presence of an ostomy) and patient related risk factors (*e.g.* NNIS risk score, ASA, BMI, age, diabetes, smoking, alcohol consumption, duration of preoperative hospital stay, history of SSI, history of radio/chemotherapy) might influence the outcome, which is why they will also be included as model covariates.

Additionally, the incidence rates within 30 days for SSI, re-laparotomy, death, and lost to follow-up for other reasons will be displayed per treatment group and compared using Fisher's exact test in order to better understand the distribution of missing values.

48.5 Secondary endpoints

Secondary endpoints will be analyzed by treatment group on the ITT set, using appropriate descriptive statistics. Any explorative statistical testing will be performed using a significance level of 5%. Subgroup analyses or treatment group comparisons will be performed for rate of superficial/deep/organ space SSI (according to CDC [1], attachment 1) stratified by the NNIS

risk score, level of contamination (class II,III or IV) during surgery (according to CDC [1] attachment 3) ASA score, BMI, age, diabetes, smoking, alcohol consumption, history of SSI, history of radio-/chemotherapy, preoperative hospital stay >2d, administration and timing of antibiotic prophylaxis, type and duration of surgery, intraoperative use of wound-edge protectors and changing of gloves, presence of an enterostomy by use of a binary logistic regression model with a main effect for treatment, the subgroup defining variable and a respective interaction effect. Description of treatment costs will be summarized as additional costs with respect to the no intervention group. All AEs including SSI and local wound complications will be analyzed with incidence rates by treatment group and according to severity. AEs rated as related to the study treatment will be listed separately. In addition, the duration of hospital stay in days will be compared between the three study groups.

48.6 Missing data

Missing primary endpoint data in the primary analysis will be dealt with using competing risks and censoring. Missing SSI evaluation due to death or relaparotomy will be considered a competing risk. Missing SSI for all other reasons will be censored. Data will not be imputed for other analyses such as secondary or subgroup analyses.

49. RANDOMIZATION AND METHODS AGAINST BIAS

Participating, GCP-certified investigators will perform the screening and recruitment of patients and will obtain the ICF prior to inclusion. Every patient fulfilling inclusion and exclusion criteria will be documented. Reasons for non-inclusion into the study will have to be documented as well in a screening-list. A GCP-trained member of the study group will perform randomization during surgery after closure of the abdominal fascia is completed using RANDOBASE, the online-randomization tool at MSZ. RANDOBASE uses pre-defined randomization lists, which will be created at IMSE and will be stratified by level of contamination of the surgical procedure (clean-contaminated, contaminated or dirty) and by study center. To assure balanced group sizes in the course of the accrual, a block-wise randomization is applied. Basic characteristics of the patient and day of randomization must be documented on the randomization sheets. Subsequently, randomization sheets must be printed out, dated, signed and stored away from the patient records, trial documents and ISF to ensure blinding. Details on the blinding procedure are presented under point 11.4.

50. FINAL REPORTING

After completion of the trial, BfArM and the leading ethics committee (TUM) have to be informed within 90 days by a final study report. Within one year of the completion of the trial, BfArM and the ethics committee will be supplied with a summary of the final report on the clinical trial containing the principle results. The sponsor is responsible for the generation of these final reports.

51. PUBLICATION OF STUDY RESULTS

After completion of the clinical study, a multi-center manuscript of the study results will be prepared for publication in a reputable scientific journal according to the CONSORT statement. For this manuscript, final analyses will be generated from the study database and it will be subject to review by the sponsor. The publication of the principal results from any single center experience within the trial is not allowed until the preparation and publication of the multi-center results. Exceptions to this rule require prior approval of the sponsor. For purposes of abstract presentation and publication, any secondary publications will be delegated to the appropriate principal authors. However, final analyses and manuscript review for all multi-center data will require the approval of the sponsor. The use of professional writers is not intended. Details on publication rules and author order will be provided in the Clinical Trial Agreement.

52. DECLARATION OF HELSINKI

The Declaration of Helsinki, 2008 (Seoul), is attached to the protocol.

53. ATTACHMENTS

Attachment 1: Definition and classification of SSI according to CDC

**Superficial Incisional SSI**	Infection occurs within 30 days after the operation **and** infection involves only skin or subcutaneous tissue of the incision **and** at least ***one*** of the following:<br><br>5 Purulent drainage, with or without laboratory confirmation, from the superficial incision.<br>6 Organisms isolated from an aseptically obtained culture of fluid or tissue from the superficial incision.<br>7 At least one of the following signs or symptoms of infection: pain or tenderness, localized swelling, redness, or heat *and* superficial incision is deliberately opened by surgeon, *unless* incision is culture-negative.<br>8 Diagnosis of superficial incisional SSI by the surgeon or attending physician.<br><br>*Notes:*<br>Do *not* report the following conditions as SSI:<br>2 Stitch abscess (minimal inflammation and discharge confined to the points of suture penetration).
**Deep Incisional SSI**	Infection occurs within 30 days after the operation **and** the infection appears to be related to the operation **and** infection involves deep soft tissues (*e.g*. fascial and muscle layers) of the incision **and** at least *one* of the following:<br><br>6 Purulent drainage from the deep incision but not from the organ/space component of the surgical site.<br>7 A deep incision spontaneously dehisces or is deliberately opened by a surgeon when the patient has at least one of the following signs or<br>8 Symptoms: fever (>38°C), localized pain, or tenderness, unless site is culture-negative.<br>9 An abscess or other evidence of infection involving the deep incision is found on direct examination, during reoperation, or by histopathologic or radiologic examination.<br>10 Diagnosis of a deep incisional SSI by a surgeon or attending physician.<br><br>*Notes:*<br>3 Report infection that involves both superficial and deep incision sites as deep incisional SSI.<br>4 Report an organ/space SSI that drains through the incision as a deep incisional SSI.
**Organ/Space SSI**	Infection occurs within 30 days after the operation and the infection appears to be related to the operation **and** infection involves any part of the anatomy (*e.g.,* organs or spaces), other than the incision, which was opened or manipulated during an operation **and** at least *one* of the following:<br><br>5 Purulent drainage from a drain that is placed through a stab wound into the organ/space.<br>6 Organisms isolated from an aseptically obtained culture of fluid or tissue in the organ/space.<br>7 An abscess or other evidence of infection involving the organ/space that is found on direct examination, during reoperation, or by histopathologic or radiologic examination.<br>8 Diagnosis of an organ/space SSI by a surgeon or attending physician.

Attachment 2: Clavien Dindo classification of surgical complications

Grade	Definition
Grade I	Any deviation from the normal postoperative course without the need for pharmacological treatment or surgical, endoscopic, and radiological interventions
Grade II	Allowed therapeutic regimens are: drugs as antiemetics, antipyretics, analgetics, diuretics, electrolytes, and physiotherapy. This grade also includes wound infections opened at the bedside
Grade III	Requiring pharmacological treatment with drugs other than such allowed for grade I complications Blood transfusions and total parenteral nutrition are also included Requiring surgical, endoscopic or radiological intervention
Grade IIIa	Intervention not under general anesthesia
Grade IIIb	Intervention under general anesthesia
Grade IV	Life-threatening complication (including CNS complications)* requiring IC/ICU management
Grade IVa	Single organ dysfunction (including dialysis)
Grade IVb	Multiorgan dysfunction
Grade V	Death of a patient
Suffix “d”	If the patient suffers from a complication at the time of discharge, the suffix “d” (for “disability”) is added to the respective grade of complication. This label indicates the need for a follow-up to fully evaluate the complication.
	*Brain hemorrhage, ischemic stroke, subarrachnoidal bleeding, but excluding transient ischemic attacks. CNS, central nervous system; IC, intermediate care; ICU, intensive care unit

Attachment 3. Classification of wound contamination levels according to CDC

Class I/ Clean	These are uninfected operative wounds in which no inflammation is encountered and the respiratory, alimentary, genital, or uninfected urinary tracts are not entered. In addition, clean wounds are primarily closed, and if necessary, drained with closed drainage. Operative incisional wounds that follow non-penetrating (blunt) trauma should be included in this category if they meet the criteria. Laparoscopic surgeries, surgeries involving the skin (such as biopsies), eye or vascular surgeries are good examples.
Class II/ Clean-Contaminated	**An operative wound in which the respiratory, alimentary, genital, or urinary tracts are entered under controlled conditions and without unusual contamination.** Specifically, operations involving the biliary tract, appendix, vagina, and oropharynx are included in this category, provided no evidence of infection or major break in technique is encountered.
Class III/ Contaminated	Open, fresh, accidental wounds. In addition, **operations with major breaks in sterile technique (e.g., open cardiac massage) or gross spillage from the gastrointestinal tract, and incisions in which acute, non-purulent inflammation is encountered are included in this category**. Contaminated wounds are also created when an outside object comes in contact with the wound (e.g. a bullet, knife blade or other pointy object).
Class IV/ Dirty-Infected	Old traumatic wounds with retained devitalized tissue and those that involve existing clinical infection or perforated viscera or a foreign object lodged in the wound **or any wound that has been exposed to pus or fecal matter.** This definition suggests that the organisms causing postoperative infection were present in the operative field before the operation.

This classification scheme has been shown in numerous studies to predict the relative probability that a wound will become infected. Clean wounds have a 1-5% risk of infection; clean-contaminated 3-11%; contaminated, 10-17%; and dirty over 27% (CDC).

Attachment 4: ASA classification

ASA Score	Patient's Preoperative Physical Status
1	Normally healthy patient
2	Patient with mild systemic disease
3	Patient with severe systemic disease that is not incapacitating
4	Patient with an incapacitating systemic disease that is a constant threat to life
5	Moribund patient who is not expected to survive for 24 hours with or without operation

Attachment 5: NNIS risk index

The NNIS risk index is operation-specific and applied to prospectively collected surveillance data. The index values range from 0 to 3 points and are defined by three independent and equally weighted variables. 0 indicating the lowest and 3 the highest risk of SSI.

One point is scored for each of the following when present:

(1) American Society of Anesthesiologists (ASA) Physical Status Classification of >2
(2) Either contaminated or dirty/infected wound classification (class III and IV)
(3) Length of operation >T hours, where T is the approximate 75th percentile of the duration of the specific operation being performed.

The T Point for Common Surgical Procedures (NNIS report 2004)

Operation	T Point (hrs)
Bile duct, liver, or pancreatic surgery	5
Colonic surgery	3
Herniorrhaphy	2
Appendectomy	1
Other digestive	2
Laparotomy	2
Small bowel	3
Splenectomy	3
Cholecystectomy	2
Gastric	3
Nephrectomy	4
Organ transplant	6

54. REFERENCES

1. Mangram, A.J., et al., *Guideline for Prevention of Surgical Site Infection, 1999. Centers for Disease Control and Prevention (CDC) Hospital Infection Control Practices Advisory Committee.* Am J Infect Control, 1999. **27**(2): p. 97-132; quiz 133-4; discussion 96.
2. Mihaljevic, A.L., et al., *Multicenter Double-Blinded Randomized Controlled Trial of Standard Abdominal Wound Edge Protection With Surgical Dressings Versus Coverage With a Sterile Circular Polyethylene Drape for Prevention of Surgical Site Infections: A CHIR-Net Trial (BaFO; NCT01181206).* Ann Surg, 2014. **260**(5): p. 730-9.
3. Diener, M.K., et al., *Effectiveness of triclosan-coated PDS Plus versus uncoated PDS II sutures for prevention of surgical site infection after abdominal wall closure: the randomised controlled PROUD trial.* Lancet, 2014. **384**(9938): p. 142-52.
4. Pinkney, T.D., et al., *Impact of wound edge protection devices on surgical site infection after laparotomy: multicentre randomised controlled trial (ROSSINI Trial).* Bmj, 2013. **347**: p. f4305.
5. Barnes, S., et al., *Surgical wound irrigation: A call for evidence-based standardization of practice.* American Journal of Infection Control, 2014. **42**(5): p. 525-529.
6. *Global Guidelines for the Prevention of Surgical Site Infection*. 2016, World Heath Orgnaization: Geneva.
7. *National Institute for Health and Clinical Excellence (NICE). Surgical Site Infection - Prevention and Treatment of Surgical Site Infection. NICE Clinical Guideline 74.* 2008, RCGO Press: London, England.
8. Rohner, E., et al., *Preferred use of polyhexanide in orthopedic surgery.* Orthopedics, 2011. **34**(10): p. e664-8.
9. Roth, B., et al., *[Recommendations for the use of polyhexanide-containing products for the treatment of wounds].* Praxis (Bern 1994), 2011. **100**(9): p. 531-7.
10. Fournel, I., et al., *Meta-analysis of intraoperative povidone-iodine application to prevent surgical-site infection.* Br J Surg, 2010. **97**(11): p. 1603-13.
11. Chundamala, J. and J.G. Wright, *The efficacy and risks of using povidone-iodine irrigation to prevent surgical site infection: an evidence-based review.* Can J Surg, 2007. **50**(6): p. 473-81.
12. Charalambous, C.P., et al., *When should old therapies be abandoned? A modern look at old studies on topical ampicillin.* J Infect, 2003. **47**(3): p. 203-9.
13. McHugh, S.M., et al., *The role of topical antibiotics used as prophylaxis in surgical site infection prevention.* J Antimicrob Chemother, 2011. **66**(4): p. 693-701.
14. Mueller, T.C., et al., *Intra-operative wound irrigation to reduce surgical site infections after abdominal surgery: a systematic review and meta-analysis.* Langenbecks Arch Surg, 2015.
15. Cervantes-Sanchez, C.R., et al., *Syringe pressure irrigation of subdermic tissue after appendectomy to decrease the incidence of postoperative wound infection.* World J Surg, 2000. **24**(1): p. 38-41; discussion 41-2.
16. Roth B, A.O., Wurmitzer F, Kramer A, *[Surgical site infections after primary antiseptic cleansing of dirty-contaminated wounds by polihexanide, PVP iodine resp. hydrogen peroxide].* GMS Krankenhaushyg Interdiszip 2007. **2**(2): p. Doc58.
17. Wundinfektionen, K.S.S.p. 2013 [cited 2014; Available from: http://www.nrz-hygiene.de/surveillance/kiss/.
18. de Lissovoy, G., et al., *Surgical site infection: incidence and impact on hospital utilization and treatment costs.* Am J Infect Control, 2009. **37**(5): p. 387-97.
19. Gastmeier, P., et al., *Postoperative Wundinfektionen nach stationären und ambulanten Operationen.* Bundesgesundheitsblatt - Gesundheitsforschung - Gesundheitsschutz, 2004. **47**(4): p. 339-344.
20. Geffers, C., M.D., *Postoperative Wundinfektionen*, B.M. e.V., Editor. 2011, Institut für Hygiene und Umweltmedizin der Charité, Berlin.
21. Mihaljevic, A.L., et al., *Wound edge protectors in open abdominal surgery to reduce surgical site infections: a systematic review and meta-analysis.* PLoS One, 2015. **10**(3): p. e0121187.
22. Clavien, P.A., et al., *The Clavien-Dindo classification of surgical complications: five-year experience.* Ann Surg, 2009. **250**(2): p. 187-96.
23. *Gesetz über den Verkehr mit Arzneimitteln (Arzneimittelgesetz - AMG)*. 2005, Bundesministerium für Justiz und Verbraucherschutz.
24. *International Conference on Harmonisation of Technical Requirements for Registration of Pharmaceuticals for Human Use, ICH E6*. 1996, International Conference on Harmonisation; http://www.ich.org: Geneva.
25. Chan, A.W., et al., *SPIRIT 2013 statement: defining standard protocol items for clinical trials.* Ann Intern Med, 2013. **158**(3): p. 200-7.

1.3 CSP summary of changes

Approvals and Amendments

Initial Approval: Bundesinstitut für Arzneimittel und Medizinprodukte (BfArM): 27.06.2017 (Nr. 4042099); Ethics Committee (EC): 27.06.2017 (Nr. 173/17-Af) Clinical Study Protocol (CSP) Version (V) 2.0 06.06.2017

Amendment 1: Addition of Study sites #11 Würzburg and #12 Mannheim
Approval of AM1: EC: 14.05.2019

Amendment 2: Major changes: Extension of study duration, Changes in statistical analysis plan because of an unexpected high number of drop-outs due to relaparotomy. To maintain statistical power, the sample size had to be increased to 680.
Approval AM2: BfArM: 08.03.2021 EC: 24.03.2021, CSP V 3.0 02.03.2021
During the course of the clinical trial BfArM/EC were informed of/approved further changes (e.g. due to addition of study sites and changes of PIs).

2. Statistical Analysis Plan (SAP)

2.1. SAP Original Version 1.0 (28.04.2021)

Table of Contents

1 **INTRODUCTION** 86
1.1 Background and rationale 86
1.2 Study objectives 86
1.3 Study endpoints 86
2 **STUDY METHODS** 87
2.1 Trial design 87
2.2 Randomization 87
2.3 Sample size 87
2.4 Framework 88
2.5 Statistical interim analyses and stopping guidance 88
2.6 Timing of final analysis 88
2.7 Timing of outcome assessments 88
3 **STATISTICAL PRINCIPLES** 89
3.1 Confidence intervals and P values 89
3.2 General calculation rules 89
3.3 Adherence and protocol deviations 89
3.4 Analysis populations 89
3.5 Event and censor times for the Kaplan-Meier analyses 89
4 **ANALYSIS** 90
4.1 Trial population 90
4.1.1 Screening data 90
4.1.2 Eligibility 90
4.1.3 Recruitment 90
4.1.4 Withdrawal/follow-up 90
4.1.5 Baseline patient characteristics 91
4.2 Outcome definitions 91
4.3 Analysis methods for the primary and secondary endpoints 92
4.3.1 Primary endpoint 92
4.3.2 Supportive analysis of the primary endpoint 92
4.3.3 Secondary endpoint analysis 92
4.3.3.1 Non-infectious wound complications 93
4.3.3.2 Hospital stay 93
4.3.3.3 Thirty-day mortality 93
4.3.3.4 Re-operation rate 93

4.3.3.5 Subgroup analysis ... 93
4.3.3.6 Treatment costs ... 94
4.3.3.7 Adverse events ... 94
4.4 Missing data ... 94
4.5 Additional analyses ... 94
4.6 Statistical software ... 94
5 TABLE SHELLS ... 95

2 INTRODUCTION

2.1 Background and rationale

Surgical site infection (SSI) is one of the most common complications following abdominal visceral surgery and dramatically increases length of hospital stay and costs. Hypothetically, intraoperative wound irrigation (IOWI) before skin closure with saline or antiseptics might be a potential pragmatic option to reduce SSI rates. Currently, there are no official recommendations on its use and clinical practice varies largely. Solutions containing the antiseptic agent polyhexanide (PHX) are approved for IOWI, and were shown to promote wound healing, but have not been evaluated in RCTs in abdominal visceral surgery. Therefore, we designed a multicenter, randomized, observer-blinded clinical trial evaluating the efficacy of IOWI with PHX solution or saline before skin closure after laparotomy. The primary endpoint is the incidence of SSI 30 days postoperatively, according to the CDC definition. The results of the trial will provide evidence for definite clinical recommendations regarding the use of IOWI and influence current guidelines and provide all participating patients the opportunity of an improved treatment.

2.2 Study objectives

To investigate whether the use of intraoperative, epifascial wound irrigation with PHX solution can reduce surgical site infections after laparotomy for visceral surgery compared to saline irrigation or no irrigation.

2.3 Study endpoints

<u>Primary efficacy endpoint</u>:

SSI according to CDC criteria within 30 days postoperatively

<u>Secondary endpoints</u>:

- Non-infectious wound complications (e.g. seroma, hematoma, delayed healing) within 30 days postoperatively
- Duration of hospital stay
- Mortality and morbidity within 30 days postoperatively
- Incidence of reoperation within 30 days postoperatively
- Incidence of AE/SAE within 30 days postoperatively
    - Surgical complications will be additionally evaluated according to the Clavien-Dindo classification.

Pre-specified subgroup analysis by category of SSI (superficial, deep, organ space), NNSI risk score, ASA score, BMI, age, diabetes, smoking, alcohol consumption, history of SSI, history of radio-/chemotherapy, pre-operative hospital stay >2d, administration and timing of antibiotic prophylaxis, type and duration of surgery, intraoperative use of wound-edge protectors and changing of gloves, presence of an enterostomy.

3 STUDY METHODS

3.1 Trial design

This study is prospective, randomized, controlled, observer and patient-blinded, multicenter, surgical trial according to German drug law (AMG) phase III-b, with three parallel comparison groups.

Patients are randomised to one of the following treatment arms:

Arm 1 (Intervention 1):

Irrigation of the subcutaneous tissue after closure of the abdominal fascia with 1000ml PHX solution (0.04%)

Arm 2 (Intervention 2):

Irrigation of the subcutaneous tissue after closure of the abdominal fascia with 1000ml saline solution (NaCl 0.9%)

Arm 3 (Control):

No epifascial wound irrigation

A total of 680 patients (290 patients in arm 1, 290 patients in arm 2, and 100 patients in arm 3) in up to 15 centres will be enrolled in the study with duration of 34 days per patient (up to three days prior to surgery, day of surgery, and 30 days post-surgery).

A total of 8 visits are scheduled during the study period.

3.2 Randomization

Patients are randomised blockwise ca. 3:3:1 to the treatment arms with stratification by centre and level of contamination of the surgical procedure (clean-contaminated, contaminated, or dirty) during surgery after closure of the abdominal fascia using RANDOBASE, the online-randomization tool at MSZ. RANDOBASE uses pre-defined randomization lists, which are created at IMedIS using Rancode Professional 2015. Two sets of sealed envelopes were produced: one for emergency unblinding at site, one for the same purpose at the MSZ-safety management department.

3.3 Sample size

Justification and calculation of the sample size can be found in the study protocol section 23.1.

Sample size adjustment (CSP approved Version 3 Amendment 2 02.03.2021):

Due to the unexpected high number of dropouts, the sample size was adjusted based on the changed analysis of SSI (see section 5.2.1). The sample size was calculated (Sample Size Software, Sample Size Tables, D. Machin et al., 2009) based on the primary endpoints of the study, assuming SSI rates (event of interest) of 2.2% in the PHX group (assuming a 75% risk reduction according to the trial by Roth et al. (14)), 8.7% in the saline group (according to the results of the trial by Cervantes-Sanchez et al. (13)), and 16.2% in the control group according to results of the previously conducted meta-analysis (12). The incidence rate of SSI over all study arms is then expected to be 7%, given the approximate 3:3:1 group assignment. The actual SSI rate up to now is 7.2%, which is very close to our assumption and we consider it valid. The

incidence rate for the competing risks of death or re-laparotomy is estimated to be a total of 13.4% in all arms. This estimation is done based on the data collected up to now.

The global significance level was set to 5% (two-sided tests). Since the PHX arm will be used twice for a comparison, the Bonferroni-Holm procedure was used to set the local alpha level for test 1 (PHX vs. no intervention) to 2.5% and for test 2 (PHX vs. saline irrigation) to 5%. If 290 patients are recruited in the PHX arm, 290 patients in the saline arm and 100 patients in the control arm (a total of 680 patients), the two Fine and Gray sub-distributional hazard models will have a power of 80% each to detect differences between the treatments.

3.4 Framework

This study tests for superiority of (1) PHX over no intervention and (2) PHX over saline irrigation with respect to SSI rate within 30 days postoperatively.

3.5 Statistical interim analyses and stopping guidance

No interim analyses are planned for this study.

3.6 Timing of final analysis

The final analysis will be performed collectively at the end of the study.

3.7 Timing of outcome assessments

Baseline data will be collected at visit 1 and 2, which means up to day 0**.**

Day 0 is defined as the time of closure of the abdominal fascia.

All other day definitions are relative to day 0.

The primary outcome will be accessed up to visit 8, which means on day 30 and up to day 36 when the time window is considered.

Visit	Day	Time window
1	-3 to -1	Up to day 0
2	0 (surgery)	
3	2	
4	4	
5	6	
6	8	
7	10	Up to day 14
8	30	Up to day 36

4 STATISTICAL PRINCIPLES

4.1 Confidence intervals and P values

All statistical tests will be performed two-sided at the global significance level of 5%. Adjustment for multiplicity will be done for the primary endpoint only, where the Bonferroni-Holm adjustment method will be used (see also section 5.2.1).

Confidence intervals will be two-sided and 95%.

4.2 General calculation rules

1. Percentages will always be quoted using number of 'known' values in the denominator unless otherwise stated.
2. P-values will be quoted to three decimal places only. Confidence intervals will also be quoted to three decimal places. However, if the statistical software SAS, which is used for analysis, prints four decimal places, values will not be rounded again, but printed to four decimal places.
3. Chi-square tests in contingency tables will be replaced by Fisher’s exact tests if any expected cell frequency is less than five.

4.3 Adherence and protocol deviations

The number and percent of patients per treatment group who received IOWI will be reported.

Major protocol deviations will be listed per patient.

4.4 Analysis populations

The **Intention-to-Treat (ITT) population** will contain all randomised patients with results attributed to the treatment group they were randomised to.

The **safety analysis (SA) population** will consist of all randomised subjects with results attributed to the treatment group of their actual treatment.

Safety analysis will be performed on the SA population. All other analyses will be performed in the ITT population.

4.5 Event and censor times for the Kaplan-Meier analyses

The following definitions will apply to the time-to-event analysis of SSI:

**Parameter:**	SSI
**Main Event Time**	time of SSI
**Competing Event Times**	time of death<br>time of re-laparotomy
**Censor Time**	time of last follow-up[*]/ time of competing event[#]

* for patients without SSI

\# for patients with re-laparotomy and for patients who died

5 ANALYSIS

5.1 Trial population

5.1.1 Screening data

The overall number of screened patients will be presented in the report.

The reasons for non-eligibility will also be summarized.

5.1.2 Eligibility

Key inclusion criteria:

- Clean-contaminated, contaminated or dirty (according to CDC classification)
- Abdominal surgery by midline or transverse laparotomy (elective or emergency)
- Age ≥18 years
- American Society of Anesthesiologists (ASA) score ≤ 3

Key exclusion criteria:

- Pregnancy or breast feeding
- Known hypersensitivity/allergy to PHX
- Inability to understand/give informed consent
- Inability to attend follow-up visits
- Revision-surgery (previous abdominal surgery within the last 30 days)
- Planned re-laparotomy within 30 days
- Severe immunosuppression
- Concurrent abdominal wall infections
- Pre-operative antibiotic therapy (within 5 days prior to surgery)

The full set of inclusion and exclusion criteria can be found in the study protocol sections 12.1 and 12.2.

5.1.3 Recruitment

Tables will contain the following absolute and relative frequencies per treatment group and overall:

- patients who entered the study.
- patients within each analysis set including reasons for exclusion. Patients in the SA will be considered in the group of their actual treatment.
- patients per centre on the ITT set.

5.1.4 Withdrawal/follow-up

Since study treatment is given only once at the beginning of the study, withdrawal from study treatment is not of interest.

The number of patients per treatment group who did not complete the study will be given in a summary table by reason (multiple reasons possible) including absolute and relative frequencies.

5.1.5 Baseline patient characteristics

The following characteristics will be summarized per treatment group on the ITT set:

Demographics: sex, age, BMI.

Medical history: main diagnosis leading to operation (malign/benign), ASA classification, diabetes (including type and treatment), allergies, comorbidities (11 pre-defined and other; multiple comorbidities are possible), previous abdominal surgery (no, single, multiple), time since last abdominal surgery, history of SSI (no, single, multiple), time since last SSI, location of last SSI, history of radiotherapy (no, single, multiple), time since end of last radiotherapy, dose (Gy), history of chemotherapy (no, single, multiple), smoking (no, previously, currently), packyears, regular alcohol consumption (no, previously, currently), glasses/week.

Surgery: duration of preoperative hospital stay (days), urgency and type of procedure, duration of surgery (min), antibiotic prophylaxis (no, yes >1h prior OP, yes ≤1h prior OP), type of skin disinfectant, type of incision, length of incision, intra-OP change of gloves (y/n), intra-OP use of wound edge protectors (y/n), enterostomy created (y/n), type of abdominal fascia closure, use of mesh (no, yes-sublay, yes-onlay), level of contamination (class I to IV), NNIS risk score, wound closure (complete/incomplete), subcutaneous sutures used (y/n), skin closure type (stapler, continuous suture, single suture).

Absolute and relative frequencies will be presented for categorical variables. Number of valid cases, mean, standard deviation, median, minimum, and maximum will be displayed for continuous variables.

5.2 Outcome definitions

The primary endpoint is the frequency of physician-assessed SSI up to 30 days post-surgery. According to the time-definitions, the SSI assessment may take place up to day 36. Surgical site infections will additionally be classified in three groups: superficial incisional, deep incisional, and organ/space.

The secondary endpoints of this study are:

Frequency of non-infectious wound complications (e.g. seroma, hematoma, delayed healing) within 30 days postoperatively;

Duration of hospital stay;

Mortality within 30 days postoperatively;

Rate of reoperation within 30 days post-surgery, which will be identified during medical review based on the AE records.

Pre-specified subgroup analysis by category of SSI (superficial, deep, organ space), NNSI risk score, ASA score, BMI, age, diabetes, smoking, alcohol consumption, history of SSI, history of radio-/chemotherapy, pre-operative hospital stay >2d, administration and timing of antibiotic prophylaxis, type and duration of surgery, intraoperative use of wound-edge protectors and changing of gloves, presence of an enterostomy.

Assessment of safety:

The assessment of safety will be based on the frequency of AE/SAE other than SSI within the safety population (according to CTCAE V. 4.3). Surgical complications will be additionally evaluated according to the Clavien-Dindo classification.

5.3 Analysis methods for the primary and secondary endpoints

5.3.1 Primary endpoint

Wound irrigation with PHX solution will be tested for superiority over no irrigation (Test 1) and irrigation with saline (Test 2) with respect to the incidence of SSI within 30 days of surgery using two Fine and Gray sub-distributional hazard models with SSI as main event and re-laparotomy and death as competing risks. The model actually compares time to event, although the timing of SSI is of less interest than the occurrence of SSI within 30 days of surgery.

Since randomization is stratified by study center and level of contamination, the models will include covariates treatment group, study center, and level of contamination. The global significance level is set to 5%. Using the Bonferroni-Holm adjustment, the local significance level will be 2.5% and 5% in the order of increasing p-value.

In case there are small study centers which would not allow the model to converge, center will not be used as covariate.

5.3.2 Supportive analysis of the primary endpoint

The frequency of SSI will be presented graphically by treatment and study center using a clustered bar chart.

In case there are differences between the treatment groups in terms of baseline characteristics, those will also be included as covariates in the models. Type and duration of operation, use of wound-edge protectors, intraoperative changing of gloves and patient related risk factors (NNSI risk score, BMI, age, diabetes) might influence the outcome, which is why they will also be included as model covariates.

Descriptive statistics for the primary endpoint will be presented per treatment group: absolute and relative frequencies of

- SSI (overall and by class: superficial incisional, deep incisional, and organ/space)
- Re-laparotomy (will be identified during medical review based on the AE records)
- Death
- Lost to follow-up
- Completed study without event

Those incidences will be compared between treatment groups using Fisher's exact test in order to better understand the distribution of missing values.

5.3.3 Secondary endpoint analysis

Secondary endpoints will be analysed by study group on the ITT set using appropriate descriptive statistics. Any explorative statistical testing will be performed two-sided using a significance level of 5%.

5.3.3.1 Non-infectious wound complications

Non-infectious wound complications (seroma, hematoma, delayed healing, necrosis) within 30 days postoperatively will be summarized by treatment group using absolute and relative frequencies. The two types of irrigation will be compared to no irrigation using the χ^2-test or the Fisher exact test, as appropriate.

5.3.3.2 Hospital stay

Duration of hospital stay – overall and post-surgery. The later will be calculated by subtracting the duration of preoperative hospital stay from the (overall) duration of hospital stay, recorded at study end (visit 8). All durations will be measured in days. Number of valid cases, mean, standard deviation, median, minimum, and maximum of post-surgical hospital stay will be displayed per treatment group. The two types of irrigation will be compared to no irrigation using the independent t-test or the Mann-Whitney-U test, as appropriate.

5.3.3.3 Thirty-day mortality

Mortality within 30 days postoperatively will be summarized by treatment group using absolute and relative frequencies. The two types of irrigation will be compared to no irrigation using the χ^2-test or the Fisher exact test, as appropriate.

5.3.3.4 Re-operation rate

Rate of reoperation within 30 days post-surgery will be identified during medical review based on the AE records and will be summarized by treatment group using absolute and relative frequencies. The two types of irrigation will be compared to no irrigation using the χ^2-test or the Fisher exact test, as appropriate.

5.3.3.5 Subgroup analysis

The primary and secondary endpoints will be additionally summarized within the following subgroups:

- Type of SSI (superficial, deep, organ space)
- NNSI - surgical infection risk index (0, 1, 2, 3)
- ASA score (1, 2, 3)
- BMI (<18.5, 18.5≤BMI<25, 25≤BMI<30, ≥30)
- Age (18≤age<40, 40≤age<65, 65≤age<85, ≥85)
- Diabetes (Type I, Type II - insulin, Type II - oral antidiabetics, Type II - dietary)
- Smoker (current, former, no)
- Alcohol consumption (current, former, no)
- History of SSI (multiple, single, no)
- History of radiotherapy (multiple, single, no)
- History of chemotherapy (multiple, single, no)
- Preoperative hospital stay >2d (y/n)
- Antibiotic prophylaxis (yes >1h prior OP, yes ≤1h prior OP, no)

- Type of surgery (intestinal, hepato-biliary, other):

  1 = Colo-rectal= colon, rectum, appendix
  2 = Hepato-bilary= pancreas, bile duct, hepatic
  3 = Oesophago-gastric = esophageal and gastric
  4 = Other

- Duration of surgery taken from NNIS: Length of OP >T hours (y/n)
- Intraoperative use of wound-edge protectors (y/n)
- Intraoperative changing of gloves (y/n)
- Presence of an enterostomy (y/n)
- Level of contamination (Class II, III, IV)

Absolute and relative frequencies will be presented for categorical variables. Number of valid cases, mean, standard deviation, median, minimum, and maximum will be displayed for continuous variables. Tests will only be performed in case clinically meaningful differences are observed between treatment groups.

5.3.3.6 Treatment costs

Analysis of treatment costs will be done indirectly through the between-group comparisons of hospital stay and surgical complications. This analysis is already described in sections 4.3.3.1, 4.3.3.2, 4.3.3.4, 4.3.1 and 4.3.2.

5.3.3.7 Adverse events

All AEs other than SSI will be analysed on the safety set. The overall incidence of non-SAE-adverse events, SAEs, and related SAEs will be tabulated using MedDRA System Organ Class and Preferred Term by treatment group (see table shells). The overall occurrences as well as the number of affected patients are of interest. SAEs and related SAEs which resulted in death will also be tabulated.

5.4 Missing data

Missing primary endpoint data in the primary analysis will be dealt with using competing risks and censoring. Missing SSI evaluation due to death or re-laparotomy will be considered a competing risk. Missing SSI for all other reasons will be censored. Data will not be imputed for other analyses such as secondary or subgroup analyses.

5.5 Additional analyses

The following parameters will be summarized by treatment group using absolute and relative frequencies: use of concomitant medications of special interest (antibiotics, immunosuppression, anticoagulants), SSI (no/yes-new/yes-ongoing), Type of SSI (including their Clavien-Dindo classification), non-SSI wound complications (seroma, hematoma, delayed healing, necrosis, other wound intervention (none, VAC, bedside wound revision, re-operation, other), abnormal lab values.

The vital parameters body temperature, systolic blood pressure, diastolic blood pressure, and pulse will be summarized by treatment group and visit using mean and SD.

5.6 Statistical software

Analysis will be performed with SAS version 9.4.

6 TABLE SHELLS

SAE, related-SAE, SAE resulting in death, related-SAE resulting in death, and non-SAE-AEs (5 tables)

System Organ Class<br>Preferred Term	Exposed to Treatment 1 N=			Exposed to Treatment 2 N=			Exposed to Treatment 3 N=		
	Events	Subjects affected		Events	Subjects affected		Events	Subjects affected	
		n	%*		n	%*		n	%*
OVERALL	x	x	(x)	x	x	(x)	x	x	(x)
SOC1	x	x	(x)	x	x	(x)	x	x	(x)
PT1	x	x	(x)	x	x	(x)	x	x	(x)
PT2	x	x	(x)	x	x	(x)	x	x	(x)
PT3	x	x	(x)	x	x	(x)	x	x	(x)
SOC2	x	x	(x)	x	x	(x)	x	x	(x)
PT4	x	x	(x)	x	x	(x)	x	x	(x)
PT5	x	x	(x)	x	x	(x)	x	x	(x)
…									

** with respect to the number of exposed subjects*

Subjects enrolled per age group

Age group	Treatment 1		Treatment 2		Treatment 3		Total
	n	%	n	%	n	%	n
Total	x	(x)	x	(x)	x	(x)	x
In utero	x	(x)	x	(x)	x	(x)	x
Preterm newborn - gestational age < 37 wk	x	(x)	x	(x)	x	(x)	x
Newborns (0-27 days)	x	(x)	x	(x)	x	(x)	x
Infants and toddlers (28 days-23 months)	x	(x)	x	(x)	x	(x)	x
Children (2-11 years)	x	(x)	x	(x)	x	(x)	x
Adolescents (12-17 years)	x	(x)	x	(x)	x	(x)	x
Adults (18-64 years)	x	(x)	x	(x)	x	(x)	x
From 65 to 84 years	x	(x)	x	(x)	x	(x)	x
85 years and over	x	(x)	x	(x)	x	(x)	x

Include only existing categories for the current study in the summary table.

<u>2.2 SAP Final Version 1.1 (08.11.2022)</u>

Table of Contents

1 INTRODUCTION 98
1.1 Background and rationale 98
1.2 Study objectives 98
1.3 Study endpoints 98
2 STUDY METHODS 99
2.1 Trial design 99
2.2 Randomization 99
2.3 Sample size 99
2.4 Framework 100
2.5 Statistical interim analyses and stopping guidance 100
2.6 Timing of final analysis 100
2.7 Timing of outcome assessments 100
3 STATISTICAL PRINCIPLES 101
3.1 Confidence intervals and P values 101
3.2 General calculation rules 101
3.3 Adherence and protocol deviations 101
3.4 Analysis populations 101
3.5 Event and censor times for the Kaplan-Meier analyses 101
4 ANALYSIS 102
4.1 Trial population 102
4.1.1 Screening data 102
4.1.2 Eligibility 102
4.1.3 Recruitment 102
4.1.4 Withdrawal/follow-up 102
4.1.5 Baseline patient characteristics 103
4.2 Outcome definitions 103
4.3 Analysis methods for the primary and secondary endpoints 104
4.3.1 Primary endpoint 104
4.3.2 Supportive analysis of the primary endpoint 104
4.3.3 Secondary endpoint analysis 104
4.3.3.1 Non-infectious wound complications 105
4.3.3.2 Hospital stay 105
4.3.3.3 Thirty-day mortality 105
4.3.3.4 Re-operation rate 105
4.3.3.5 Subgroup analysis 105
4.3.3.6 Treatment costs 106
4.3.3.7 Adverse events 106

4.4 Missing data ... 106
4.5 Additional analyses ... 106
4.6 Statistical software ... 106
5 TABLE SHELLS ... 107

7 INTRODUCTION

7.1 Background and rationale

Surgical site infection (SSI) is one of the most common complications following abdominal visceral surgery and dramatically increases length of hospital stay and costs. Hypothetically, intraoperative wound irrigation (IOWI) before skin closure with saline or antiseptics might be a potential pragmatic option to reduce SSI rates. Currently, there are no official recommendations on its use and clinical practice varies largely. Solutions containing the antiseptic agent polyhexanide (PHX) are approved for IOWI, and were shown to promote wound healing, but have not been evaluated in randomized clinical trials (RCTs) in abdominal visceral surgery. Therefore, we designed a multicenter, randomized, observer-blinded clinical trial evaluating the efficacy of IOWI with PHX solution or saline before skin closure after laparotomy. The primary endpoint is the incidence of SSI 30 days postoperatively, according to the Center of Disease Control (CDC) definition. The results of the trial will provide evidence for definite clinical recommendations regarding the use of IOWI and influence current guidelines and provide all participating patients the opportunity of an improved treatment.

7.2 Study objectives

To investigate whether the use of intraoperative, epifascial wound irrigation with PHX solution can reduce surgical site infections after laparotomy for visceral surgery compared to saline irrigation or no irrigation.

7.3 Study endpoints

<u>Primary efficacy endpoint</u>:

SSI according to CDC criteria within 30 days postoperatively

<u>Secondary endpoints</u>:

- Non-infectious wound complications (e.g. seroma, hematoma, delayed healing) within 30 days postoperatively
- Duration of hospital stay
- Mortality and morbidity within 30 days postoperatively
- Incidence of reoperation within 30 days postoperatively
- Incidence of AE/SAE within 30 days postoperatively
  - Surgical complications will be additionally evaluated according to the Clavien-Dindo classification.

Pre-specified subgroup analysis by category of SSI (superficial, deep, organ space), NNIS risk score, ASA score, BMI, age, diabetes, smoking, alcohol consumption, history of SSI, history of radio-/chemotherapy, pre-operative hospital stay >2d, administration and timing of antibiotic prophylaxis, type and duration of surgery, intraoperative use of wound-edge protectors and changing of gloves, presence of an enterostomy.

8 STUDY METHODS

8.1 Trial design

This clinical trial is a prospective, randomized, controlled, observer and patient-blinded, multicenter, surgical trial according to German drug law (AMG) phase III-b, with three parallel comparison groups.

Patients are randomised to one of the following treatment arms:

Arm 1 (Intervention 1):

Irrigation of the subcutaneous tissue after closure of the abdominal fascia with 1000ml PHX solution (0.04%)

Arm 2 (Intervention 2):

Irrigation of the subcutaneous tissue after closure of the abdominal fascia with 1000ml saline solution (NaCl 0.9%)

Arm 3 (Control):

No epifascial wound irrigation

A total of 680 patients (290 patients in arm 1, 290 patients in arm 2, and 100 patients in arm 3) in up to 15 centres will be enrolled in the study with duration of 34 days per patient (up to three days prior to surgery, day of surgery, and 30 days post-surgery).

A total of 8 visits are scheduled during the study period.

8.2 Randomization

Patients are randomised blockwise ca. 3:3:1 to the treatment arms with stratification by centre and level of contamination of the surgical procedure (clean-contaminated, contaminated, or dirty) during surgery after closure of the abdominal fascia using RANDOBASE, the online-randomization tool at MSZ. RANDOBASE uses pre-defined randomization lists, which are created at IMedIS using Rancode Professional 2015. Two sets of sealed envelopes were produced: one for emergency unblinding at site, one for the same purpose at the MSZ-safety management department.

8.3 Sample size

Justification and calculation of the sample size can be found in the study protocol section 23.1.

Sample size adjustment (CSP approved Version 3 Amendment 2 02.03.2021):

Due to the unexpected high number of dropouts, the sample size was adjusted based on the changed analysis of SSI (see section 5.2.1). The sample size was calculated (Sample Size Software, Sample Size Tables, D. Machin et al., 2009) based on the primary endpoints of the study, assuming SSI rates (event of interest) of 2.2% in the PHX group (assuming a 75% risk reduction according to the trial by Roth et al. (14)), 8.7% in the saline group (according to the results of the trial by Cervantes-Sanchez et al. (13)), and 16.2% in the control group according to results of the previously conducted meta-analysis (12). The incidence rate of SSI over all study arms is then expected to be 7%, given the approximate 3:3:1 group assignment. The actual SSI rate up to now is 7.2%, which is very close to our assumption and we consider it valid. The

incidence rate for the competing risks of death or re-laparotomy is estimated to be a total of 13.4% in all arms. This estimation is done based on the data collected up to now.

The global significance level was set to 5% (two-sided tests). Since the PHX arm will be used twice for a comparison, the Bonferroni-Holm procedure was used to set the local alpha level for test 1 (PHX vs. no intervention) to 2.5% and for test 2 (PHX vs. saline irrigation) to 5%. If 290 patients are recruited in the PHX arm, 290 patients in the saline arm and 100 patients in the control arm (a total of 680 patients), the two Fine and Gray sub-distributional hazard models will have a power of 80% each to detect differences between the treatments.

8.4 Framework

This study tests for superiority of (1) PHX over no intervention and (2) PHX over saline irrigation with respect to SSI rate within 30 days postoperatively.

8.5 Statistical interim analyses and stopping guidance

No interim analyses are planned for this study.

8.6 Timing of final analysis

The final analysis will be performed collectively at the end of the study.

8.7 Timing of outcome assessments

Baseline data will be collected at visit 1 and 2, which means up to day 0**.**

Day 0 is defined as the time of closure of the abdominal fascia.

All other day definitions are relative to day 0.

The primary outcome will be accessed up to visit 8, which means on day 30 and up to day 36 when the time window is considered.

Visit	Day	Time window
1	-3 to -1	Up to day 0
2	0 (surgery)	
3	2	
4	4	
5	6	
6	8	
7	10	Up to day 14
8	30	Up to day 36

9 STATISTICAL PRINCIPLES

9.1 Confidence intervals and P values

All statistical tests will be performed two-sided at the global significance level of 5%. Adjustment for multiplicity will be done for the primary endpoint only, where the Bonferroni-Holm adjustment method will be used (see also section 5.2.1).

Confidence intervals will be two-sided and 95%.

9.2 General calculation rules

4. Percentages will always be quoted using number of 'known' values in the denominator unless otherwise stated.

5. P-values will be quoted to three decimal places only. Confidence intervals will also be quoted to three decimal places. However, if the statistical software SAS, which is used for analysis, prints four decimal places, values will not be rounded again, but printed to four decimal places.

6. Chi-square tests in contingency tables will be replaced by Fisher's exact tests if any expected cell frequency is less than five.

9.3 Adherence and protocol deviations

The number and percent of patients per treatment group who received IOWI will be reported.

Major protocol deviations will be listed per patient.

9.4 Analysis populations

The **Intention-to-Treat (ITT) population** will contain all randomised patients with results attributed to the treatment group they were randomised to.

The **safety analysis (SA) population** will consist of all randomised subjects with results attributed to the treatment group of their actual treatment.

Safety analysis will be performed on the SA population. All other analyses will be performed in the ITT population.

9.5 Event and censor times for the Kaplan-Meier analyses

The following definitions will apply to the time-to-event analysis of SSI:

**Parameter:**	SSI
**Main Event Time**	time of SSI
**Competing Event Times**	time of death<br>time of re-laparotomy
**Censor Time**	time of last follow-up*

* for patients without SSI

10 ANALYSIS

10.1 Trial population

10.1.1 Screening data

The overall number of screened patients will be presented in the report.

The reasons for non-eligibility will also be summarized.

10.1.2 Eligibility

Key inclusion criteria:

- Clean-contaminated, contaminated or dirty (according to CDC classification)
- Abdominal surgery by midline or transverse laparotomy (elective or emergency)
- Age ≥18 years
- American Society of Anesthesiologists (ASA) score ≤ 3

Key exclusion criteria:

- Pregnancy or breast feeding
- Known hypersensitivity/allergy to PHX
- Inability to understand/give informed consent
- Inability to attend follow-up visits
- Revision-surgery (previous abdominal surgery within the last 30 days)
- Planned re-laparotomy within 30 days
- Severe immunosuppression
- Concurrent abdominal wall infections
- Pre-operative antibiotic therapy (within 5 days prior to surgery)

The full set of inclusion and exclusion criteria can be found in the study protocol sections 12.1 and 12.2.

10.1.3 Recruitment

Tables will contain the following absolute and relative frequencies per treatment group and overall:

- patients who entered the study.
- patients within each analysis set including reasons for exclusion. Patients in the SA will be considered in the group of their actual treatment.
- patients per centre on the ITT set.

10.1.4 Withdrawal/follow-up

Since study treatment is given only once at the beginning of the study, withdrawal from study treatment is not of interest.

The number of patients per treatment group who did not complete the study will be given in a summary table by reason (multiple reasons possible) including absolute and relative frequencies.

10.1.5 Baseline patient characteristics

The following characteristics will be summarized per treatment group on the ITT set:

Demographics: sex, age, BMI.

Medical history: main diagnosis leading to operation (malign/benign), ASA classification, diabetes (including type and treatment), allergies, comorbidities (11 pre-defined and other; multiple comorbidities are possible), previous abdominal surgery (no, single, multiple), time since last abdominal surgery, history of SSI (no, single, multiple), time since last SSI, location of last SSI, history of radiotherapy (no, single, multiple), time since end of last radiotherapy, dose (Gy), history of chemotherapy (no, single, multiple), smoking (no, previously, currently), packyears, regular alcohol consumption (no, previously, currently), glasses/week.

Surgery: duration of preoperative hospital stay (days), urgency and type of procedure, duration of surgery (min), antibiotic prophylaxis (no, yes >1h prior OP, yes ≤1h prior OP), type of skin disinfectant, type of incision, length of incision, intra-OP change of gloves (y/n), intra-OP use of wound edge protectors (y/n), enterostomy created (y/n), type of abdominal fascia closure, use of mesh (no, yes-sublay, yes-onlay), level of contamination (class I to IV), NNIS risk score, wound closure (complete/incomplete), subcutaneous sutures used (y/n), skin closure type (stapler, continuous suture, single suture).

Absolute and relative frequencies will be presented for categorical variables. Number of valid cases, mean, standard deviation, median, minimum, and maximum will be displayed for continuous variables.

10.2 Outcome definitions

The primary endpoint is the frequency of physician-assessed SSI up to 30 days post-surgery. According to the time-definitions, the SSI assessment may take place up to day 36. Surgical site infections will additionally be classified in three groups: superficial incisional, deep incisional, and organ/space.

The secondary endpoints of this study are:

Frequency of non-infectious wound complications (e.g. seroma, hematoma, delayed healing) within 30 days postoperatively;

Duration of hospital stay;

Mortality within 30 days postoperatively;

Rate of reoperation within 30 days post-surgery, which will be identified during medical review based on the AE records.

Pre-specified subgroup analysis by category of SSI (superficial, deep, organ space), NNIS risk score, ASA score, BMI, age, diabetes, smoking, alcohol consumption, history of SSI, history of radio-/chemotherapy, pre-operative hospital stay >2d, administration and timing of antibiotic prophylaxis, type and duration of surgery, intraoperative use of wound-edge protectors and changing of gloves, presence of an enterostomy.

Assessment of safety:

The assessment of safety will be based on the frequency of AE/SAE other than SSI within the safety population (according to CTCAE V. 4.3). Surgical complications will be additionally evaluated according to the Clavien-Dindo classification.

10.3 Analysis methods for the primary and secondary endpoints

10.3.1 Primary endpoint

Wound irrigation with PHX solution will be tested for superiority over no irrigation (Test 1) and irrigation with saline (Test 2) with respect to the incidence of SSI within 30 days of surgery using two Fine and Gray sub-distributional hazard models with SSI as main event and re-laparotomy and death as competing risks. The model actually compares time to event, although the timing of SSI is of less interest than the occurrence of SSI within 30 days of surgery.

Since randomization is stratified by study center and level of contamination, the models were planned to include covariates study center and level of contamination in addition to treatment group. After reviewing the blinded data, it was decided to combine contamination classes I with II and III with IV. Classes I and IV contain two patients each. Since there are small study centers which would not allow the model to converge, center will not be used as covariate (Langen N=5, Düsseldorf N=6).

The global significance level is set to 5%. Using the Bonferroni-Holm adjustment, the local significance level will be 2.5% and 5% in the order of increasing p-value.

10.3.2 Supportive analysis of the primary endpoint

In case there are differences between the treatment groups in terms of baseline characteristics, those will also be included as covariates in the models.

Type and duration of operation, use of wound-edge protectors, intraoperative changing of gloves and patient related risk factors (NNIS risk score, BMI, age, diabetes) might influence the outcome, which is why they will also be included as model covariates.

Descriptive statistics for the primary endpoint will be presented per treatment group: absolute and relative frequencies of

- SSI (overall and by class: superficial incisional, deep incisional, and organ/space)
- Re-laparotomy (will be identified during medical review based on the AE records)
- Death
- Lost to follow-up
- Completed study without event

Those incidences will be compared between treatment groups using Fisher’s exact test in order to better understand the distribution of missing values.

10.3.3 Secondary endpoint analysis

Secondary endpoints will be analysed by study group on the ITT set using appropriate descriptive statistics. Any explorative statistical testing will be performed two-sided using a significance level of 5%.

10.3.3.1 Non-infectious wound complications

If wound complications are recorded on the same day as an SSI or an ongoing SSI, then they will not be included in the analysis, as they are not non-infectious (and are therefore already accounted for in the SSI analysis).

Non-infectious wound complications (seroma, hematoma, delayed healing, necrosis) within 30 days postoperatively will be summarized by treatment group using absolute and relative frequencies. The two types of irrigation will be compared to no irrigation using the Fisher exact test.

10.3.3.2 Hospital stay

Duration of hospital stay – overall and post-surgery. The later will be calculated by subtracting the duration of preoperative hospital stay from the (overall) duration of hospital stay, recorded at study end (visit 8). All durations will be measured in days. Number of valid cases, mean, standard deviation, median, minimum, and maximum of post-surgical hospital stay will be displayed per treatment group. The two types of irrigation will be compared to no irrigation using the Mann-Whitney-U test.

10.3.3.3 Thirty-day mortality

Mortality within 30 days postoperatively will be summarized by treatment group using absolute and relative frequencies. The two types of irrigation will be compared to no irrigation using the Fisher exact test.

10.3.3.4 Re-operation rate

Rate of reoperation within 30 days post-surgery will be identified during medical review based on the AE records and will be summarized by treatment group using absolute and relative frequencies. The two types of irrigation will be compared to no irrigation using the χ^2-test.

10.3.3.5 Subgroup analysis

The primary and secondary endpoints will be additionally summarized within the following subgroups:

- Type of SSI (superficial, deep, organ space)
- NNIS - surgical infection risk index (0, 1, 2, 3)
- ASA score (1, 2, 3)
- BMI (<18.5, 18.5≤BMI<25, 25≤BMI<30, ≥30)
- Age (18≤age<40, 40≤age<65, 65≤age<85, ≥85)
- Diabetes (Type I, Type II - insulin, Type II - oral antidiabetics, Type II - dietary)
- Smoker (current, former, no)
- Alcohol consumption (current, former, no)
- History of SSI (multiple, single, no)
- History of radiotherapy (multiple, single, no)
- History of chemotherapy (multiple, single, no)

- Preoperative hospital stay >2d (y/n)
- Antibiotic prophylaxis (yes >1h prior OP, yes ≤1h prior OP, no)
- Type of surgery (intestinal, hepato-biliary, other):
  1 = Colo-rectal= colon, rectum, appendix
  2 = Hepato-bilary= pancreas, bile duct, hepatic
  3 = Oesophago-gastric = esophageal and gastric
  4 = Other
- Duration of surgery taken from NNIS: Length of OP >T hours (y/n)
- Intraoperative use of wound-edge protectors (y/n)
- Intraoperative changing of gloves (y/n)
- Presence of an enterostomy (y/n)
- Level of contamination (Class II, III, IV)

Absolute and relative frequencies will be presented for categorical variables. Number of valid cases, mean, standard deviation, median, minimum, and maximum will be displayed for continuous variables. Tests will only be performed in case clinically meaningful differences are observed between treatment groups.

10.3.3.6 Treatment costs

Analysis of treatment costs will be done indirectly through the between-group comparisons of hospital stay and surgical complications. This analysis is already described in sections 4.3.3.1, 4.3.3.2, 4.3.3.4, 4.3.1 and 4.3.2.

10.3.3.7 Adverse events

All AEs other than SSI will be analysed on the safety set. The overall incidence of non-SAE-adverse events, SAEs, and related SAEs will be tabulated using MedDRA System Organ Class and Preferred Term by treatment group (see table shells). The overall occurrences as well as the number of affected patients are of interest. SAEs and related SAEs which resulted in death will also be tabulated.

10.4 Missing data

Missing primary endpoint data in the primary analysis will be dealt with using competing risks and censoring. Missing SSI evaluation due to death or re-laparotomy will be considered a competing risk. Missing SSI for all other reasons will be censored. Data will not be imputed for other analyses such as secondary or subgroup analyses.

10.5 Additional analyses

The following parameters will be summarized by treatment group using absolute and relative frequencies: use of concomitant medications of special interest (antibiotics, immunosuppression, anticoagulants), SSI (no/yes-new/yes-ongoing), Type of SSI (including their Clavien-Dindo classification), non-SSI wound complications (seroma, hematoma, delayed healing, necrosis, other wound intervention (none, VAC, bedside wound revision, re-operation, other), abnormal lab values.

10.6 Statistical software

Analysis will be performed with SAS version 9.4.

11 TABLE SHELLS

SAE, related-SAE, SAE resulting in death, related-SAE resulting in death, and non-SAE-AEs (5 tables)

System Organ Class<br>Preferred Term	Exposed to Treatment 1<br>N=			Exposed to Treatment 2<br>N=			Exposed to Treatment 3<br>N=		
	Events	Subjects affected		Events	Subjects affected		Events	Subjects affected	
		n	%*		n	%*		n	%*
OVERALL	x	x	(x)	x	x	(x)	x	x	(x)
SOC1	x	x	(x)	x	x	(x)	x	x	(x)
PT1	x	x	(x)	x	x	(x)	x	x	(x)
PT2	x	x	(x)	x	x	(x)	x	x	(x)
PT3	x	x	(x)	x	x	(x)	x	x	(x)
SOC2	x	x	(x)	x	x	(x)	x	x	(x)
PT4	x	x	(x)	x	x	(x)	x	x	(x)
PT5	x	x	(x)	x	x	(x)	x	x	(x)
…									

** with respect to the number of exposed subjects*

Subjects enrolled per age group

Age group	Treatment 1		Treatment 2		Treatment 3		Total
	n	%	n	%	n	%	n
Total	x	(x)	x	(x)	x	(x)	x
Adults (18-64 years)	x	(x)	x	(x)	x	(x)	x
From 65 to 84 years	x	(x)	x	(x)	x	(x)	x
85 years and over	x	(x)	x	(x)	x	(x)	x

2.3 SAP Summary of Changes

Due to the large number of missing data that the unexpected high number of drop-outs would have meant, we adapted the analysis strategy, so the primary endpoint was analysed using the Fine and Gray sub-distributional hazard models with SSI as main event and relaparotomy and death as competing risks. The rest of the missing SSI was censored at time of last follow-up. The sample size was adjusted based on this changed analysis of SSI. The sample size was calculated based on the primary endpoints of the study, assuming SSI rates (event of interest) of 2.2% in the PHX group (assuming a 75% risk reduction according to the trial by Roth et al.), 8.7% in the saline group (according to the results of the trial by Cervantes-Sanchez et al.), and 16.2% in the control group according to results of the previously conducted meta-analysis. The incidence rate of SSI over all study arms was then expected to be 7%, given the approximate 3:3:1 group assignment. The incidence rate for the competing risks of death or re-laparotomy was estimated to be a total of 13.4% in all arms. This estimation was done based on the data collected up to that timepoint. The global significance level was set to 5% (two-sided tests). Since the PHX arm was used twice for a comparison, the Bonferroni-Holm procedure was used to set the local alpha level for test 1 (PHX vs. no intervention) to 2.5% and for test 2 (PHX vs. saline irrigation) to 5%. If 290 patients were recruited in the PHX arm, 290 patients in the saline arm and 100 patients in the control arm (a total of 680 patients), the two Fine and Gray sub-distributional hazard models had a power of 80% each to detect differences between the treatments. These changes were submitted to and approved by the ethics committee and health authority (BfARM) as a second amendment of the study protocol on the 8th and 24th March 2021 respectively. No other changes were made in the trial conduct in respect to the original design.
