# Supplementary material for: Intraoperative Wound Irrigation for the Prevention of Surgical Site Infection After Laparotomy: A Randomized Clinical Trial by CHIR-Net
Source: JAMA Surg. 2024 Feb 21;159(5):484–92. doi: 10.1001/jamasurg.2023.7985 (PMC10882507; doi:10.1001/jamasurg.2023.7985)
Supplement: Supplement 2. — eAppendix 1. CDC classification of intraoperative Level of Contamination (LOC) eAppendix. 2. CDC classification of Surgical Site Infection (SSI) eTable 1. Sensitivity analysis of the primary endpoint with the subdistributional hazard model of Fine and Gray with SSI as main event, re-laparotomy and death as competing risks, treatment group, and several patient characteristics as covariates eTable 2. Analysis of the primary endpoint with the subdistributional hazard model of Fine and Gray with SSI as main event and relaparotomy and death as competing risks, and treatment group, level of contamination, BMI class, cardiovascular, and pulmonary comorbidities as covariates eTable 3. Frequency, severity, causal relationship, and types of serious adverse events for the wound irrigation groups PHX, saline, and no irrigation eTable 4. Clavien‒Dindo classification of surgical complications (including SSI I-III) in the IOWISI trial by study group [file jamasurg-e237985-s002.pdf]

Supplemental Online Content

Mueller TC, Kehl V, Dimpel R, et al; the IOWISI study group. Intraoperative wound irrigation for the prevention of surgical site infection after laparotomy: a randomized clinical trial by CHIR-Net. *JAMA Surg*. Published online February 21, 2024. doi:10.1001/jamasurg.2023.7985

eAppendix 1. CDC classification of intraoperative Level of Contamination (LOC)

eAppendix. 2. CDC classification of Surgical Site Infection (SSI)

eTable 1. Sensitivity analysis of the primary endpoint with the subdistributional hazard model of Fine and Gray with SSI as main event, re-laparotomy and death as competing risks, treatment group, and several patient characteristics as covariates

eTable 2. Analysis of the primary endpoint with the subdistributional hazard model of Fine and Gray with SSI as main event and relaparotomy and death as competing risks, and treatment group, level of contamination, BMI class, cardiovascular, and pulmonary comorbidities as covariates

eTable 3. Frequency, severity, causal relationship, and types of serious adverse events for the wound irrigation groups PHX, saline, and no irrigation

eTable 4. Clavien–Dindo classification of surgical complications (including SSI I-III) in the IOWISI trial by study group

This supplemental material has been provided by the authors to give readers additional information about their work.

© 2024 Mueller TC et al. *JAMA Surgery.*

eAppendix. 1 CDC classification of intraoperative Level of Contamination (LOC)

LOC	Criteria	SSI rates*
**Level I/ Clean**	-uninfected operative wounds<br>-no inflammation is encountered<br>-the respiratory, alimentary, genital, or uninfected urinary tracts are **not** entered	1-5%
**Level II/ Clean-contaminated**	-the respiratory, alimentary, genital, or urinary tracts are entered under controlled conditions and without unusual contamination<br>-no evidence of infection or major break in technique is encountered	3-11%
**Level III/ Contaminated**	-operations with major breaks in sterile technique or gross spillage from gastrointestinal tract<br>-incisions in which acute, non-purulent inflammation is encountered<br>-outside object had contact with wound (e.g., bullet, knife blade)	10-17%
**Level IV/ Dirty-infected**	- existing clinical infection<br>-perforated viscera<br>-foreign object lodged in wound<br>-any wound that has been exposed to pus or fecal matter	>27%

**according to Reeves N, Torkington J. Prevention of surgical site infections. Surgery (Oxford). 2022;40(1):20-4.*

© 2024 Mueller TC et al. *JAMA Surgery.*

eAppendix. 2 CDC classification of Surgical Site Infection (SSI)

	Infection occurs within 30 days after the operation …
**Superficial incisional SSI (class I)**	**…and** infection involves **only skin or subcutaneous tissue** **and** at least ***one*** of the following:<br><br>1 Purulent drainage from the superficial incision<br>2 Organisms isolated from an aseptically obtained culture of fluid or tissue from the superficial incision<br>3 At least one of the following signs or symptoms of infection: pain or tenderness, localized swelling, redness, or heat *and* superficial incision is deliberately opened by surgeon, *unless* incision is culture negative
**Deep incisional SSI (class II)**	**…and** infection involves **deep soft tissues (fascial and muscle layers)** **and** at least ***one*** of the following:<br><br>1 Purulent drainage from the deep incision but not from the organ/space component of the surgical site<br>2 A deep incision spontaneously dehisces or is deliberately opened by a surgeon<br>3 And at least one of the following symptoms: fever (>38°C), localized pain, or tenderness of the incision area *unless* incision is culture negative<br>4 An abscess or other evidence of infection involving the deep incision is found on direct examination, during reoperation, or by histopathologic or radiologic examination
**Organ/space SSI (class III)**	…**and** infection involves any part of the anatomy (*e.g.,* organs or spaces), other than the incision, which was opened or manipulated during an operation **and** at least ***one*** of the following:<br><br>1 Purulent drainage from a drain that is placed through a stab wound into the organ/space<br>2 Organisms isolated from an aseptically obtained culture of fluid or tissue in the organ/space
	**…or the infection is diagnosed by the attending surgeon**

© 2024 Mueller TC et al. *JAMA Surgery.*

eTable 1. Sensitivity analysis of the primary endpoint with the subdistributional hazard model of Fine and Gray with SSI as main event, re-laparotomy and death as competing risks, treatment group, and several patient characteristics as covariates

		95%CI for HR		
	HR	*Lower*	*Upper*	*P-value*
Treatment group				0·833
(Test 1) No irrigation vs. PHX	1·123	0·579	2·175	0·732
(Test 2) Saline vs. PHX	1·167	0·701	1·945	0·552
Level of contamination				
III/IV vs. I/II	1·396	0·565	3·450	0·469
BMI class				0·780
18·5 ≤ BMI < 25 (vs. BMI < 18·5)	1·339	0·371	4·830	0·656
25 ≤ BMI < 30 (vs. BMI < 18·5)	1·075	0·290	3·985	0·914
30 ≤ BMI (vs. BMI < 18·5)	1·022	0·260	4·012	0·975
Diabetes				
Yes vs. no	0·836	0·449	1·556	0·572
NNIS risk score	1·019	0·735	1·413	0·910
Type of surgery				0·267
HBP vs. colorectal	0·564	0·284	1·121	0·102
Upper GI vs. colorectal	0·559	0·253	1·235	0·151
Other vs. colorectal	0·284	0·037	2·148	0·223
Duration of surgery (min)	1·001	0·999	1·003	0·205
Intraoperative change of gloves				
Yes vs. no	1·076	0·637	1·818	0·783
Use of wound edge protectors				
Yes vs. no	1·094	0·635	1·887	0·746
Enterostomy created				
Yes vs. no	1·975	0·913	4·274	0·084

Abbreviations: PHX = Polyhexanide; BMI = body mass index; CI = Confidence interval; HR = Hazard ratio; HBP = hepatobiliary-pancreatic, GI = Gastrointestinal, NNIS = National Nosocomial Infections Surveillance

© 2024 Mueller TC et al. *JAMA Surgery.*

eTable 2. Analysis of the primary endpoint with the subdistributional hazard model of Fine and Gray with SSI as main event and relaparotomy and death as competing risks, and treatment group, level of contamination, BMI class, cardiovascular, and pulmonary comorbidities as covariates

		95%CI for HR		*P-value*
	HR	*Lower*	*Upper*	
Treatment group				0·714
(Test 1) No irrigation vs. PHX	1·236	0·635	2·404	0·533
(Test 2) Saline vs. PHX	1·201	0·740	1·950	0·458
Level of contamination				
III/IV vs. I/II	1·660	0·772	3·571	0·194
BMI class				0·829
18·5 ≤ BMI < 25 (vs. BMI < 18·5)	1·456	0·367	5·771	0·593
25 ≤ BMI < 30 (vs. BMI < 18·5)	1·197	0·305	4·699	0·797
30 ≤ BMI (vs. BMI < 18·5)	1·170	0·285	4·797	0·827
Cardiovascular comorbidity				0·562
Current (vs. none)	1·007	0·651	1·559	0·975
Previous (vs. none)	0·343	0·048	2·452	0·286
Pulmonary comorbidity				0·145
Current (vs. none)	0·829	0·385	1·782	0·630
Previous (vs. none)	2·135	0·966	4·718	0·061

Abbreviations: PHX = Polyhexanide; BMI = body mass index; CI = Confidence interval; HR = Hazard ratio

© 2024 Mueller TC et al. *JAMA Surgery.*

eTable 3. Frequency, severity, causal relationship, and types of serious adverse events for the wound irrigation groups PHX, saline, and no irrigation

	Exposed to PHX n=291			*Exposed to Saline n=295*			*Exposed to No irrigation n=103*			*P-value* &
		Subjects affected			*Subjects affected*			*Subjects affected*		
	Events	*n*	*(%)*	*Events*	*n*	*(%)*	*Events*	*n*	*(%)*	
SAE	178 (100)	95	(33)	177 (100)	104	(35)	63 (100)	41	(40)	0·232
Severity (n, % #)										0·031
Mild	17 (9·6)	··	··	9 (5·1)	··	··	7 (11·1)	··	··	··
Moderate	48 (27·0)	··	··	54 (30·5)	··	··	14 (22·2)	··	··	··
Severe	58 (32·6)	··	··	65 (36·7)	··	··	28 (44·4)	··	··	··
Life-threatening	47 (26·4)	··	··	32 (18·1)	··	··	8 (12·7)	··	··	··
Death	8 (4·5)	··	··	17 (9·6)	··	··	6 (9·5)	··	··	··
Causal relationship (n, % #)										0·709 §
Unrelated	167 (93·8)	··	··	165 (93·2)	··	··	63 (100·0)	··	··	··
Unlikely related	11 (6·1)	··	··	11 (6·2)	··	··	0	··	··	··
Probably related	0	··	··	1 (0·6)	··	··	0	··	··	··
SAE specification*										
Cardiac disorder	6	5	(2)	6	4	(1)	3	3	(3)	0·583
Respiratory, thoracic, or mediastinal disorder	16	12	(4)	12	11	(4)	4	3	(3)	0·856
Vascular disorder	10	10	(3)	12	9	(3)	3	3	(3)	0·951
Surgically relevant	6	6	(2)	10	8	(3)	3	3	(3)	0·835
Renal or urinary disorder	3	3	(1)	2	2	(1)	2	2	(2)	0·553
Gastrointestinal disorder	30	24	(8)	32	31	(11)	14	14	(14)	0·279
Surgically relevant	15	13	(4)	17	17	(6)	8	8	(8)	0·419
Intra-abdominal hemorrhage/hematoma	5	5	(2)	3	3	(1)	4	4	(4)	0·158
Hepatobiliary disorder	10	10	(3)	18	13	(4)	4	4	(4)	0·815
Surgically relevant	5	5	(2)	0	0		1	1	(1)	0·222
Infection or infestation	24	24	(8)	26	24	(8)	6	6	(6)	0·712
Surgically relevant	11	11	(4)	17	17	(6)	5	5	(5)	0·494

© 2024 Mueller TC et al. *JAMA Surgery.*

Injury, poisoning, or procedural complications	30	26	(9)	31	30	(10)	14	13	(13)	0·560
Surgically relevant	28	25	(9)	29	28	(9)	14	13	(13)	0·489
Anastomotic complications	14	14	(5)	15	15	(5)	10	10	(10)	0·154
Wound complications	10	9	(3)	11	10	(3)	3	3	(3)	0·966
Other	49	32	(11)	38	28	(9)	13	10	(10)	0·823

Symbols: & χ² test over all groups, unless otherwise stated; § Fisher exact test comparing all groups; * coded with MedDRA; # Number of SAE in the denominator

Abbreviations: PHX = Polyhexanide; SAE = Serious Adverse Event

© 2024 Mueller TC et al. *JAMA Surgery.*

eTable 4. Clavien–Dindo classification of surgical complications (including SSI I-III) in the IOWISI trial by study group

		Treatment group					
		PHX		Saline		No irrigation	
		N	%	N	%	N	%
Clavien–Dindo classification	Grade I	88	40,4%	84	35,1%	34	45,3%
	Grade II	38	17,4%	36	15,1%	7	9,3%
	Grade IIIa	52	23,9%	64	26,8%	16	21,3%
	Grade IIIb	37	17,0%	41	17,2%	16	21,3%
	Grade IVa	1	0,5%	3	1,3%	0	
	Grade IVb	1	0,5%	4	1,7%	0	
	Grade V	1	0,5%	7	2,9%	2	2,7%

Abbreviations: PHX = Polyhexanide

© 2024 Mueller TC et al. *JAMA Surgery.*
