# Supplementary material for: Intraoperative Wound Irrigation for the Prevention of Surgical Site Infection After Laparotomy: A Randomized Clinical Trial by CHIR-Net
Source: JAMA Surg. 2024 Feb 21;159(5):484–92. doi: 10.1001/jamasurg.2023.7985 (PMC10882507; doi:10.1001/jamasurg.2023.7985)
Supplement: Supplement 3. — IOWISI study group members [file jamasurg-e237985-s003.pdf]

*Indicates required information. Only first name, last name, and suffix will appear in PubMed.

*Group Name(s): IOWISI Study Group

*First Name and Middle Initial(s)	*Last Name	*Suffix (eg, Jr, III)	Academic Degrees	Institution	Location (city, state/province, country)	Role or Contribution, eg, chair, principal investigator	Group (if more than 1 Group listed in the byline) and/or Subgroup (eg, Steering Committee)
Edith	Heimsch			Münchner Studienzentrum, Technical University of Munich, School of Medicine and Health, Ismaninger Str. 22, 81675 Munich, Germany	Munich, Germany	Data Management	
Alfred	Zollner			Münchner Studienzentrum, Technical University of Munich, School of Medicine and Health, Ismaninger Str. 22, 81675 Munich, Germany	Munich, Germany	Safety Management	
Beate	Schossow			Münchner Studienzentrum, Technical University of Munich, School of Medicine and Health, Ismaninger Str. 22, 81675 Munich, Germany	Munich, Germany	Project Assistant	
Petra	Wagner			Münchner Studienzentrum, Technical University of Munich, School of Medicine and Health, Ismaninger Str. 22, 81675 Munich, Germany	Munich, Germany	Project Assistant	
Urula	Böcher			Technical University of Munich, School of Medicine and Health, Department of Surgery, Ismaninger Str. 22, 81675 Munich, Germany	Munich, Germany	Study Nurse	
Dorothea	Dichtl			Technical University of Munich, School of Medicine and Health, Department of Surgery, Ismaninger Str. 22, 81675 Munich, Germany	Munich, Germany	Study Nurse	
Rüdiger	Göß		MD	Technical University of Munich, School of Medicine and Health, Department of Surgery, Ismaninger Str. 22, 81675 Munich, Germany	Munich, Germany	Clinical Investigator	
Carmen	Mota-Reyes		MD	Technical University of Munich, School of Medicine and Health, Department of Surgery, Ismaninger Str. 22, 81675 Munich, Germany	Munich, Germany	Clinical Investigator	
Ilaria	Pergolini		MD	Technical University of Munich, School of Medicine and Health, Department of Surgery, Ismaninger Str. 22, 81675 Munich, Germany	Munich, Germany	Clinical Investigator	
Stephan	Schorn		MD	Technical University of Munich, School of Medicine and Health, Department of Surgery, Ismaninger Str. 22, 81675 Munich, Germany	Munich, Germany	Clinical Investigator	
Matthias	Kelm		MD	Department of General, Visceral, Transplant, Vascular and Pediatric Surgery, University Hospital of Würzburg, 97080 Würzburg, Germany	Wuerzburg, Germany	Clinical Investigator	
Franziska	Köhler		MD	Department of General, Visceral, Transplant, Vascular and Pediatric Surgery, University Hospital of Würzburg, 97080 Würzburg, Germany	Wuerzburg, Germany	Clinical Investigator	
Sophie	Müller		MD	Department of General, Visceral, Transplant, Vascular and Pediatric Surgery, University Hospital of Würzburg, 97080 Würzburg, Germany	Wuerzburg, Germany	Clinical Investigator	
Agnes	Treutlein		MD	Department of General, Visceral, Transplant, Vascular and Pediatric Surgery, University Hospital of Würzburg, 97080 Würzburg, Germany	Wuerzburg, Germany	Clinical Investigator	
Anna	Widder		MD	Department of General, Visceral, Transplant, Vascular and Pediatric Surgery, University Hospital of Würzburg, 97080 Würzburg, Germany	Wuerzburg, Germany	Clinical Investigator	
Ulrike	Wulfsteiner		MD	Department of General, Visceral, Transplant, Vascular and Pediatric Surgery, University Hospital of Würzburg, 97080 Würzburg, Germany	Wuerzburg, Germany	Clinical Investigator	
Laura Isabel	Hanke		MD	Department of General, Visceral and Transplant Surgery, University Medical Center Mainz, Langenbeckstr. 1, 55131 Mainz, Germany	Mainz, Germany	Clinical Investigator	
Nicolas	Wachter		MD	Department of General, Visceral and Transplant Surgery, University Medical Center Mainz, Langenbeckstr. 1, 55131 Mainz, Germany	Mainz, Germany	Clinical Investigator	
Christian	Boedecker		MD	Department of General, Visceral and Transplant Surgery, University Medical Center Mainz, Langenbeckstr. 1, 55131 Mainz, Germany	Mainz, Germany	Clinical Investigator	
Elnaz	Payani			Department of General, Visceral and Transplantation Surgery, Ludwig Maximilians University of Munich, University Hospital, Marchioninistr. 15, 81377 Munich, Germany		Clinical Investigator	
Alena	Sint			Department of General, Visceral and Transplantation Surgery, Ludwig Maximilians University of Munich, University Hospital, Marchioninistr. 15, 81377 Munich, German	Munich, Germany	Clinical Investigator	
Nicole	Trebesius			Department of General, Visceral and Transplantation Surgery, Ludwig Maximilians University of Munich, University Hospital, Marchioninistr. 15, 81377 Munich, German	Munich, Germany	Clinical Investigator	
Simon	Weigand			Department of General, Visceral and Transplantation Surgery, Ludwig Maximilians University of Munich, University Hospital, Marchioninistr. 15, 81377 Munich, German	Munich, Germany	Clinical Investigator	
Katharina	Brüwer			Department of General, Visceral and Transplantation Surgery, Ludwig Maximilians University of Munich, University Hospital, Marchioninistr. 15, 81377 Munich, German	Munich, Germany	Clinical Investigator	
Barbara	Maichle			Clinical Study Center, Department of General, Visceral and Transplantation Surgery, Heidelberg University Hospital, Im Neuenheimer Feld 420, 69120, Heidelberg, Ge	Heidelberg, Germany	Clinical Investigator	
Britta	Knoth			Clinical Study Center, Department of General, Visceral and Transplantation Surgery, Heidelberg University Hospital, Im Neuenheimer Feld 420, 69120, Heidelberg, Ge	Heidelberg, Germany	Study Coordinator	
Katharina	Hiller			Clinical Study Center, Department of General, Visceral and Transplantation Surgery, Heidelberg University Hospital, Im Neuenheimer Feld 420, 69120, Heidelberg, Ge	Heidelberg, Germany	Study Nurse	
Tim	Fahlbusch		MD	Department of General and Visceral Surgery, St. Josef Hospital, Ruhr University Bochum Hospitals, Gudrunstr. 56, 44791 Bochum, Germany	Bochum, Germany	Clinical Investigator	
Richard	Hummel		MD	Department of Surgery, University Medical Center Schleswig-Holstein, Campus Lübeck, Ratzeburger Allee 160, 23538 Lübeck, Germany	Luebeck, Germany	Clinical Investigator	
Ulrich	Wellner		MD	Department of Surgery, University Medical Center Schleswig-Holstein, Campus Lübeck, Ratzeburger Allee 160, 23538 Lübeck, Germany	Luebeck, Germany	Clinical Investigator	
Julia	Bertram				Luebeck, Germany	Study Nurse	
Jürgen	Weitz		MD	Department of Visceral, Thoracic and Vascular Surgery, Faculty of Medicine and University Hospital Carl Gustav Carus, Technische Universität Dresden, Fetscherstr. 74, 01307 Dresden, Germany; & National Center for Tumor Diseases (NCT/UCC), Dresden, Germany: German Cancer Research Center (DKFZ), Heidelberg; Faculty of Medicine and University Hospital Carl Gustav Carus, Technical University of Dresden, Helmholtz Center Dresden-Rossendorf (HZDR), Dresden, Germany	Dresden, Germany	Clinical Investigator	
Sandra	Korn		PhD	Department of Visceral, Thoracic and Vascular Surgery, Faculty of Medicine and University Hospital Carl Gustav Carus, Technische Universität Dresden, Fetscherstr. 74, 01307 Dresden, Germany; & National Center for Tumor Diseases (NCT/UCC), Dresden, Germany: German Cancer Research Center (DKFZ), Heidelberg; Faculty of Medicine and University Hospital Carl Gustav Carus, Technical University of Dresden, Helmholtz Center Dresden-Rossendorf (HZDR), Dresden, Germany	Dresden, Germany		
Janusz von	Renesse		MD	Department of Visceral, Thoracic and Vascular Surgery, Faculty of Medicine and University Hospital Carl Gustav Carus, Technische Universität Dresden, Fetscherstr. 74, 01307 Dresden, Germany; & National Center for Tumor Diseases (NCT/UCC), Dresden, Germany: German Cancer Research Center (DKFZ), Heidelberg; Faculty of Medicine and University Hospital Carl Gustav Carus, Technical University of Dresden, Helmholtz Center Dresden-Rossendorf (HZDR), Dresden, Germany	Dresden, Germany	Clinical Investigator	
Karl	Karstens		MD	Department of General, Visceral and Thoracic Surgery, University Hospital Hamburg-Eppendorf, Martinistr. 52, 20246 Hamburg, Germany	Hamburg, Germany	Clinical Investigator	
Nathaniel	Melling		MD	Department of General, Visceral and Thoracic Surgery, University Hospital Hamburg-Eppendorf, Martinistr. 52, 20246 Hamburg, Germany	Hamburg, Germany	Clinical Investigator	
Matthias	Reeh		MD	Department of General, Visceral and Thoracic Surgery, University Hospital Hamburg-Eppendorf, Martinistr. 52, 20246 Hamburg, Germany	Hamburg, Germany	Clinical Investigator	
Priska	Hakenberg			Department of Surgery, University Medical Center Mannheim, Medical Faculty Mannheim, Heidelberg University, Theodor-Kutzer-Ufer 1-3, 68167 Mannheim, Germany	Mannheim, Germany	Clinical Investigator	
Erik	Rasbach			Department of Surgery, University Medical Center Mannheim, Medical Faculty Mannheim, Heidelberg University, Theodor-Kutzer-Ufer 1-3, 68167 Mannheim, Germany	Mannheim, Germany	Clinical Investigator	
Dorothee	Sturm			Department of Surgery, University Medical Center Mannheim, Medical Faculty Mannheim, Heidelberg University, Theodor-Kutzer-Ufer 1-3, 68167 Mannheim, Germany	Mannheim, Germany	Study Coordinator	

Supplemental Online Content: Nonauthor Collaborators

*Indicates required information. Only first name, last name, and suffix will appear in PubMed.

*First Name and Middle Initial(s)	*Last Name	*Suffix (eg, Jr, III)	Academic Degrees	Institution	Location (city, state/province, country)	Role or Contribution, eg, chair, principal investigator	Group (if more than 1 Group listed in the byline) and/or Subgroup (eg, Steering Committee)
Hany	Ashmawy			Department of Surgery A, General, Visceral, Thoracic, and Pediatric Surgery, University Hospital Düsseldorf and Heinrich Heine University Düsseldorf, Moorenstr. 5, 40225 Düsseldorf, Germany	Duesseldorf, Germany	Clinical Investigator	
Levent	Dizdar			Department of Surgery A, General, Visceral, Thoracic, and Pediatric Surgery, University Hospital Düsseldorf and Heinrich Heine University Düsseldorf, Moorenstr. 5, 40225 Düsseldorf, Germany	Duesseldorf, Germany	Clinical Investigator	
Sascga	Vaghiri			Department of Surgery A, General, Visceral, Thoracic, and Pediatric Surgery, University Hospital Düsseldorf and Heinrich Heine University Düsseldorf, Moorenstr. 5, 40225 Düsseldorf, Germany	Duesseldorf, Germany	Clinical Investigator	
Benjamin	Albers			Asklepios Clinic Langen, Department of General, Visceral and Thoracic Surgery, Röntgenstr. 20, 63225 Langen, Germany	Langen, Germany	Clinical Investigator	
Marcus	Mittag		MD	Asklepios Clinic Langen, Department of General, Visceral and Thoracic Surgery, Röntgenstr. 20, 63225 Langen, Germany	Langen, Germany	Clinical Investigator	
