# Supplementary material for: Intraoperative Wound Irrigation for the Prevention of Surgical Site Infection After Laparotomy: A Randomized Clinical Trial by CHIR-Net
Source: JAMA Surg. 2024 Feb 21;159(5):484–92. doi: 10.1001/jamasurg.2023.7985 (PMC10882507; doi:10.1001/jamasurg.2023.7985)
Supplement: Supplement 4. — Data sharing statement [file jamasurg-e237985-s004.pdf]

Data Sharing Statement

Mueller. Intraoperative Wound Irrigation for the Prevention of Surgical Site Infection After Laparotomy. *JAMA Surg*. Published February 21, 2024. doi:10.1001/jamasurg.2023.7985

Data
Data available: No

Additional Information
Explanation for why data not available: Data collected for the study, including individual participant data or a data dictionary, will not be made available to others. The study protocol is available, having been published in a peer-reviewed journal and in the German clinical trials register DRKS (DRKS number 00012251) (www.drks.de). The results of the study are accessible in the European clinical trials register (EUDRA-CT number 2017-000152-26) (https://www.clinicaltrialsregister.eu/ctr-search/trial/2017-000152-26/results).
